# Organometallic Complexes with an Indolo[2,3‑*c*]Quinoline-Derived Ligand: From Structural Features and Solution Speciation to Nanoformulation for Enhanced Therapeutic Potential

**DOI:** 10.1021/acs.inorgchem.6c01228

**Published:** 2026-05-25

**Authors:** Tamás Pivarcsik, Egon F. Várkonyi, János P. Mészáros, Orsolya Dömötör, Márta Nové, Gabriella Spengler, Nóra V. May, Petra Bombicz, Christopher Wittmann, Felix Bacher, Vladimir B. Arion, Edit Csapó, Éva A. Enyedy

**Affiliations:** † Department of Molecular and Analytical Chemistry, 37442 University of Szeged, Dóm tér 7-8, Szeged H-6720, Hungary; ‡ MTA-SZTE Lendület “Momentum” Noble Metal Nanostructures Research Group, University of Szeged, Rerrich B. tér. 1, Szeged H-6720, Hungary; § Department of Physical Chemistry and Materials Science, University of Szeged, Rerrich B. tér 1, Szeged H-6720, Hungary; ∥ Department of Medical Microbiology, Albert Szent-Györgyi Health Center and Albert Szent-Györgyi Medical School, University of Szeged, Semmelweis u. 6, Szeged H-6725, Hungary; ⊥ Centre for Structural Science, HUN-REN Research Centre for Natural Sciences, Magyar Tudósok krt. 2, Budapest H-1117, Hungary; # Institute of Inorganic Chemistry, Faculty of Chemistry, 27258 University of Vienna, Währinger Str. 42, Vienna 1090, Austria; ¶ Department of Inorganic Polymers, “Petru Poni” Institute of Macromolecular Chemistry, Aleea Gr. Ghica Voda 41 A, Iasi 700487, Romania

## Abstract

Synthesis and comprehensive solid and solution phase characterization of Ru­(II)­(η^6^-*p*-cymene), Os­(II)­(η^6^-*p*-cymene), and Rh­(III)­(η^5^-C_5_Me_5_) complexes of an indolo­[2,3-*c*]­quinoline-derived Schiff base compound (IQPMA) and its simpler analogue (DIPMA), both bearing a bidentate (N,N) chelating motif, are reported. The complexes exhibit enhanced aqueous solubility compared to the free ligands. The structures of five half-sandwich complexes were determined by single-crystal X-ray diffraction. A correlation analysis revealed that, despite the highly similar coordination geometry, conformational variations are primarily governed by steric and electronic interactions between the aromatic ring systems. Complexation of IQPMA and DIPMA with Rh­(III)­(η^5^-C_5_Me_5_) promoted ligand hydrolysis, while the corresponding Ru­(II) and Os­(II) organometallic complexes remained stable at physiological pH (7.4). These latter complexes showed very slow aquation kinetics and strong binding to human serum albumin mediated by intermolecular interactions. IQPMA, its complexes, and the DIPMA complexes exhibited weak-to-moderate cytotoxicity, and the Ru­(II)­(η^6^-*p*-cymene) complex of IQPMA was selectively active toward breast adenocarcinoma MCF-7 cells. Metal coordination significantly enhanced antibacterial efficacy against Gram-positive strains and inhibition of biofilm formation. To improve bioavailability, the Ru­(II)­(η^6^-*p*-cymene) complexes were encapsulated into asolectin-derived liposomes (∼200 nm) with high encapsulation efficiency and colloidal stability. Importantly, cytotoxicity assays confirmed that nanoformulation preserved the biological activity of the metal complexes.

## Graphical abstract

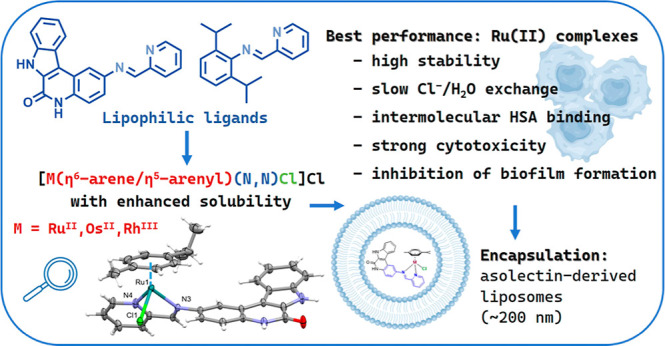

## Introduction

Paullones, structurally derived from the indolo­[3,2-*d*]­benzazepine scaffold, are prominent pharmacologically active heterocyclic compounds, known for their potent inhibitory effects on various kinase proteins, including cyclin-dependent kinase,^1–5^ glycogen synthase kinase-3β,^3,4,6^ and mitochondrial malate dehydrogenase.^4,7^ Certain paullone derivatives and their Cu­(II) complexes were also recognized for targeting the human R2 ribonucleotide reductase protein,^8^ while the related indole-fused heterocycles, indolo­[2,3-*d*]­quinolines (also known as latonduines), act as effective microtubule-targeting agents.^9^ Indoloquinolines feature a fused system of quinolin-2­(1H)-one and indole moieties, both of which are common motifs in biologically active compounds.^10–13^ Certain indoloquinolines were reported to exert antimicrobial activity as well.^14,15^ Structural modifications of the indoloquinoline backbone primarily involve isomerism (e.g., indolo­[3,2-*c*]­quinolines or the structurally related indolo­[2,3-*c*]­quinolines), as well as variations in ring sizes, resulting in seven-membered (paullones), eight-membered (indolo­[2,3-*e*]­benzazocines), and nine-membered (indolo­[2,3-*f*]­benzazonines) analogues.^16–21^ Indolo­[2,3-*c*]­quinolines exhibited stronger cytotoxicity than their paullone counterparts, highlighting the significant impact of replacing the azepine ring with a six-membered pyridine ring.^19^ Moreover, indoloquinolines have been shown to act as superior to paullones DNA intercalators, due to their planar molecular structure resulting from the conjugated and extended aromatic ring system.^19^

However, the poor aqueous solubility and bioavailability of these scaffolds remain major limitations in their use as drugs. In our previous studies, we addressed this issue by introducing various metal binding sites at different positions on the core structure, thereby generating bidentate or tridentate chelating moieties. Primarily, sp^2^-hybridized *N*-donor atoms were incorporated to increase ligand solubility upon complexation, as well as to improve cytotoxic activity and thermodynamic stability. A variety of Cu­(II), Zn­(II) coordination complexes,^13,17–21^ as well as Ru­(II) and Os­(II) arene metal complexes were developed,^21,22^ most of which displayed superior anticancer properties compared to the corresponding proligands. Indeed, certain Cu­(II) complexes of indolo­[2,3-*c*]­quinoline-derived Schiff bases exhibited markedly improved aqueous solubility as well.^13^

The developed Ru­(II)­(η^6^-*p*-cymene) and Os­(II)­(η^6^-*p*-cymene) metal complexes of indolo­[3,2-*c*]-quinolines exhibited IC_50_ values in the micromolar range against a panel of human cancer cell lines,^22–25^ showing a 5- to 16-fold increase in cytotoxicity compared to the uncoordinated ligand precursors, thus underscoring their potential as anticancer agents. Furthermore, efforts have also been made to enhance cytotoxicity and improve cancer cell targeting via conjugation with human serum albumin (HSA), which exacerbated the cytotoxicity significantly.^26^

While comprehensive solution equilibrium studies on Cu­(II) complexes of indoloquinolines and related compounds have been already reported,^13,17,19,20^ no solution phase chemical data are available for such organometallic half-sandwich complexes. Therefore, in this work, our aim was to develop and characterize the Ru­(II)­(η^6^-*p*-cymene) (RuCym), Os­(II)­(η^6^-*p*-cymene) (OsCym), and Rh­(III)­(η^5^-C_5_Me_5_) (RhCp*) complexes of an indolo­[2,3-*c*]-quinoline-based compound [2-((pyridin-2-ylmethylene)­amino)-5*H*-indolo­[2,3-*c*]­quinolin-6­(7*H*)-one (IQPMA, Chart 1)] possessing an (*N,N*) donor set and to investigate their cytotoxic and antibacterial activity. The coordination mode of this ligand is similar to that of indolo­[3,2-*c*]-quinolines,^22,23^ although the indole is attached to the quinoline through different carbon atoms, altering the spatial orientation of the heterocyclic core. The solution phase properties of the new compounds, such as lipophilicity, stability, coligand exchange processes, and interaction with HSA, are also comprehensively investigated and discussed. Two simpler model ligands, namely, 2,6-diisopropyl-*N*-(pyridin-2-ylmethylene)­aniline (DIPMA) and *N*-(pyridin-2-ylmethylene)­aniline (PMA), containing the same type of (N,N) donor set as IQPMA (Chart 1), were also involved. The compounds were tested on human cancer cells, and the most promising candidates, the Ru­(II)­(η^6^-*p*-cymene) complexes of IQPMA and DIPMA, were selected for further nanoformulation studies using asolectin (ASO)-based liposomes. A biodegradable poly­(lactic-*co*-glycolic acid) (PLGA)-based system was also explored as an alternative nanocarrier.

**Chart 1:**
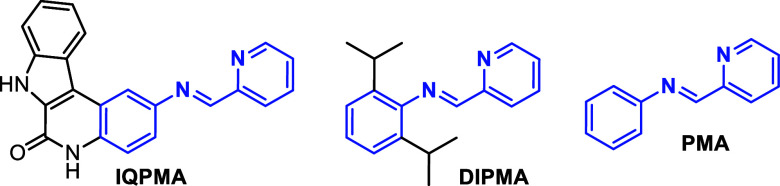
Chemical structures of the bidentate ligands bearing the (*N,N*) donor set: IQPMA, DIPMA, and PMA.

## Results and Discussion

### Synthesis of IQPMA Ligand and Its Characterization

IQPMA was synthesized in six steps (Scheme 1). Starting from 2-amino-5-nitrobenzophenone (**A**), chloroacetylation, followed by azide substitution, afforded intermediate **C**, which underwent cyclization to quinolinone **D** and subsequently to indoloquinoline **E**. Final reduction of the nitro group yielded 2-aminoindolo-[2,3-*c*]­quinoline-6-one (**F**). Condensation of **F** with 2-formylpyridine afforded the Schiff base HIQPMA.

**Scheme 1:**
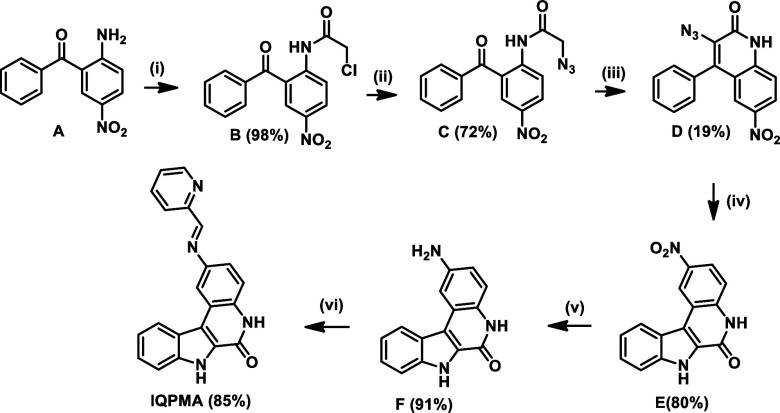
Multistep Synthesis of IQPMA; Reagents and Conditions: (i) ClCH_2_COCl, CHCl_3_, Reflux, 30 min; (ii) NaN_3_, DMF, H_2_O, 60 °C, 30 min; (iii) 40% NaOH, Ethanol, 70 °C, 16 h; (iv) Toluene, Reflux, 2 h; (v). Pd/C (10%), H_2_, THF_deoxygenated_, 3 bar, RT, 20 h; (vi): 2-Formylpyridine, Ethanol, 85 °C, 16 h; Yields are Shown in the Parentheses

The positive ion electrospray ionization mass spectrometry (ESI-MS) measurement for IQPMA showed a peak at *m*/*z* 339.16 attributed to [M + H^+^]^+^. The ^1^H and ^13^C NMR spectra of IQPMA were in accordance with the *C*_1_ molecular symmetry of the Schiff base (Figures S1–S7). The purity of the compound required for biological investigations was confirmed by elemental analysis. The compound was also characterized by single crystal X-ray diffraction (SC-XRD) crystallography (vide infra).

### Synthesis of the Half-Sandwich Complexes of IQPMA and DIPMA and Their Characterization

For the synthesis of the half-sandwich complexes, IQPMA and DIPMA were dissolved in dichloromethane or chloroform, to which a half-equivalent of metal precursor ([Ru­(η^6^-*p*-cymene)­Cl_2_]_2_, [Os­(η^6^-*p*-cymene)­Cl_2_]_2_, or [Rh­(η^5^-C_5_Me_5_)­Cl_2_]_2_, dissolved in the same solvent or in methanol) was added. Notably, the analogous organometallic Ru­(II) and Os­(II) complexes of indolo­[3,2-*c*]-quinolines were prepared differently, namely, using a one-pot three-component synthesis from the corresponding 2-aminoindoloquinoline derivative, 2-formylpyridine, and the given metal dinuclear precursor.^23^ After 24 h of stirring, the solvent was partially evaporated, and a small amount of diethyl ether was added to induce precipitation. The products were filtered, washed with diethyl ether, and dried in an oven at 45 °C for 4 h. This procedure was consistently followed for each complex with IQPMA and DIPMA.

Three novel complexes, [RuCym­(IQPMA)­Cl]­Cl (**1a**), [OsCym­(IQPMA)­Cl]Cl (**2a**), and [RhCp*­(IQPMA)­Cl]­Cl (**3a**), with one chlorido coligand and one chloride counterion were synthesized, in addition to [RuCym­(DIPMA)­Cl]Cl (**4a**), [OsCym­(DIPMA)­Cl]Cl (**5a**), and [RhCp*­(DIPMA)­Cl]Cl (**6a**) (Figure 1). The structures and purity of the synthesized complexes were confirmed by ^1^H and ^13^C NMR spectroscopy (Figures S8–S19), elemental analysis, and ESI-MS. In the ^1^H NMR spectra, the formation of the half-sandwich complexes is clearly evidenced by the appearance of a new set of signals distinct from those of both the free ligand and the organometallic precursor. Notably, no signals attributable to free ligand or metal precursor were detected, indicating the formation of pure products. Chemical shifts were observed for the ligand and the organometallic fragment ^1^H resonances, consistent with coordination. In the ESI mass spectra, the dominant peaks correspond to the expected [M­(arene)­(ligand)­Cl]^+^ ions, further supporting the proposed composition of the prepared complexes. Additionally, single crystals suitable for SC-XRD analysis (vide infra) were obtained for five complexes (**1b**–**4b**, **6b**, Figure 1), in which the outer sphere chloride was replaced by PF_6_^–^ for the better crystallization conditions. All experimental data confirmed the bidentate coordination of the neutral ligand to the metal center via an (*N,N*) donor set, with a chlorido coligand completing the coordination sphere. It should also be noted that syntheses of **6b**,^27^ [RuCym­(DIPMA)­Cl]­BF_4_,^28^ [RuCym­(DIPMA)­Cl]­PF_6,_ ^29^ and [OsCym­(DIPMA)­Cl]­PF_6_ ^30^ were reported previously.

**Figure 1:**
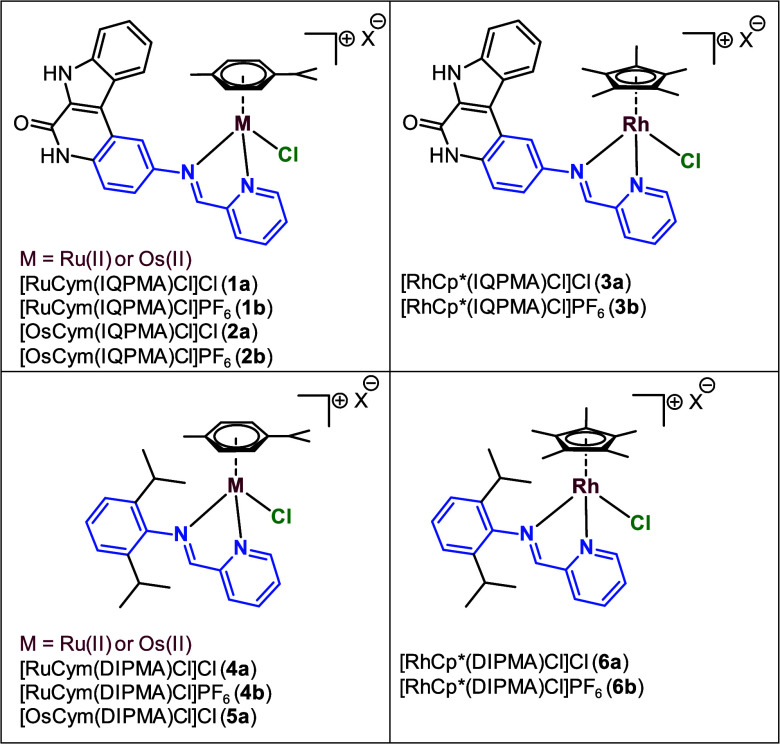
Chemical structures of the half-sandwich Ru­(II)­(η^6^-*p*-cymene), Os­(II)­(η^6^-*p*-cymene), and Rh­(III)­(η^5^-C_5_Me_5_) complexes of IQPMA and DIPMA. Solvent molecules found in the crystals are not shown. X^–^ denotes the counteranion: Cl^–^ or PF_6_^–^ as indicated in the name of the complexes.

### Structural Studies of IQPMA and DIPMA Organometallic Complexes by SC-XRD

An orange, plate-shaped single crystal of IQPMA (**I**) was grown by layering diethyl ether over its DMF solution in an NMR tube. Crystal data and structure refinement parameters are collected in Table S1. Selected bond distances and angles are collected in Table S2. IQPMA crystallized in the monoclinic space group *P*2_1_/*n*. An ORTEP view of the molecule is depicted in Figure 2, and the unit cell is shown in Figure S20. The IQPMA molecule is not completely planar, as the pyridine ring makes an angle of 33.95(12)° with the fused aromatic ring system (Figure S21). Details of the crystal packing, including the herringbone arrangement, π–π stacking interactions, and hydrogen bonding, are provided in the captions of Figures S22 and S23.

**Figure 2:**
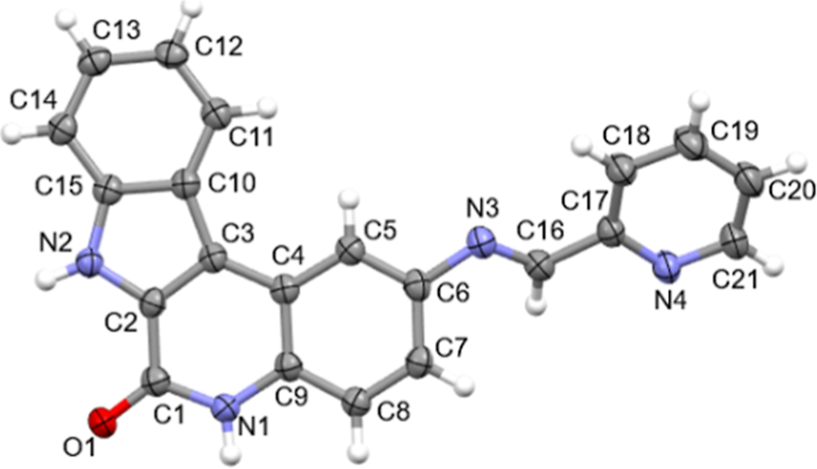
ORTEP view of IQPMA (**I**) with thermal ellipsoids drawn at the 50% probability level.

The organometallic complexes with IQPMA and DIPMA were crystallized under various experimental conditions (see Experimental Section) to give single crystals of XRD quality of [RuCym­(IQPMA)­Cl]­PF_6_×DMF (**1b**×DMF), [OsCym­(IQPMA)­Cl]­PF_6_×DMF (**2b**×DMF), [RhCp*­(IQPMA)­Cl]­PF_6_×DMF (**3b**×DMF), [RuCym­(DIPMA)­Cl]­PF_6_ (**4b**), and [RhCp*­(DIPMA)­Cl]­PF_6_ (**6b**) (Figure 3).

**Figure 3:**
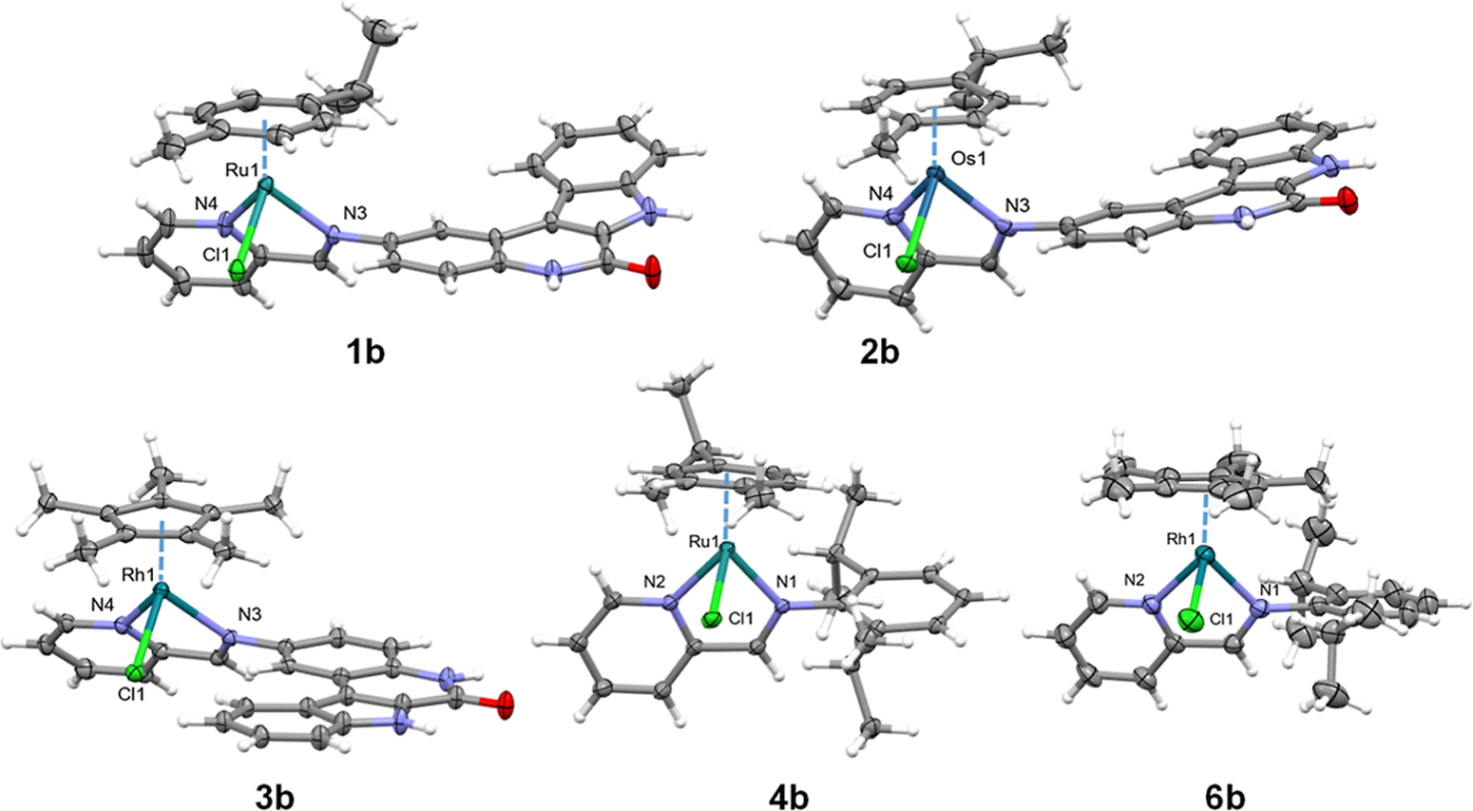
ORTEP view of [RuCym­(IQPMA)­Cl]­PF_6_ ×DMF (**1b** ×DMF), [OsCym­(IQPMA)­Cl]­PF_6_ ×DMF (**2b** ×DMF), [RhCp*­(IQPMA)­Cl]­PF_6_ ×DMF (**3b** ×DMF), [RuCym­(DIPMA)­Cl]­PF_6_ (**4b**), and [RhCp*­(DIPMA)­Cl]­PF_6_ (**6b**). Interstitial DMF molecules and counterions are omitted for clarity. Thermal displacement ellipsoids are drawn at the 50% probability level.

Crystals of **1b** and **2b** contained unidentifiable residual electron densities in the holes, which were assigned to disordered diethyl ether, as this solvent was used for their crystallization. Crystal data and structure refinement details for **1b** and **2b** are collected in Table S1 and those for **3b**, **4b,** and **6b** in Table S3. Selected bond distances and angles are collected in Table S4. In all cases, the metal adopts a pseudo-octahedral (“piano-stool”) geometry, where, in addition to the η^6^ and η^5^ binding of *p*-cymene (Cym) and Cp* ring, respectively, pyridine and amine nitrogen atoms of IQPMA or DIPMA are coordinated to the metal, forming a five-membered chelate ring, and a chlorido coligand completes the coordination sphere. The overlaid structures of the RuCym, OsCym, and RhCp* complexes are shown in Figure 4. The +1 global charge of the complex is counterbalanced by a PF_6_^–^ counteranion, and one DMF molecule also occupies the interstitial space in the crystal structures of **1b**, **2b,** and **3b**. Further details of the unit cells, packing arrangements, and intermolecular interactions are given in the captions of Figures S24–S26. Interestingly, the symmetrical N2–H2···O1 H-bond intermolecular interactions between the IQPMA ligands present in the free ligand (**I**) are also found in the crystal structures of RuCym, OsCym, and RhCp* complexes with IQPMA (Figure S26), implying that this H-bond operates as a significant driving force in the formation of the crystal structure. Further crystallographic details, including solvent-filled channels, unit-cell packing along the main crystallographic axes, intermolecular interactions, and conformational comparisons of the complexes, are provided in the Supporting Information (Figures S27–S31).

**Figure 4:**
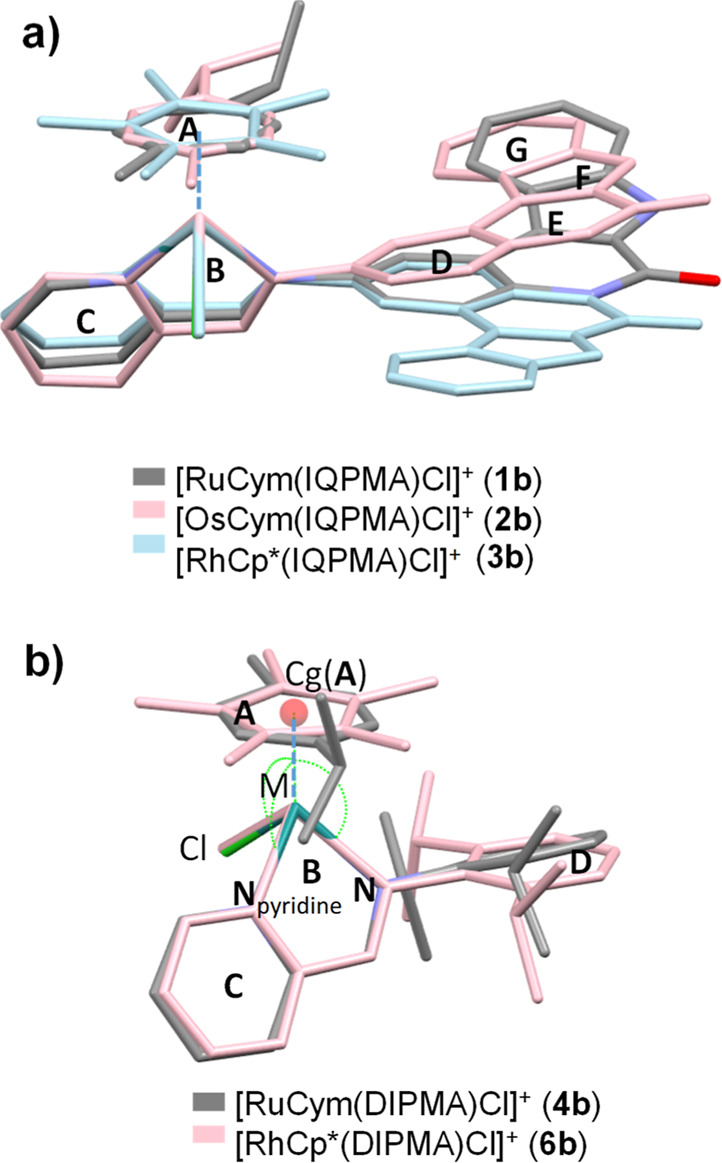
Overlaid structures of organometallic complexes in crystals of (a) **1b**-**3b** and (b) **4b** and **6b** with ring numbers. The metal atoms (M), Cl, N_pyridine_, and N atoms are overlaid. Ring and atom numbering are shown in Table S4.

Since PMA, IQPMA, and DIPMA ligands all contain an aromatic ring attached to the metal ion via the (N,N) donor set and an N–C single bond, we tried to understand the factors governing their conformation. In this context, the conformations of our complexes (Figures 3 and 4) were compared with previously reported crystal structures of organometallic complexes with DIPMA and PMA deposited with the Cambridge Structural Database (CSD) (2025.1.1): [RuCym­(DIPMA)­Cl]­BF_4_ (ref. code ROLDIJ^28^), [OsCym­(DIPMA)­Cl]­PF_6_ (ref. code TELBAQ^30^), and [RhCp*­(DIPMA)­Cl]­PF_6_×CH_2_Cl_2_ (ref. code ACUDAH^27^) and three solvate IrCp* complexes: [IrCp*­(DIPMA)­Cl]­PF_6_×toluene (ref. code DOSXAN^31^), [IrCp*­(DIPMA)­Cl]×4-methylbenzene-1-sulfonate (ref. code LIYSAQ^32^), and [IrCp*­(DIPMA)­Cl]­PF_6_×CH_2_Cl_2_ (ref. code SEPWUI^33^); together with PMA complexes: [RuCym­(PMA)­Cl]­PF_6_×acetone (ref. code DEQNAS^34^), [RhCp*­(PMA)­Cl]­PF_6_ (ref. code AQECOQ^35^, and [IrCp*­(PMA)­Cl]­PF_6_ (ref. code AQECIK^35^). The relevant bond distances and angles of the coordination spheres, together with ring plane angles for all 14 complexes, are collected in Tables S4 and S5, and statistical analysis was performed to reveal correlations and similarities among the investigated half-sandwich complexes. For analysis, the following metric parameters were used (Figure 4b): ligand–metal bond distances (M–Cl, M–N, and M–N_pyridine_) and the distance between the center of gravity of the Cp*/Cym ring (Cg­(A)) and the metal ion (Cg­(A) – M), bond angles enclosed by the Cg­(A) and ligand donor atoms (Cg­(A) – M–Cl, Cg­(A) – M–N, and Cg­(A) – M–N_pyr_), and the angles between the ligand donor atoms and Cl^–^ ion (Cl–M–N, Cl–M–N_pyridne_, and N–M–N_pyridine_). In addition, the angles enclosed by three ring planes were also examined: the angle enclosed by the plane of the Cp*/Cym ring and the coordinating pyridine ring plane (BC), the plane of the pyridine and the associated aromatic ring (D), and the angle enclosed by the planes of rings A and D (Table S4, Figure 4a). Cluster analysis (CA) was performed on the standardized data set using the software Statistica,^36^ where samples are grouped based on similarities without considering the information about the class membership. The tree diagram obtained by CA indicates that, based on the type of ligands, the structures can be divided into two main groups: complexes formed with DIPMA ligands and with IQPMA or PMA ligands (Figure S32). In the tree structure, these groups are further divided into smaller subgroups according to the type of metal ion, i.e., RuCym/OsCym complexes are separated from RhCp*/IrCp* complexes for both ligand groups. However, despite these inherent differences in the coordination environment and electronic properties, the applied statistical approach allows for the identification of general trends and similarities across the entire series. Based on the conformational data examined, the greatest similarity is found between the [IrCp*­(PMA)­Cl]­PF_6_ (AQECIK^35^) and [RhCp*­(PMA)­Cl]­PF_6_ (AQECOQ^35^) complexes, which show the closest relationship in the tree diagram. In addition to these, there is also a fairly high similarity between the two solvate structures of [IrCp*­(DIPMA)­Cl]­PF_6_ (SEPWUI^33^ and DOSXAN^31^) complexes, and between pairs [OsCym­(DIPMA)­Cl]­PF_6_ (TELBAQ^30^) and [RuCym­(DIPMA)­Cl]­PF_6_ (**4b**), as well as [RuCym­(PMA)­Cl]­PF_6_ (DEQNAS^34^) and [RuCym­(IQPMA)­Cl]­PF_6_ (**1b**).

Correlation analysis was also performed for data collected in Table S4 and significant correlations (Pearson’s *R* value > 0.7) among the structural parameters were found (Table S6). It is known that the M–Cg distance is much smaller in Ru­(II)/Os­(II) half-sandwich complexes than in Rh­(III)/Ir­(III) ones, so this value divides the data into two groups, leading to false correlation data. However, it is noteworthy that the Cg-M distance increases slightly with the angle between the larger D-BC ring planes within both the Ru/Os and Rh/Ir groups (Figure 5a).

**Figure 5:**
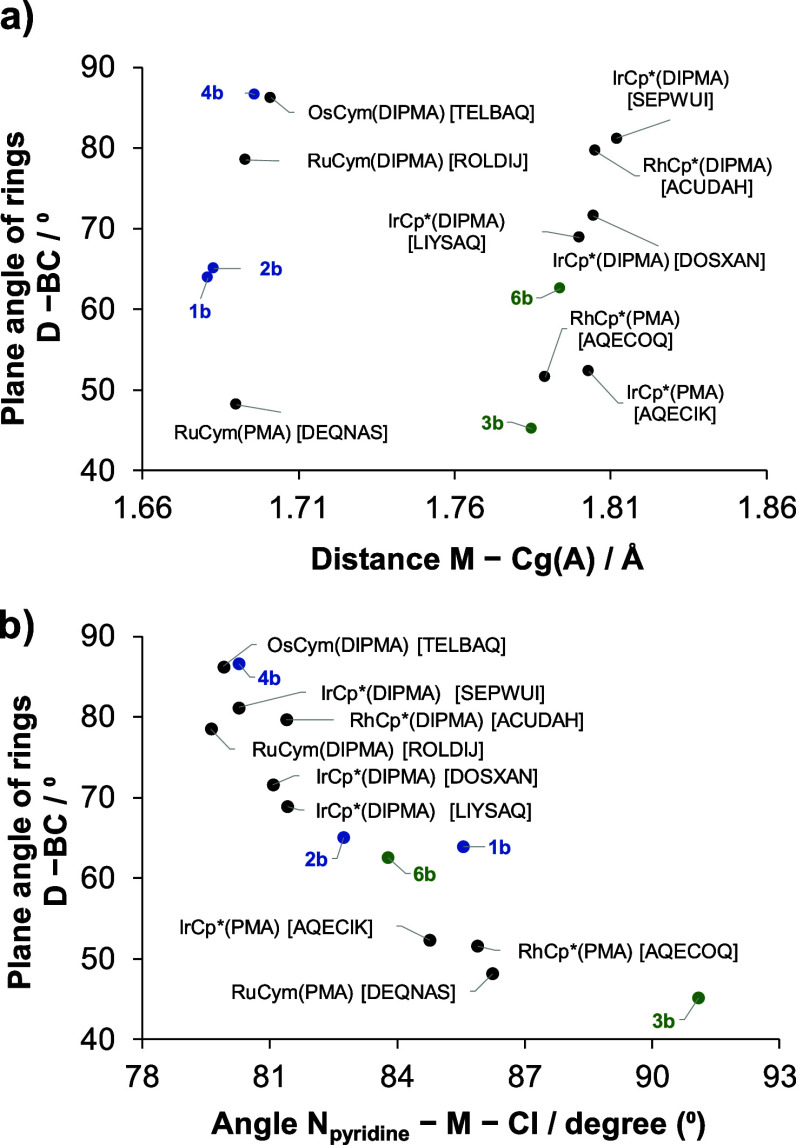
Correlation diagram between ring plane angle D–BC (a) with distance M–Cg­(A) and (b) with angle N_pyridine_–M–Cl for 14 organometallic complexes based on their crystallographic data. Data are taken from refs [ ^27,28,30–35^ ].

The results obtained may indicate a steric effect of the ring conformations that influence the Cg–M distance. Furthermore, it appears that this D–BC angle has an opposite effect to the N_pyridine_–M–Cl angle (*R* = – 0.9041), regardless of the type of metal ion (Figure 5b). Details of the correlations between geometric parameters and the resulting conformational effects, suggesting their predominantly intramolecular origin, are provided in the captions of Figures S33–S35. Based on the results of the correlation analysis, we assume that the conformational effects are determined within the complex and do not depend on secondary interactions occurring in the crystal structure.

Overall, it can be concluded that, apart from the metal–Cp*/Cym distance, the coordination mode in the “piano-stool” organometallic complexes of PMA, IQPMA, and DIPMA with Ru­(II), Rh­(III), Os­(II), and Ir­(III) is very similar. At the same time, the angle between the two aromatic systems of the ligands can vary widely (D–BC angle is between 48.2° and 86.6°). Larger angles can be seen for DIPMA and smaller angles for IQPMA and PMA. The smaller angle is likely driven by conjugation, as also seen in the crystals of the free IQPMA. However, steric constraints imposed by the Cp*/Cym ring force the D ring to adopt an orientation nearly parallel to it (A-D angle <21.5°), which in turn dictates the coordination geometry of the BC ring, independent of the metal type.

### Stability and Solution Equilibrium Processes

#### Compounds PMA, DIPMA, and IQPMA

To elucidate the solution behavior of the Schiff-base ligands (IQPMA and DIPMA), their simplest and most soluble structural analogue, PMA (Chart 1), was first investigated. Since these compounds, containing the CH = N moiety, may undergo hydrolysis in water, the stability and light-sensitivity of the model compound PMA were monitored over time at pH 0.7, 7.4, and 13.0 by UV–visible (UV–vis) spectrophotometry at 200 μM concentration (Figure S36). UV–vis spectra indicate that PMA is stable under acidic and neutral conditions, while changes occur under basic conditions, most likely due to hydrolysis. Accordingly, titrations were performed in the pH range 2.5–11.4. Further details are provided in the caption of Figure S37. It should be noted that the absorbance–pH curves exhibit wavelength-dependent inflection points at pH < 6, suggesting the presence of isomeric species. To clarify this behavior, ^1^H NMR spectra were recorded at various pH values (Figures S38 and S39), confirming the formation of two isomers (*Z* and *E* forms) and significant hydrolysis at pH > 11 (see further explanation in the legend of the figure). The distinct sets of resonances corresponding to the two isomers show different pH dependences and varying intensity ratios, which allowed the determination of their microscopic p*K*_a_ values (associated with the pyridinium nitrogen) as well as their actual molar fractions. Specifically, the p*K*_a_ = 4.41 ± 0.02 microconstant was obtained for the *E* isomer, whereas the value for the *Z* isomer could merely be estimated (p*K*_a_ ∼ 2.5). The higher p*K*_a_ value of the *E* isomer is attributed to the presence of an intramolecular hydrogen bond between the pyridinium NH^+^ and the imine nitrogen (Figure 6b). As shown in Figure 6, the molar fractions indicate that at pH < 4.6, the *E* isomer predominates (HL_*E*_^+^), most probably due to the stabilization by this intramolecular hydrogen bond. With increasing pH, the formation of the *Z* isomer becomes more pronounced, and at neutral pH their ratio is *x*(*E*):*x*(*Z*) ∼ 2:3. On the basis of the microconstants, the macroconstant characterizing the entire system is p*K*_a_ = 4.0.

**Figure 6:**
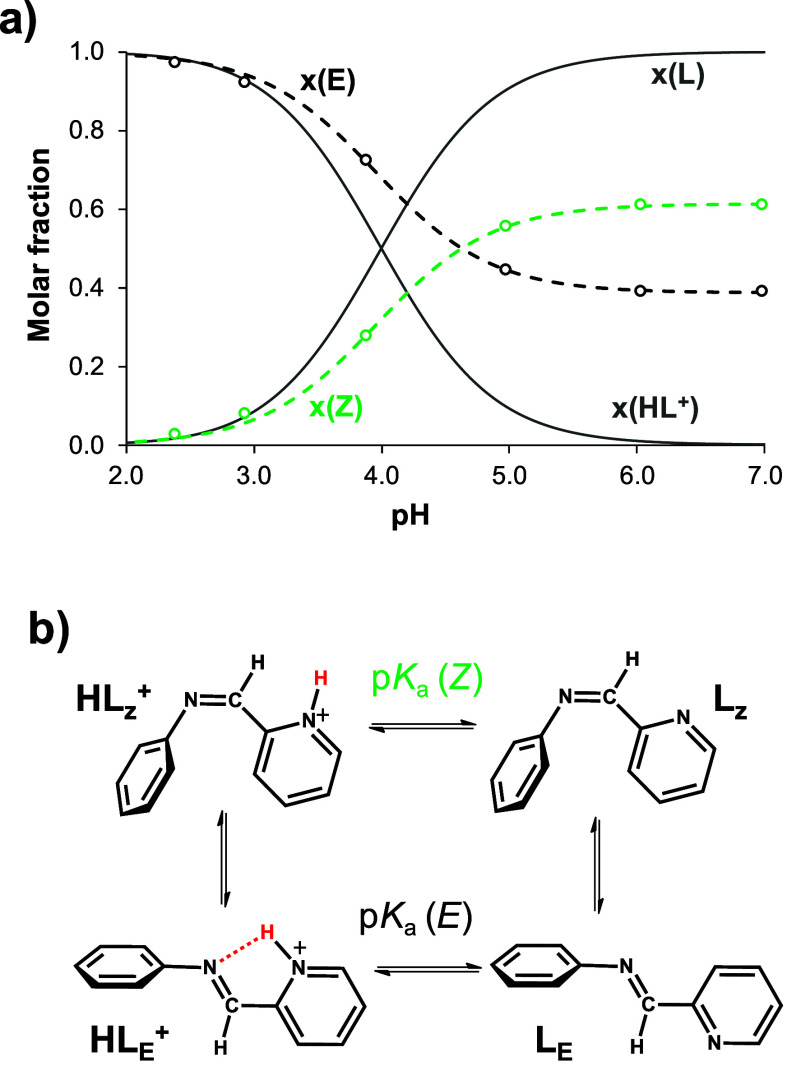
(a) Molar fraction of the HL^+^ and L species of PMA plotted against the pH (solid lines) together with the molar fraction of the *Z* and *E* isomeric forms (dashed lines) and p*K*_a_ microconstants calculated from the ^1^H NMR spectra recorded at the different pH values (see Figure S38). The symbols (o) denote the molar fractions calculated directly from the ^1^H NMR spectra at the given pH value. (b) The chemical equilibria for the deprotonation and isomerization processes. {*c*_PMA_ = 200 μM; *I* = 0.10 M (KCl); *T* = 25.0 °C}.

The stability of DIPMA and IQPMA under acidic (pH 1.0) and physiological (pH 7.4) conditions was also examined by UV–vis spectrophotometry (10 μM, 1% (*v/v*) DMSO/H_2_O) and ^1^H NMR spectroscopy (300 μM, 50% (*v*/*v*) DMSO-*d*_6_/PBS’, pH 7.4) (Figures S40 and S41). Notably, the stock solutions were prepared in pure DMSO due to their limited aqueous solubility. For direct comparison, the spectral changes were also monitored for PMA at pH 7.4 when the sample was obtained from a DMSO stock solution diluted by the aqueous buffer (Figure S36c). The ^1^H NMR spectra recorded for the DMSO-*d*_6_ solution of IQPMA and DIPMA (in addition to PMA) reveal that the compounds display no changes for 24 h and exist in a single isomeric form, most likely the *E* isomer (Figure S42). DIPMA and IQPMA in the 1% (*v*/*v*) DMSO/H_2_O solvent mixture remained stable for 24 h at pH 1.0 (Figures S40a and S41a). In addition, IQPMA proved to be fluorescent under these conditions (Figure S43a). On the contrary, spectral changes were observed at pH 7.4 in both the UV–vis and ^1^H NMR spectra over the same period. However, the extent of these changes differs for the three compounds. In the case of DIPMA and PMA, the ^1^H NMR spectra recorded at pH 7.4 remained unchanged over time; however, DIPMA appears in the 50% (*v*/*v*) DMSO-*d*_6_/PBS solution exclusively as a single isomer (most probably *E*), as the *Z* isomer is sterically disfavored by the 2,6-diisopropyl moieties. On the contrary, the UV–vis absorbance spectra of both DIPMA and PMA showed changes under the applied conditions (Figures S40b and S36c). We attribute the changes to a time-dependent shift in the *Z*/*E* ratio upon strong dilution of the DMSO stock solution with aqueous buffer, while the extent of the UV–vis spectral changes was even greater with IQPMA (Figure S41b). The forming species for IQPMA is highly fluorescent in the visible wavelength range; the initial form is much less fluorescent, and the detected low fluorescence is probably also due to the forming product (Figure S43a,b). Notably, the precipitate also appeared in the case of DIPMA and IQPMA under the conditions used, which makes the interpretation of the spectral changes more difficult.

#### Organometallic Complexes with PMA, DIPMA, and IQPMA

In order to understand the behavior of pharmacologically active metal complexes in biofluids, as well as their pharmacokinetics and mechanisms of action, it is crucial to characterize their solution phase properties such as stability, ligand-exchange processes, and lipophilicity. For the stability assays, the complexes **1a**–**6a** were dissolved in various media. The PMA complexes were not isolated since they were only used for comparison and were prepared in situ by mixing equimolar solutions of the ligand and the corresponding metal precursor (at pH ∼ 3 to prevent hydrolysis of either of them, similarly as done in our previous works^37,38^ ). After complete complex formation, the pH was adjusted to the desired value by the addition of acid (HCl), buffer (PBS’ or phosphate), or base (KOH) (Figures S44–S53). During the monitored 24 h, or even longer times, the ^1^H NMR and UV–vis spectra of all studied organometallic complexes remained unchanged at pH 1.0 except the case of complex **6a** (Figure S47a). All RuCym and OsCym complexes were also stable at pH 7.4, as no signs of complex dissociation or ligand hydrolysis were observed. The spectra of the RuCym complexes **1a** and **4a** also remained unchanged in the pH range 2–9 (Figure S49). On the contrary, the RhCp* complexes of PMA and IQPMA ligands exhibited slow but significant spectral changes, e.g., for the PMA 19% decomposed product after 3 days (Figures S50 and S51b), at pH 7.4, the pH where the ligand alone was found to be stable during this period. The aqueous stability of [RhCp*­(DIPMA)­Cl]­PF_6_ (**6b**) was also studied at pH 7.2 in 30% (*v*/*v*) DMSO/70% (*v*/*v*) PBS buffer at 37 °C by Hu et al., and only the Cl^–^ → H_2_O coligand exchange was reported.^27^ The RuCym and RhCp* complexes of PMA were further studied at pH 9.4, and hydrolytic processes occurred in all cases (faster for the RhCp* complexes), resulting in the formation of various products (Figures S52 and S53). In all, these results suggest that RhCp* can facilitate ligand hydrolysis, and complexation with the resulting amine hydrolysis product is also possible.

The stability of the complexes **1a**–**6a** was further monitored at pH 7.4 in Eagle’s minimum essential medium (EMEM), a biologically relevant matrix. The RuCym and OsCym complexes showed no or only minor spectral changes in this medium and were similar to those of the complexes alone in the buffered medium (Figure S54). However, the RhCp* complexes exhibited significant spectral shifts, due to the accelerated hydrolysis of the Schiff base ligand and/or possible interaction with medium components (Figure S55).

When [M­(η^6^-arene/η^5^-arenyl)­(ligand)­Cl]^+^ complexes are dissolved in water, the chlorido coligand is assumed to undergo partial substitution by water, which affects the actual charge and thus lipophilicity. The extent of this ligand-exchange depends on the chloride ion affinity of the complex, expressed as log*K*′(H_2_O/Cl^–^) equilibrium constant, and on the actual chloride ion concentration in the solution. This ligand-exchange process of complexes **1a**–**6a** was investigated by UV–vis and ^1^H NMR spectroscopy, and pH = 6.0 was chosen for the measurements to prevent hydrolytic processes. In the case of RhCp* complexes, the equilibrium could be reached within some min; therefore, the determination of the log*K*′(H_2_O/Cl^–^) constants (Table 1) was performed via titrations with KCl solution, as illustrated by the representative spectra shown for complex **6a** in Figure 7.

**Table 1:** Log*K*’ (H_2_O/Cl^–^) Constants of the Half-Sandwich RhCp* and RuCym Complexes, Determined by UV–vis Titrations and ^1^H NMR Spectroscopic Measurements, Respectively^a^ ^d^

	log*K*’ (H_2_O/Cl^–^)
[RuCym(IQPMA)Cl]Cl (1a)	n.d^a^
[RhCp*(IQPMA)Cl]Cl (3a)	2.87(5)^b^
[RuCym(DIPMA)Cl]Cl (4a)	2.72(1)^c^
[RhCp*(DIPMA)Cl]Cl (6a)	3.16(3)^b^

a Based on the ^1^H NMR spectra, the change in aqua complex fraction was small, even after 4 days (∼10% → ∼14%), making it difficult to determine the constant reliably.

b UV–vis spectra were recorded after a 5 min equilibration time.

c ^1^H NMR spectra were recorded after a 7 day equilibration period.

d {*I* = 0.2 M KNO_3_; *T* = 25.0 °C}.

**Figure 7:**
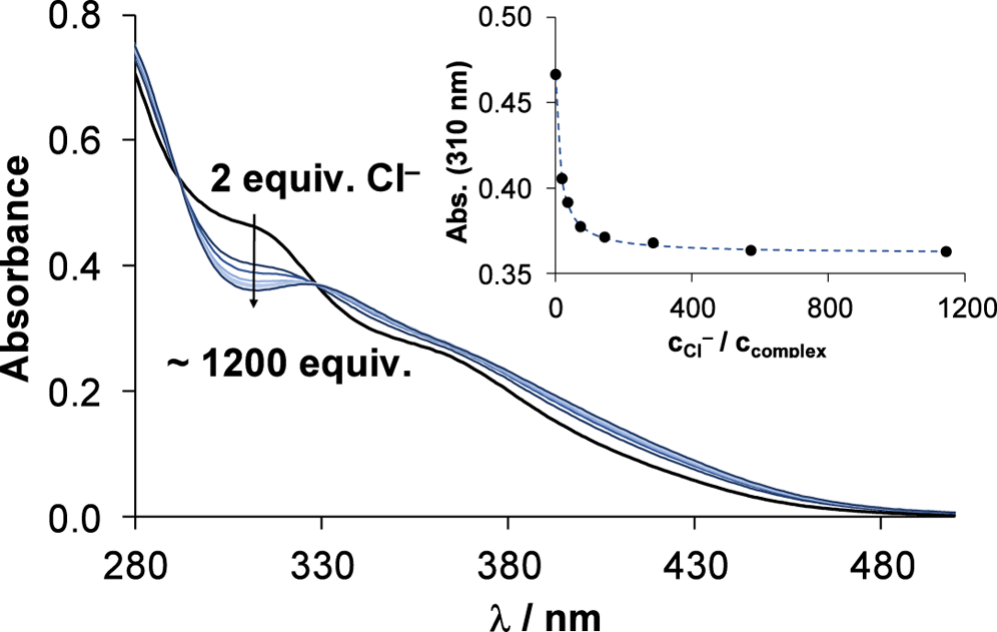
UV–vis spectra recorded for complex **6a** at various equivalents of chloride ions. The inserted figure shows the absorbance values at 310 nm (*) as a function of *c*_Cl_^–^/*c*_complex_ with the fitted (blue dashed) line. {*c*_complex_ = 56 μM, *I* = 0.2 M KNO_3_, l = 1 cm, *T* = 25.0 °C}.

For the RuCym and OsCym complexes, the Cl^–^ → H_2_O exchange reaction was found to be fairly slow. As illustrated by the representative ^1^H NMR spectra shown for **4a** in Figure S56, after the dissolution of the complex, two well-separated sets of signals were observed due to the slow aquation process. Over time, the peaks corresponding to the chlorido species gradually converted into those of the aqua complex, reaching equilibrium after 3 days. Furthermore, adding KCl to the samples shifted the equilibrium toward the chlorido species formation, thereby confirming the assignment of the two peak sets. Based on the integrals of the peaks, the log*K*′(H_2_O/Cl^–^) constants were calculated; however, for complex **1a,** the changes in the aqua complex fraction were too small (Table 1). For the OsCym complexes, the release of the chlorido ligand was even slower, e.g., after 7 days, the equilibrium was not reached (no equilibrium constants were determined, only estimated log*K*′(H_2_O/Cl^–^) ≤ 2.9 for both **2a**, **5a**). In comparison with other analogous complexes, these relatively high exchange constants fall within the expected range,^29,37,40^ exhibiting the somewhat lower chloride affinity of the RuCym complexes compared to the RhCp* derivatives.

The lipophilicity of the ligands IQPMA and DIPMA, as well as their half-sandwich complexes (**1a**–**6a**), was characterized using the traditional shake-flask method with *n*-octanol/water partitioning at pH = 7.4. The phase separation was performed after 4 h (or 2 h for RhCp* complexes) of partitioning. To mimic the physiological environment of biofluids, different chloride concentrations were applied, corresponding to those found in blood (100 mM), cytosol (24 mM), and the nucleus (4 mM).^41^ The presence of chloride ions can affect the lipophilicity of the organometallic half-sandwich complexes, since the coordination of this anion results in a positively charged (+1) complex, while the aqua complex has a +2 charge, thus the latter is more hydrophilic. However, this is relevant only in the case of the RhCp* complexes, where the release of the chlorido coligand is significantly faster. The RuCym and OsCym complexes predominantly remain in their chlorido form after dissolution in aqueous media for long days, as discussed above. The results are shown in Figure 8 (and in Table S7).

**Figure 8:**
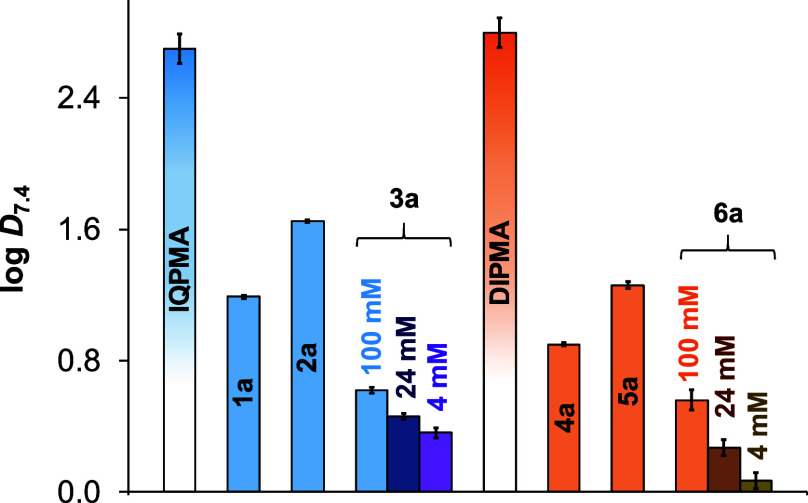
Lipophilicity of the studied compounds (IQPMA, DIPMA, and complexes **1a**–**6a**) expressed as logD_7.4_ using n-octanol/buffered aqueous solution partitioning for 4 h (or 2 h for RhCp* complexes) at pH = 7.4 (20 mM phosphate buffer). The logD_7.4_ values for **3a** and **6a** were determined at different chloride ion concentrations (100, 24, and 4 mM KCl), whereas those for the ligands and complexes **1a**, **2a**, **4a**, and **5a** were determined at 100 mM KCl {*T* = 25.0 °C}.

The ligands exhibit a strong lipophilic character (log*D*_7.4_ = +2.7 ± 0.1 (IQPMA), +2.8 ± 0.1 (DIPMA)). Upon complexation, the hydrophobicity significantly decreased, although the complexes still retain a certain degree of lipophilic character (log*D*_7.4_ > 0). Among the complexes, the OsCym derivatives (**2a**, **5a**) are the most lipophilic, while the RhCp* derivatives (**3a**, **6a**) are the most hydrophilic. Additionally, complexes containing IQPMA are more lipophilic compared to their DIPMA counterparts, as expected. As clearly seen, the lower the chloride ion concentration, the higher the hydrophilic character of the RhCp* complexes.

The thermodynamic solubility of IQPMA and its OsCym complex (**2a**) at pH 7.4 was determined to be *S*_7.4_ = 2.6 ± 0.8 μM and 220 ± 20 μM, respectively, using 24 h for the saturation. The more hydrophilic RuCym (**1a**) derivatives exhibited much higher solubility (*S*_7.4_ > 6 mM). (**3a** was not tested due to its instability at pH 7.4.) These findings clearly demonstrate the beneficial effect of complexation on aqueous solubility.

#### Interaction of Selected Organometallic Complexes with Human Serum Albumin

Binding to blood serum proteins fundamentally influences the path of a drug or drug candidate within the body. Human serum albumin (HSA) is the most abundant protein component of blood serum; therefore, we have tested the binding of the RuCym and OsCym complexes **1a**, **2a**, **4a,** and **5a** to this protein by spectrofluorometry. The RhCp* complexes were not involved due to their lower aqueous stability (at pH = 7.4). Tryptophan (Trp-214) quenching experiments were carried out; the decrease of fluorescence upon addition of a small molecule refers to binding on HSA. The binding of the investigated metal complexes was slow and took longer than 24 h (Figure 9a). From a physiological point of view, there is no point in waiting any longer; therefore, batch samples were made and measured after 24 h. DIPMA complexes did not quench considerably the fluorescence of HSA at 20–30-fold excess, while IQPMA complexes, especially **5a**, quenched the protein’s fluorescence effectively (Figure 9b). Quenching constants were calculated with the computer program HypSpec:^42^ log*K*_Q_’ = 5.4 ± 0.1 (**5a**), 4.9 ± 0.1 (**4a**), 3.9 ± 0.1 (**2a**), <3.8 ± 0.1 (**1a**). Most probably, intermolecular binding of the complexes takes place, since the UV–vis absorption spectra of the complexes were unaltered in the presence of HSA (not shown). The intermolecular binding mode is further supported by the high stability of the metal—(*N,N*) ligand coordination bonds and the strongly inert nature of the chlorido coligand.

**Figure 9:**
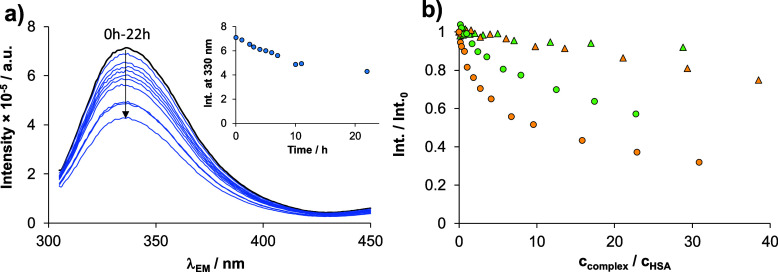
(a) Fluorescence emission spectra of HSA in the presence of 10 equiv. **5a** followed in time; the inserted figure shows the intensities at λ_EM_ = 330 nm as a function of time. (b) Quenching of Trp-214 fluorescence in HSA at λ_EM_ = 330 nm upon addition of **1a** (▲, green), **2a** (▲, yellow), **4a** (●, green), or **5a** (●, yellow) after 24 h waiting {*c*_HSA_ = 1 μM; λ_EX_ = 295 nm; PBS’; *T* = 25 °C}.

#### Evaluation of the In Vitro Cytotoxic Activity of IQPMA, DIPMA, and Their Complexes (1a—6a)

The in vitro cytotoxicity of compounds IQPMA and DIPMA and their organometallic complexes (**1a**–**6a**) was studied in different human cancer cell lines. For the colorimetric 3-(4,5-dimethylthiazol-2-yl)-2,5-diphenyl-tetrazolium bromide (MTT) assay, the chemosensitive Colo205 and the doxorubicin-resistant Colo320 human colon adenocarcinoma cell lines, along with the lung carcinoma A549 and breast adenocarcinoma MCF-7 cancer cell lines, were selected in addition to the noncancerous CCD-19Lu fibroblast cell line. The determined IC_50_ values in the different cell lines are shown in Table 2.

**Table 2:** In Vitro Cytotoxicity of the Two Ligands (IQPMA and DIPMA) and the Six Complexes (**1a**–**6a**) Expressed in IC_50_ (μM) Values, Tested in Human Cancer Cell Lines (Colo205, Colo320, MCF-7, and A549) and in a Non-cancerous Fibroblast CCD-19Lu Cell Line Using 72 h Incubation time. Doxorubicin Was Used as a Positive Control^a^

	Colo205	Colo320	A549	MCF-7	CCD-19Lu
**IC**_**50**_ **/μM**					
**IQPMA**	19 ± 1	21 ± 2	43 ± 3	15 ± 1	11 ± 1
**1a**	28 ± 2	>100	>100	9 ± 1	7 ± 2
**2a**	45 ± 3	93 ± 2	25.9 ± 0.8	38 ± 5	31.4 ± 0.3
**3a**	21 ± 2	21.4 ± 0.1	9.4 ± 0.2	12 ± 1	9 ± 1
**DIPMA**	>100	>100	>100	>100	>100
**4a**	2.14 ± 0.07	>100	38 ± 2	8.1 ± 0.5	20 ± 2
**5a**	3.9 ± 0.4	53 ± 1	6 ± 1	6 ± 1	30 ± 2
**6a**	22 ± 1	>100	>100	31 ± 2	53 ± 2
**doxorubicin**	0.76 ± 0.02	1.01 ± 0.04	0.23 ± 0.01	0.46 ± 0.01	0.20 ± 0.02

a The dinuclear precursors were non-toxic against the tested cell lines (IC_50_ > 100 μM).

Upon analysis of the cytotoxicity data, it can be concluded that IQPMA exhibits moderate cytotoxicity (IC_50_ = 15 – 43 μM). It should be noted that previously reported related indolo­[2,3-*c*]­quinoline derivatives showed lower IC_50_ values on various cancer cell lines compared to IQPMA.^13,19^ For example, trimethylsilyl-substituted indolo­[2,3-*c*]­quinoline Schiff bases showed IC_50_ values of 3.23 μM (A549) and 1.43 μM (MCF-7),^13^ while morpholine-substituted derivatives exhibited IC_50_ values in the range of 0.27–8.3 μM depending on the cell line.^19^ In general, the complexation of IQPMA with the organometallic fragments did not result in a substantial improvement in activity against the Colo205 and Colo320 cell lines, as similar or even higher IC_50_ values were obtained for the corresponding complexes (**1a**–**3a**). Among these complexes, the RhCp* derivative (**3a**) was the most potent across most cell lines, while the RuCym complex (**1a**) displayed an enhanced and selective effect toward MCF-7 cells (IC_50_ = 9.1 μM). In contrast, the OsCym derivative (**2a**) was the least active. The RuCym and OsCym complexes of the analogous indolo­[3,2-*c*]-quinoline were also tested on A549 cells, and IC_50_ > 80 μM were found, while they were quite active on the strongly drug-sensitive CH1 ovarian cancer cells.^23^ On the contrary, the complexation of the above-mentioned related indolo­[2,3-*c*]­quinoline derivatives with Cu­(II) and Zn­(II) typically resulted in higher cytotoxic activity (low micromolar to submicromolar IC_50_ values).^13,19^ The uncoordinated DIPMA exhibits negligible anticancer activity against most cell lines (IC_50_ > 100 μM). The higher cytotoxicity of IQPMA compared to DIPMA may be attributed to the presence of the indolo­[2,3-*c*]­quinoline scaffold, which is known for its intrinsic bioactivity,^13,19^ as well as its increased lipophilicity that may facilitate cellular uptake. Notably, DIPMA complexes (**4a**–**6a**) show higher cytotoxic activity than the free ligand, with IC_50_ values ranging from 2 to 38 μM across the tested cell lines, indicating moderate-to-strong potency, except for the RhCp* complex (**6a**) in Colo320 and A549 and the RuCym (**4a**) complex in Colo320 cells (IC_50_ > 100 μM). Notably, complex **6b** was tested among others against A549 cells by Hu et al. and showed moderate activity (IC_50_ = 30.3 μM),^27^ while the analogous complex [RuCym­(DIPMA)­Cl]­(CH_3_C_6_H_4_SO_3_) displayed IC_50_ = 71.8 μM on the same cell line in another study.^32^ Overall, **6a** is less potent than its RuCym (**4a**) and OsCym (**5a**) counterparts, while for its iridium analogue [IrCp*­(DIPMA)­Cl]­PF_6_, IC_50_ = 28.8 and 13.9 μM values were reported in A549 and HeLa cancer cells using 24 h exposure.^33^ Additionally, the DIPMA complexes of RuCym (**4a**) and OsCym (**5a**) were marginally more cytotoxic in most cell lines than their IQPMA **1a** and **2a** counterparts, while the [RhCp*­(IQPMA)­Cl]Cl (**3a**) complex was slightly more potent than its DIPMA analogue (**6a**). For most compounds, the IC_50_ value is similar or much higher in the doxorubicin-resistant Colo320 cells than in the chemosensitive Colo205 cells, indicating reduced susceptibility of the resistant cell line.

Notably, no general trend in cytotoxic activity can be established, as the observed effects depend strongly on both the metal center and the ligand scaffold. Further studies aimed at elucidating the mechanism of action of these complexes, including apoptosis induction and ROS generation, are planned for future work.

To assess the selectivity of the compounds, IC_50_ values were also determined in the noncancerous CCD-19Lu fibroblast cell line (Table 2). As can be seen, DIPMA exhibited no significant cytotoxic effect in this cell line, while IQPMA ligand showed the lowest IC_50_ value among all tested cell lines, indicating a lack of selectivity. Selectivity ratios [SR = IC_50_ (CCD-19Lu)/IC_50_ (cancer cell line)] were calculated for the organometallic complexes (**1a**–**6a**) (Figure 10). These ratios revealed a notable selectivity of the complexes containing DIPMA, particularly for the RuCym (**4a**) and OsCym (**5a**) complexes, with selectivity ratios ranging from 2.5 to 9.4. In contrast, the values for IQPMA complexes (**1a**–**3a**) were mostly below one, suggesting limited or no selectivity toward cancer cells.

**Figure 10:**
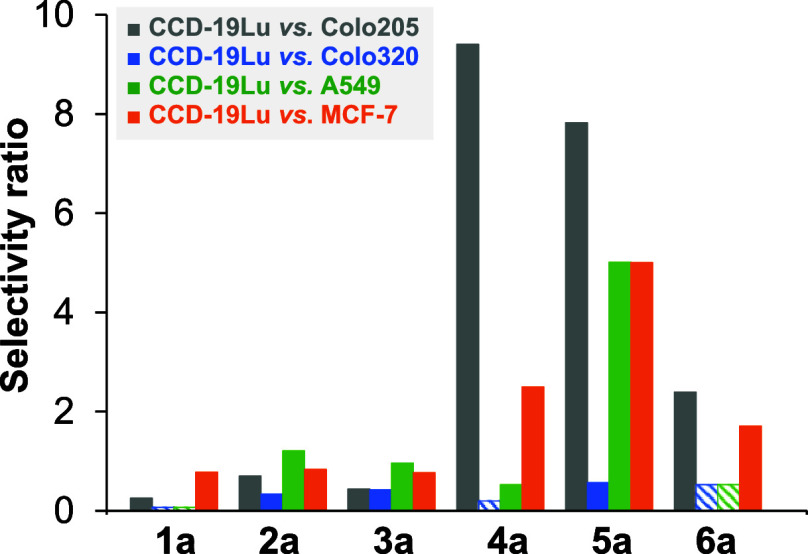
Selectivity ratios for the complexes (**1a**–**6a**) obtained as the quotients of IC_50_ (CCD-19Lu)/IC_50_ (cancer cell line). Striped columns indicate upper limits of SR values. The IC_50_ values are shown in Table 3.

Considering the cytotoxicity and stability results, for further nanoformulation studies, the RuCym complexes (**1a** and **4a**) were selected and tested on MCF-7 cells (vide infra). These derivatives exhibited the most favorable balance between potency and selectivity, allowing a meaningful comparison of the two ligand systems with an identical metal center. The comparison of IQPMA and DIPMA demonstrates how conjugation with the indoloquinoline core can significantly change the biological response.^39^

#### Preparation and Evaluation of Lipid- and Polymer-Based Formulations of 1a and 4a

Polymer-based and lipid-based nanocarriers are often used as drug-delivery platforms for chemotherapeutics, including metal-based compounds.^43^ At first, polymer-based nanocarriers with PLGA—Pluronic (PLUR) matrices were developed and characterized for the encapsulation of complexes **1a** and **4a**, which were selected on the basis of their cytotoxic properties and stability in solution. The PLUR-stabilized PLGA colloidal carriers represent a potentially safe platform for human therapeutic applications.^44^ Two PLGA copolymers differing in their lactic acid/glycolic acid ratios (75:25 and 50:50) were applied; the higher lactic acid content (75:25) yielded a more hydrophobic matrix.^45^ The detailed preparation and characterization of the PLGA-based formulations are provided in the Supporting Information. Despite their good colloidal stability and tunable physicochemical properties, these systems unfortunately showed low drug loading efficiencies (typically <1%) for these organometallic complexes. This very low loading efficiency can be attributed to the predominantly charged and relatively hydrophilic nature of the complexes, which limits their partitioning into the hydrophobic PLGA matrix during nanoprecipitation. Consequently, formulation efforts were redirected toward liposomal systems, expected to provide improved encapsulation and bioavailability.

Asolectin (ASO)-derived liposomes were selected as further carriers, as ASO is a natural, biocompatible, non-immunogenic phospholipid mixture rich in phosphatidylcholine, which supports stable liposomal formation and efficient drug incorporation.^46^ The choice was inspired by the clinical success of lipid-based chemotherapeutics such as liposomal doxorubicin (Doxil), which demonstrated improved pharmacokinetics and tumor selectivity.^47^ Accordingly, liposomal encapsulation might enhance the bioavailability and tumor-targeting via the enhanced permeability and retention (EPR) effect.^48^ Liposomal nanoformulation is a well-established strategy also for metallodrugs to improve their solubility and pharmacokinetic properties.^49–51^ Lipid-based nanoformulations of complexes **1a** and **4a** within ASO dispersions were prepared by the in situ encapsulation method, followed by gel filtration to separate free and liposome-encapsulated drug fractions. The preparation parameters were optimized to obtain liposomes with therapeutically favorable size and stability, and the encapsulation efficiency was assessed using corrected encapsulation efficiency (EE%_corr_) (Table 3, calculation details are provided in the Experimental Section). As shown in Table S10, the lipid-based formulations enhanced the transport of the complexes through the gel, consistent with lighter gel coloration and the higher EE%_corr_ values. Notably, under the applied conditions, a portion of the free compound can elute early together with the liposomes in the void volume, while the remainder is retained within the gel matrix. Knowing that gel filtration results in the aforementioned “leakage” of free complex, the conventionally calculated encapsulation efficiency (EE % = ((total drug added – free drug)/total drug added)×100%) does not fully distinguish between encapsulated and freely eluting drug species. Therefore, EE%_corr_ was calculated, taking into account the amount of complex that elutes early, which was determined by gel filtration of the nonformulated complex. EE%_corr_ offers a relevant estimate of the drug fraction that remains associated with vesicles during filtration.

**Table 3:** Loading Parameters of Complexes (Non-corrected Encapsulation Efficacy Percentage (EE %), the Transported Compound in Free form Percentage (*T*_free_ %), and EE%_corr_ Values of **1a** and **4a** at Different Initial Concentrations in the Lipid-Based Nanocarriers^a^

complex	*c*_initial_ (μM)	*T*_free_ %	EE %	EE%_corr_ ± SD	estimated ***c***_encapsulated_ (μM)
**1a**	100	0.3	72.5	72.4 ± 2.1	72.4
	300	1.9	66.8	66.2 ± 2.4	199.0
	600	7.5	24.7	18.6 ± 3.0	111.7
**4a**	100	11.8	77.0	74.0 ± 2.3	74.0
	300	18.4	71.4	65.0 ± 2.6	195.0
	600	27.7	39.5	16.4 ± 3.1	98.2

a {*c*_ASO_ = 1 mg/mL}.

For both complexes **1a** and **4a** (Table 3), the EE%_corr_ decreased progressively with increasing initial drug concentration, suggesting that lipid-phase saturation limits the ability of liposomes to encapsulate additional drug molecules at higher loadings. It should be noted that the two tested half-sandwich complexes showed similar EE%_corr_ values. For the cytotoxicity assays, the nanoformulations prepared at an initial complex concentration of 100 μM were selected (vide infra), as they exhibited the highest EE%_corr_ values. Physico-chemical characterization of these formulations revealed that the **1a** liposomes exhibited a hydrodynamic diameter (*d*_H_) of 202 ± 6 nm, a zeta potential (ζ) of −65.7 ± 1.6 mV, and a polydispersity index (PDI) of 0.248 ± 0.032. The corresponding values for **4a** liposomes were *d*_H_ = 196 ± 5 nm, ζ = – 68.7 ± 1.5 mV, and PDI = 0.301 ± 0.028. The particle sizes, centered around 200 nm, fall within the optimal range for passive tumor accumulation via the EPR effect, suggesting favorable in vivo targeting potential. Furthermore, the strongly negative zeta potentials indicate excellent electrostatic stability, while the relatively low PDI values confirm the homogeneity and colloidal stability of the liposomal dispersions.

In conclusion, the ASO-based liposomal systems demonstrated high encapsulation efficiency and improved formulation performance compared with the PLGA-based formulation. The introduction of the EE%_corr_ parameter provides a more accurate and functionally relevant estimation of the encapsulation.

#### Evaluation of the In Vitro Cytotoxic Activity of Nanoformulated Complexes 1a and 4a

The anticancer activity of the lipid-based nanoformulated organoruthenium complexes **1a** and **4a** (100 μM) was evaluated on MCF-7 cells, and the determined IC_50_ values are shown in Table 4. The empty liposomes exhibited negligible cytotoxicity. The encapsulation of the complexes into the ASO-based liposomes resulted in a minor increase in IC_50_ values, suggesting that the nanoformulation retained the anticancer activity of the free compounds. These findings are encouraging, as the liposomal encapsulation may offer enhanced pharmacokinetic stability and tumor-targeting potential in vivo.

**Table 4:** In Vitro Cytotoxicity of **1 and 4a** and Their Liposomal Formulations Expressed in IC_50_ (μM) Values, Tested in MCF-7 Cell Lines Using 72 h Incubation Time

	IC_50_/μM in MCF-7
[RuCym(IQPMA)Cl]Cl (1a)	13.8 ± 0.7
(1a)-loaded liposomes	14.4 ± 0.9
[RuCym(DIPMA)Cl]Cl (4a)	12.1 ± 0.5
(4a)-loaded liposomes	13.3 ± 0.8
empty liposomes	>100

#### Antibacterial Activity and Inhibition of Biofilm Formation

Indoloquinoline derivatives exhibited notable antimicrobial properties, including activity against resistant bacterial strains and biofilms,^14,15^ which motivated the investigation of the antibacterial activity of the ligands and complexes **1a**–**6a**. The antibacterial activity of the compounds was tested in various bacterial strains, including the Gram-positive *Staphylococcus aureus*, its methicillin-resistant counterpart (MRSA), *Enterococcus faecalis*, and the Gram-negative *Escherichia coli* and *Klebsiella quasipneumoniae*. The determined minimum inhibitory concentration (MIC) values are shown in Table 5.

**Table 5:** Antibacterial Activity of the Two Ligands (IQPMA and DIPMA) and the Six Complexes (**1a**–**6a**) Expressed in MIC (μM) Values Tested in Various Bacterial Strains

Gram-positive				Gram-negative	
	*S. aureus*	*S. aureus* *MRSA*	*E. faecalis*	*E. coli*	*K. quasipneumoniae*
**MIC/μM**					
**IQPMA**	100	>100	>100	>100	>100
**(1a)**	6.25	50	>100	12.5	>100
**(2a)**	3.12	50	50	25	>100
**(3a)**	50	>100	>100	100	>100
**DIPMA**	>100	>100	>100	>100	>100
**(4a)**	0.05	12.5	12.5	25	>100
**(5a)**	0.098	6.25	12.5	50	>100
**(6a)**	100	>100	>100	>100	>100

The two ligands exhibited no or minimal activity, as their MIC values consistently exceeded 100 μM in nearly all cases. The RhCp* complexes (**3a**, **6a**) were also largely inactive, although they demonstrated a weak antibacterial effect against *S. aureus* (MIC = 50 – 100 μM). In contrast, the RuCym and OsCym complexes (particularly with the DIPMA ligand) showed considerable antimicrobial activity (MIC = 0.05–50 μM), except against *K. quasipneumoniae* (MIC >100 μM), a trend observed for all compounds tested. The RuCym (**1a**) and OsCym (**2a**) complexes also displayed moderate activity (MIC = 6.25–50 μM) against methicillin-resistant *S. aureus* (MRSA), a clinically important multidrug-resistant pathogen.^52^ Overall, significantly higher MIC values were obtained for Gram-negative strains compared to their Gram-positive ones, reflecting the additional permeability barrier of the outer membrane. Coordination to half-sandwich RuCym and OsCym centers markedly improved the antibacterial efficacy relative to the free ligands.

The RuCym (**1a**) and OsCym (**2a**) complexes of IQPMA were further evaluated for their ability to inhibit biofilm formation and proliferation by *S. aureus*, methicillin-resistant *S. aureus* MRSA, and *E. coli* strains. Biofilms play a critical role in the persistence of bacterial infections, making them more difficult to eliminate by using conventional antibiotics. Although both complexes exhibited a strong antibacterial effect against *S. aureus*, they did not significantly inhibit biofilm formation in this strain. At the same time, they demonstrated marked inhibitory effects against MRSA biofilms (41% for **1a** and 66% for **2a**) and moderate antibiofilm activity against *E. coli* (29% for **1a** and 15% for **2a**) (see details in Table S11).

## Conclusion

Synthesis of the RuCym, OsCym, and RhCp* half-sandwich complexes of a novel indolo­[2,3-*c*]-quinoline-based compound, IQPMA, and its structurally related ligand DIPMA was reported. The complexes (**1a**–**6a**) of these (*N,N*) donor ligands have the [M­(η^6^-arene/η^5^-arenyl)­(IQPMA/DIPMA)­Cl]Cl general chemical formula and were characterized by NMR, ESI-MS, and elemental analysis methods. Crystal structures of IQPMA and five organometallic complexes were determined using SC-XRD analysis, revealing the expected pseudo-octahedral geometry of the complexes. The solid-phase structures of the complexes **1b**–**4b** and **6b** (possessing PF_6_^–^ counterion) were compared with previously reported crystal structures of organometallic complexes of DIPMA and its model ligand PMA. Correlation analysis of the SC-XRD structural parameters showed that, beyond the expected metal –Cp*/Cym distance differences, the coordination geometry is highly similar across the series, while conformational variations are mainly governed by steric and electronic effects between the aromatic ring systems. In particular, the angle between the two ligand aromatic rings plays a key role in defining the overall geometry, largely independent of secondary crystal-packing interactions.

The ligands IQPMA, DIPMA, and PMA are Schiff bases; therefore, their potential hydrolytic process was also investigated. The compounds were stable in DMSO, and in an aqueous environment at pH 1.0; however, slow and much faster UV–vis spectral changes were observed at neutral pH and under strongly basic conditions over time, respectively. The latter changes were clearly attributed to the hydrolysis of the CH=N bond, while relatively fast *Z*/*E* isomerization occurred at pH 7.4, when the DMSO stock solutions were strongly diluted with aqueous buffer. The RuCym and OsCym complexes of IQPMA and DIPMA were stable at both pH 1.0 and 7.4, whereas their RhCp* analogs unexpectedly appeared to facilitate the ligand hydrolysis. For the RuCym and OsCym complexes of IQPMA and DIPMA, the Cl^–^/H_2_O exchange was found to be slow; the equilibrium can be reached only after several days or even longer. The determined or estimated log*K*’ (H_2_O/Cl^–^) constants indicated a higher affinity for chloride ions for RhCp* complexes than for RuCym and OsCym derivatives. The free IQPMA and DIPMA ligands are strongly lipophilic (log*D*_7.4_ = +2.7 and +2.8, respectively), but complexation considerably reduced their hydrophobicity. The aqueous solubility of the organometallic complexes increased by orders of magnitude compared to the free ligands. RuCym and OsCym complexes of IQPMA show considerable affinity (log*K*_Q_’ = 4.9–5.4) toward the blood carrier HSA; most probably, intermolecular binding takes place.

Following limited success with polymer-based PLGA–Pluronic formulations, asolectin-derived liposomes were utilized for RuCym complexes **1a** and **4a**. These provided efficient encapsulation and excellent colloidal stability. The resulting liposomes were homogeneous nanoparticles (∼200 nm) with strongly negative zeta potentials, which are favorable features for passive tumor accumulation via the EPR effect. Encapsulation efficiency was relatively high, but decreased with increasing initial drug concentration, though it remained similar for both complexes. Overall, asolectin-based liposomal systems proved to be a robust and effective platform for nanoformulating these organometallic complexes.

IQPMA exhibited moderate cytotoxic activity, and its complexes generally did not enhance activity across the tested cell lines. However, the RhCp* complex **3a** was the most effective in this series, and the RuCym complex **1a** exhibited selective activity toward MCF-7 cells. DIPMA was noncytotoxic; however, its complexes (**4a**–**6a**) exhibited improved activity. Cytotoxicity was generally lower in drug-resistant Colo320 cells than in Colo205 cells, indicating reduced susceptibility of the resistant phenotype. Encapsulation of RuCym complexes **1a** and **4a** into asolectin-based liposomes resulted in only a minor increase in the IC_50_ values, indicating that the nanoformulation largely preserved the anticancer activity of the compounds.

## Experimental Section

### Materials

All solvents were of analytical grade and used without further purification. [Rh­(η^5^-C_5_Me_5_)­(μ-Cl)­Cl]_2_, [Ru­(η^6^-*p*-cymene)­(μ-Cl)­Cl]_2_, PMA, DIPMA, *n*-octanol, CD_3_OD, DMSO-*d*_6_, D_2_O, EMEM, HSA (A8763, essentially globulin-free), 4,4-dimethyl-4-silapentane-1-sulfonic acid (DSS), and doxorubicin were Sigma-Aldrich products and were used without further purification. The [Os­(η^6^-*p*-cymene)­(μ-Cl)­Cl]_2_ precursor was prepared as it was reported previously.^53^ NaH_2_PO_4_, Na_2_HPO_4_, KH_2_PO_4_, KCl, KNO_3_, NaCl, HNO_3_, KOH, HCl, DMSO, DMF, toluene, methanol, ethanol, acetone, diethyl-ether, *n*-hexane, CHCl_3_, and CH_2_Cl_2_ were Molar Chemicals or VWR products. Milli-Q water was used for sample preparation. For the synthesis, ligands, reagents, and solvents were purchased from commercial suppliers (Sigma-Aldrich and Alfa Aesar) and were used without further purification.

### Stock Solutions and Sample Preparation

Milli-Q water was utilized to prepare both stock and sample solutions. The stock solutions of the organometallic complexes were made by directly dissolving the isolated complexes in aqueous media. To ensure physiological relevance and maintain stability during the investigation, a modified PBS buffer (PBS’, pH = 7.4) was used. The PBS buffer contains 12 mM Na_2_HPO_4_, 3 mM KH_2_PO_4_, 1.5 mM KCl, and 100.5 mM NaCl, with the concentrations of K^+^, Na^+^, and Cl^–^ ions similar to those found in human blood serum. Stock solutions of HSA were prepared in the PBS buffer, and the concentration was determined by its UV absorption (λ_280nm_ = 36,850 M^–1^ cm^–1^).^54^ For formulation purposes, stock solutions of **1a** and **4a** complexes were prepared in Milli-Q water.

### Synthesis and Characterization of IQPMA and the Half-Sandwich Complexes

IQPMA was synthesized via a multistep route, followed by coordination to the corresponding metal precursors to yield the half-sandwich complexes. All compounds were fully characterized by NMR, ESI-MS, and elemental analysis. The presence of water in the isolated samples was supported by Fourier Transform Infrared spectroscopy spectra (Figure S57). Detailed synthetic procedures and characterization data are provided in the Supporting Information.

### NMR Spectroscopy Applied for the Solution Studies

A Bruker Avance III HD Ascend 500 Plus instrument was used to perform the solution studies in an aqueous environment. For this purpose, the WATERGATE water suppression pulse scheme was used in the presence of 10% (*v/v*) D_2_O or 50% DMSO-*d*_6_ and DSS internal standard (to obtain reference peak). pH-dependent processes of 0.73 mM or 3 mM PMA were studied at *I* = 0.1 M KCl in 10% (*v/v*) D_2_O/water. While the same experiments for DIPMA and IQPMA required 50% DMSO-*d*_6_/water and 0.3 mM ligand concentrations. To study the H_2_O/Cl^–^ exchange process, the sample contained 6.26 mM RuCym or OsCym complex concentration, and the chloride ion concentration was 12.52 mM.

### Crystallization, X-ray Data Collection, Structure Solution, and Refinement

A single crystal of IQPMA (**I**) (orange, plate crystal) was obtained from DMF solution in an NMR tube, and diethyl ether was layered on it. For single crystal growth of complexes **1b**–**4b** and **6b**, the chloride counterion was replaced with a hexafluorophosphate anion to improve crystallization. The complexes containing chloride counterions (**1a**–**4a**, **6a**) were dissolved in DMF, followed by the addition of 1 equiv. NH_4_PF_6_. The mixture was stirred for 48 h. Afterward, two different downstream processes were applied. For the complexes containing the IQPMA ligand (**1b**–**3b**), the solvent was partly evaporated, followed by the addition of diethyl ether. The mixture was vacuum-filtrated, and the product (complex with PF_6_^–^ counterion) was washed with water to remove NH_4_Cl. For the complexes containing the DIPMA ligand (**4b** and **6b**), the mixture was vacuum-filtrated (to remove NH_4_Cl), and the product (complex with PF_6_^–^ counterion) was obtained by the addition of diethyl ether.

Single crystals of [RuCym­(IQPMA)­Cl]­PF_6_×DMF (+solvent) (**1b**) (orange, block crystal) and [OsCym­(IQPMA)­Cl]­PF_6_×DMF (+solvent) (**2b**) (reddish crystal) were obtained from DMF solution. In the former case, the solution was placed in an NMR tube, and diethyl ether was layered on it. In the latter case, we used the vapor diffusion method with diethyl ether. A single crystal of [RhCp*­(IQPMA)­Cl]­PF_6_ ×DMF (**3b**) (orange, block crystal) was obtained from DMF solution. The solution was layered on H_2_O in an NMR tube. Single crystals of [RuCym­(DIPMA)­Cl]­PF_6_ (**4b**) (orange, chunk crystal) and RhCp*­(DIPMA)­Cl]­PF_6_ (**6b**) (orange, block crystal) were obtained from a CH_2_Cl_2_ solution of the compounds with diethyl ether by the vapor diffusion method.

SC-XRD data processing and absorption correction were performed using standard procedures, and the structures were solved by direct methods and refined with established programs.^55–59^ Graphical representations were prepared using Mercury.^60^ Full details of data collection, refinement (including treatment of disorder and solvent masking), and crystallographic parameters are provided in the Supporting Information (Tables S1 and S2). All crystallographic information files (CIFs) were deposited in the Cambridge Crystallographic Data Center with numbers CCDC 2529816–2529821.

Statistical analysis was performed on the structural data to reveal correlations and similarities between complexes and their parameters. CA was performed on the standardized data set, where samples are grouped on the basis of similarities without taking into account the information about the class membership. This technique is based on the idea that the similarity is inversely related to the distance between samples. CA calculates the distances (or correlation) between all samples using a defined metric, which is Euclidean distance in our case. Grouping of the samples was performed by Ward’s method clustering algorithm. Linear correlations between all data were calculated by Pearson’s values (R). All statistical evaluations were accomplished with the software Statistica 13.1.^36^

### Preparation of the Drug-free Liposomal Carriers

Liposomal carrier particles were prepared by a previously validated protocol reported in our former work.^61^ A cost-effective soybean-derived phospholipid mixture, asolectin (ASO, Sigma), was used as the lipid source. In each case, 2 mg of ASO was dissolved in 2 mL of CHCl_3_/CH_3_OH (9:1, *v*/*v*) solvent mixture, and the solvents were evaporated under reduced pressure (∼5 min, 50 °C, 100 rpm) to form a thin lipid film. The resulting transparent film was hydrated with 2 mL of Milli-Q water under magnetic stirring for 1 h, yielding a 1.0 mg/mL dispersion. During hydration, the phospholipids spontaneously self-assembled into closed bilayer structures, forming drug-free (empty) liposomes. The formation of liposomes by film hydration was first demonstrated by Bangham et al.^62^

### Preparation and Purification of the Drug-Containing Liposomal Nanoformulations

Liposomes loaded with organometallic complexes **1a** and **4a** were prepared using the in situ encapsulation method, in which liposome formation and drug entrapment occur simultaneously. Following the procedure described in the previous section, the lipid film was hydrated with 2 mL of the respective complex stock solution (complex dissolved in Milli-Q water) and stirred for 1 h. Nonencapsulated complexes were removed by the standard method, Sephadex G-type size-exclusion gel filtration,^48^ where liposomes pass through the gel bed more rapidly, while smaller, free molecules are retained within the pores. The gel phase was prepared by swelling 10 g of cross-linked dextran-based gel (Sephadex G-50; Sigma) in 200 mL of Milli-Q water, yielding ∼20 mL of compact gel bed. Then, 500 μL of the gel was transferred into a microcentrifuge filter unit and centrifuged at 13,000 rpm for 5 min to compact the bed. The procedure was repeated twice, discarding the filtrate each time. The drug-loaded liposomal dispersion was applied onto the gel bed, centrifuged at 7000 rpm for 10 min. The actual complex concentration in the collected fraction was determined spectrophotometrically, using an Agilent Cary 3500 spectrophotometer (Figure S57). The same instrument was used for all further UV–vis spectroscopic measurements.

### Determination of Corrected Encapsulation Efficiency

The encapsulation efficiency (EE %) represents the encapsulated fraction of the compound.

1 $$EE\%={\left[compound\right]}_{encapsulated}/c_{compound}\times 100$$

where *c*_compound_ is the sum of the free and encapsulated compound concentrations ([compound]_free_ + [compound]_encapsulated_). Conventional calculations (EE%_non corr._) assume that the encapsulated concentration is identical with the compound’s concentration filtered into the void volume (*c*_filtered_). During the size-exclusion gel filtration of the lipid-based formulations, it was observed that the organometallic complexes were not completely retained within the gel matrix under the conditions used, and a certain fraction passed through the column. Assuming that the fraction of the early eluting noncapsulated compound is constant, this can be taken into account for samples of liposomal formulations by determining this fraction (*T*_free_ or *T*_free_ %) for a sample containing only the metal complex. This way, *c*_filtered_ is calculated as follows

2 $$c_{filtered}={\left[compound\right]}_{free}\times T_{free}+{\left[compound\right]}_{encapsulated}$$

The combination of eqs 1 and 2 allows the calculation of corrected EE % (EE%_corr_)­

3 $$EE{\%}_{corr}=\left(c_{filtered}-c_{compound}\times T_{free}\right)/\left\{\left(1-T_{free}\right)\times c_{compound}\right\}\times 100$$

Namely

4 $$EE{\%}_{corr}=\left(EE{\%}_{non\;corr.}-T_{free}\%\right)/\left(100-T_{free}\%\right)$$

After gel filtration, the eluate was diluted 15-fold to minimize potential light scattering effects from vesicular particles. The drug concentration in the eluted fractions was then determined by UV–vis spectrophotometry. The uncertainty of the calculated EE%_corr_ values was estimated by standard error propagation, using the experimental variances of the transported fractions obtained for both the free and liposomal samples. The resulting standard error typically fell within ±2–3%, corresponding to an uncertainty of ∼1.5–3.5% in the calculated encapsulation ratio. The gel-filtered samples were used for the biological assays within ∼1 h of the filtration step.

### Characterization of the Nanoformulations

For the characterization of the polymer- and lipid-based particles, the average size, size distribution, and zeta potential (ζ-potential) values were measured by a HORIBA SZ-100 NanoParticle Analyzer. For each sample (after 10-fold dilution), measurements were performed in triplicate, and for each sample, 10 parallel data points were recorded. The light source was a semiconductor laser (λ = 532 nm, 10 mW), and photomultiplier tubes were used as detectors at a 90° scattering angle. For the calculation of the zeta potential values, the Smoluchowski equation was used by converting the measured electrophoretic mobility data.

### UV–Visible Spectrophotometry and Spectrofluorometry

UV–vis spectra were recorded within the wavelength range of 190–1100 nm with a path length of 1 cm. The concentrations of the ligands and complexes ranged from 10 to 200 μM. The spectra were corrected for both background and baseline. Equilibrium constants were determined using the HypSpec computer program.^42^

Fluorescence measurements were performed using a Fluoromax (Horiba Jobin Yvon) spectrofluorometer with a 1 × 1 cm quartz cuvette. Fluorometric measurements were conducted to investigate the interactions of the RuCym and OsCym complexes with 1 μM HSA in PBS medium at 25 °C. The quenching constants (*K*_Q_) for the HSA-complex species were calculated using the HypSpec program,^42^ with calculations based on data from at least two independent measurements. The self-absorbance and inner filter effects were considered, and corrections were applied as described in our previous works.^38,63^

### Determination of Aqueous Thermodynamic Solubility at pH = 7.4 (*S*_7.4_)

Thermodynamic solubility (*S*_7.4_) of the compounds was assessed after a 24 h waiting time by measuring the saturation levels in water at pH = 7.4 (PBS buffer) and 25.0 ± 0.1 °C. The concentration of the compounds was determined by UV–vis spectrophotometry. For calibration, stock solutions of the compounds were used with known concentrations dissolved in 100% DMSO, 75% DMSO, and 50% (*v/v*) DMSO/buffered aqueous solutions.

### In Vitro Biological Assays

In vitro biological activity was assessed by MTT-based cytotoxicity assays on human cancer and normal cell lines, with IC_50_ values obtained by sigmoidal fitting using GraphPad Prism,^64^ as well as by antibacterial MIC determination following CLSI guidelines.^65^ Detailed experimental procedures are provided in the Supporting Information.

## Supplementary Material



## Notes

^1^H and ^13^C NMR spectra of the compounds; synthesis and characterization of the novel compounds; methods used for the characterization of the isolated solid compounds; crystallographic and statistical data; time-dependent UV–vis and ^1^H NMR spectra of ligands and complexes at different pH and matrices; pH-dependent UV–vis and ^1^H NMR spectra of PMA; fluorescence excitation and emission spectra of IQPMA; Log*D*_7.4_ values of IQPMA, DIPMA, and their complexes; description of preparation of PLGA-based nanoformulations and its physicochemical characterizations; determination of drug loading; and experimental for the biological assays (PDF)

## Abbreviations

IQPMA: (2-((pyridin-2-ylmethylene)­amino)-5*H*-indolo­[2,3-*c*]­quinolin-6­(7*H*)-one
DIPMA: 2,6-diisopropyl-*N*-(pyridin-2-ylmethylene)­aniline
PMA: *N*-(pyridin-2-ylmethylene)­aniline
RhCp*: Rh­(η^5^-C_5_Me_5_)
RuCym: Ru­(η^6^-*p*-cymene)
OsCym: Os­(η^6^-*p*-cymene)
p*K*_a_: proton dissociation constant
SR: selectivity ratio
ESI-MS: electrospray ionization mass spectrometry
NMR: nuclear magnetic resonance
ORTEP: Oak Ridge Thermal-Ellipsoid Plot Program
UV–vis: UV–visible
EDTA: ethylenediaminetetraacetic acid
EMEM: Eagle’s Minimum Essential Medium
HSA: human serum albumin
MIC: minimum inhibitory concentration
Trp: tryptophan
EE %: encapsulation efficiency
MRSA: methicillin-resistant *S. aureus*
PBS’: phosphate-buffered saline
ATCC: American Type Culture Collection
CLSI: Clinical and Laboratory Standard Institute
PLGA: poly­(lactic-*co*-glycolic acid)
THF: tetrahydrofuran
(SC-XRD): single crystal X-ray diffraction
DMF: dimethylformamide
Cp*: pentamethylcyclopentadienyl anion
Cym: *p*-cymene
DMSO: dimethyl sulfoxide
PLUR: pluronic
ASO: asolectin
Doxil: liposomal doxorubicin
EPR: enhanced permeability and retention effect
EE%_corr_: corrected encapsulation efficiency
*d*_H_: hydrodynamic diameter
PDI: polydispersity index
MTT: 3-(4,5-dimethylthiazol-2-yl)-2,5-diphenyl-tetrazolium bromide
DSS: 4,4-dimethyl-4-silapentane-1-sulfonic acid
CA: cluster analysis
CV: crystal violet
