# Supplementary material for: Organometallic Complexes with an Indolo[2,3‑c]Quinoline-Derived Ligand: From Structural Features and Solution Speciation to Nanoformulation for Enhanced Therapeutic Potential
Source: Inorg Chem. 2026 May 25;65(22):12422–37. doi: 10.1021/acs.inorgchem.6c01228 (PMC13250974; doi:10.1021/acs.inorgchem.6c01228)
Supplement: Supplementary file 1 [file ic6c01228_si_001.pdf]

# Organometallic Complexes with an Indolo[2,3-*c*]quinoline-derived Ligand: From Structural Features and Solution Speciation to Nanoformulation for Enhanced Therapeutic Potential

*Tamás Pivarcsik,[a] Egon F. Várkonyi,[a,b,c] János P. Mészáros,[a] Orsolya Dömötör,[a] Márta Nové,[d] Gabriella Spengler,[d] Nóra V. May,[e] Petra Bombicz,[e] Christopher Wittmann,[f] Felix Bacher,[f] Vladimir B. Arion,[f,g] Edit Csapó,[b,c] Éva A. Enyedy[a,]**

[a] Department of Molecular and Analytical Chemistry, University of Szeged, Dóm tér 7-8, H-6720 Szeged, Hungary

[b] MTA-SZTE Lendület "Momentum" Noble Metal Nanostructures Research Group, University of Szeged, Rerrich B. tér 1, H-6720 Szeged, Hungary

[c] Department of Physical Chemistry and Materials Science, University of Szeged, Rerrich B. tér 1, H-6720 Szeged, Hungary

[d] Department of Medical Microbiology, Albert Szent-Györgyi Health Center and Albert Szent-Györgyi Medical School, University of Szeged, Semmelweis u. 6, H-6725 Szeged, Hungary

[e] Centre for Structural Science, HUN-REN Research Centre for Natural Sciences, Magyar Tudósok krt. 2, H-1117 Budapest, Hungary

[f] Institute of Inorganic Chemistry, Faculty of Chemistry, University of Vienna, Währinger Str. 42, 1090 Vienna, Austria

[g] Department of Inorganic Polymers, "Petru Poni" Institute of Macromolecular Chemistry, Aleea Gr. Ghica Voda 41 A, 700487 Iasi, Romania

# Table of contents


$^{1}H$ and $^{13}C$ NMR spectra ........ SI-2

Synthesis and characterization of IQPMA and its intermediates ........ SI-19

Synthesis and characterization of the half-sandwich complexes ........ SI-20

Methods used for the characterization of the isolated solid compounds ........ SI-20

X-ray data collection, structure solution and refinement ........ SI-25

SC-XRD data ........ SI-25

Correlation tables and figures for SC-XRD data ........ SI-40

Stability of the ligands: fluorescence, UV-vis and $^{1}H$ NMR spectra ........ SI-44

Stability of the complexes: UV-vis and $^{1}H$ NMR spectra ........ SI-50

Log$D_{7.4}$ values of the complexes and their determination method ........ SI-58

Description of preparation of PLGA-based nanoformulations, physico-chemical characterizations and determination of drug loading ........ SI-59

Data for biofilm inhibition ........ SI-64

IR spectra of complexes **1a**–**4a** ........ SI-65

Experimental for the biological assays ........ SI-66

References ........ SI-69

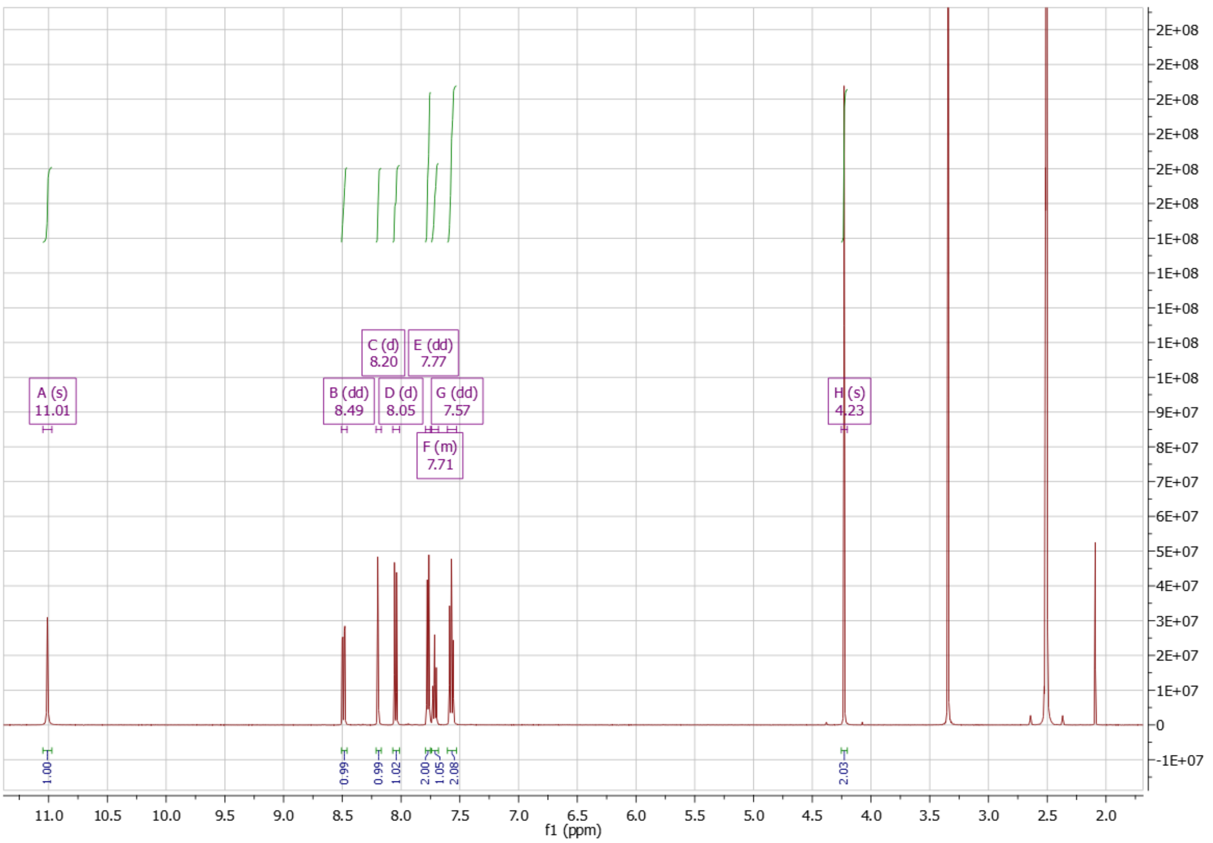


**Figure S1.** $^1$H NMR spectrum of species **B** in DMSO-$d_6$.

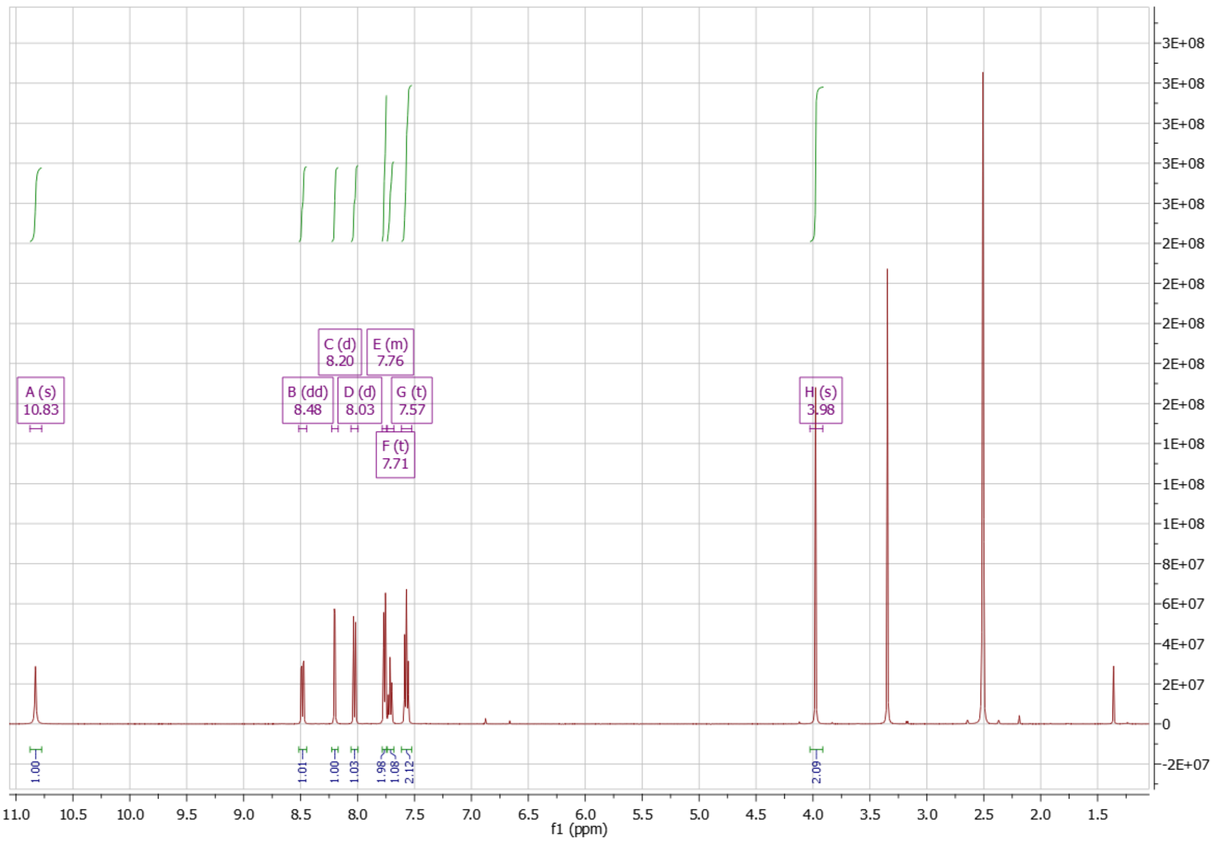


**Figure S2**. $^1$H NMR spectrum of species **C** in DMSO-$d_6$.

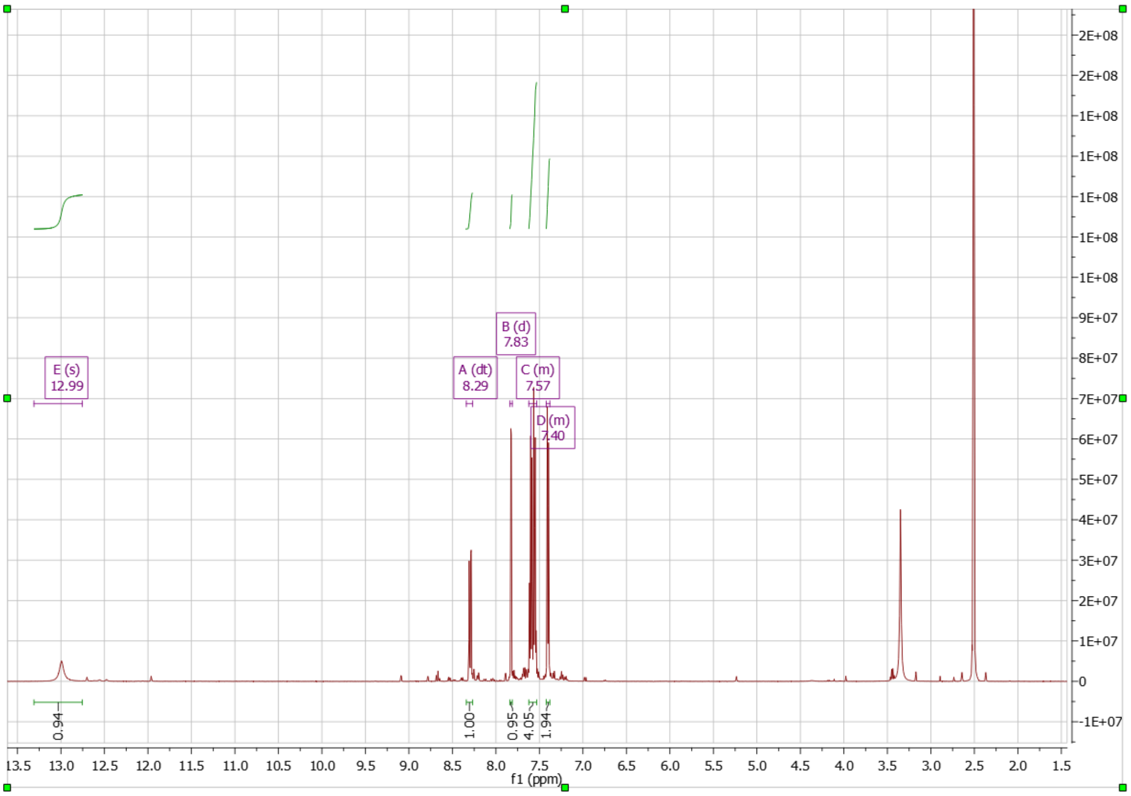


**Figure S3.** $^{1}$H NMR spectrum of **D** in DMSO-$d_6$. Minor additional low-intensity signals are present in the spectrum of this intermediate, which was used in the next reaction step without exhaustive purification.

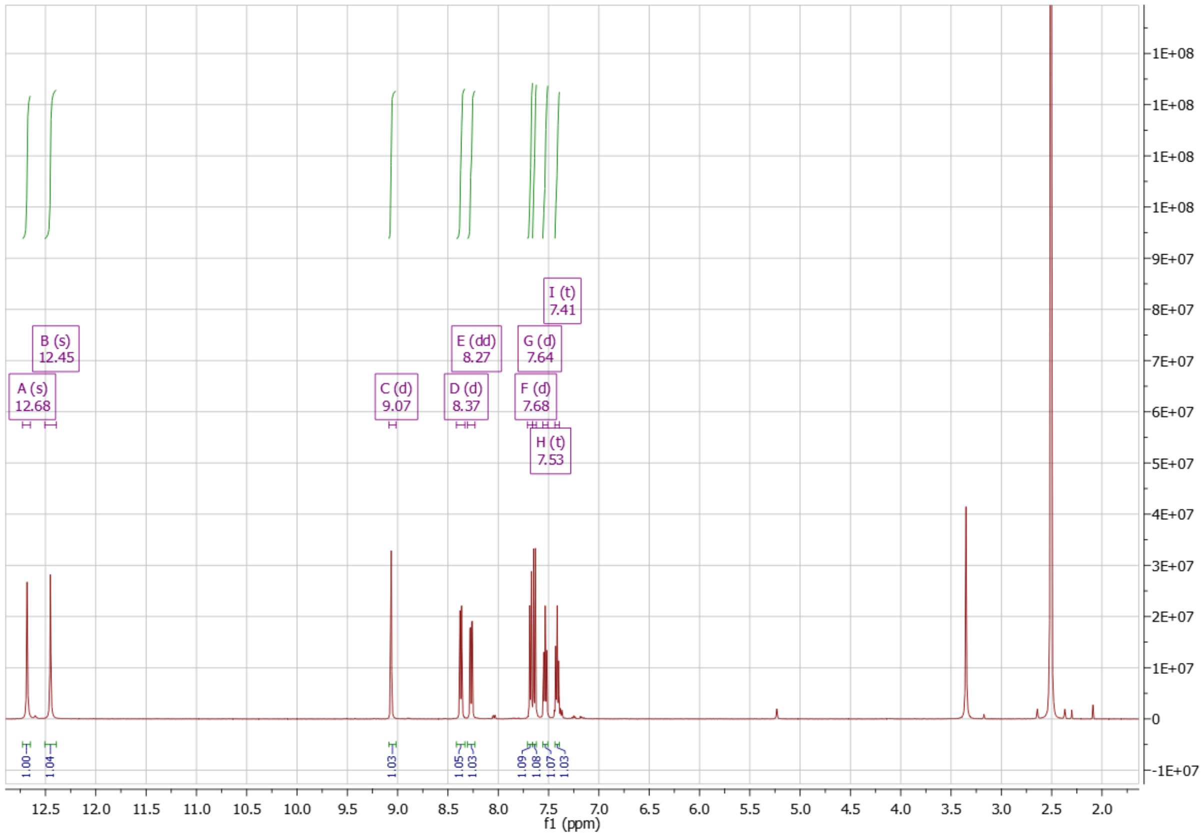


**Figure S4.** $^{1}$H NMR spectrum of **E** in DMSO-$d_6$.

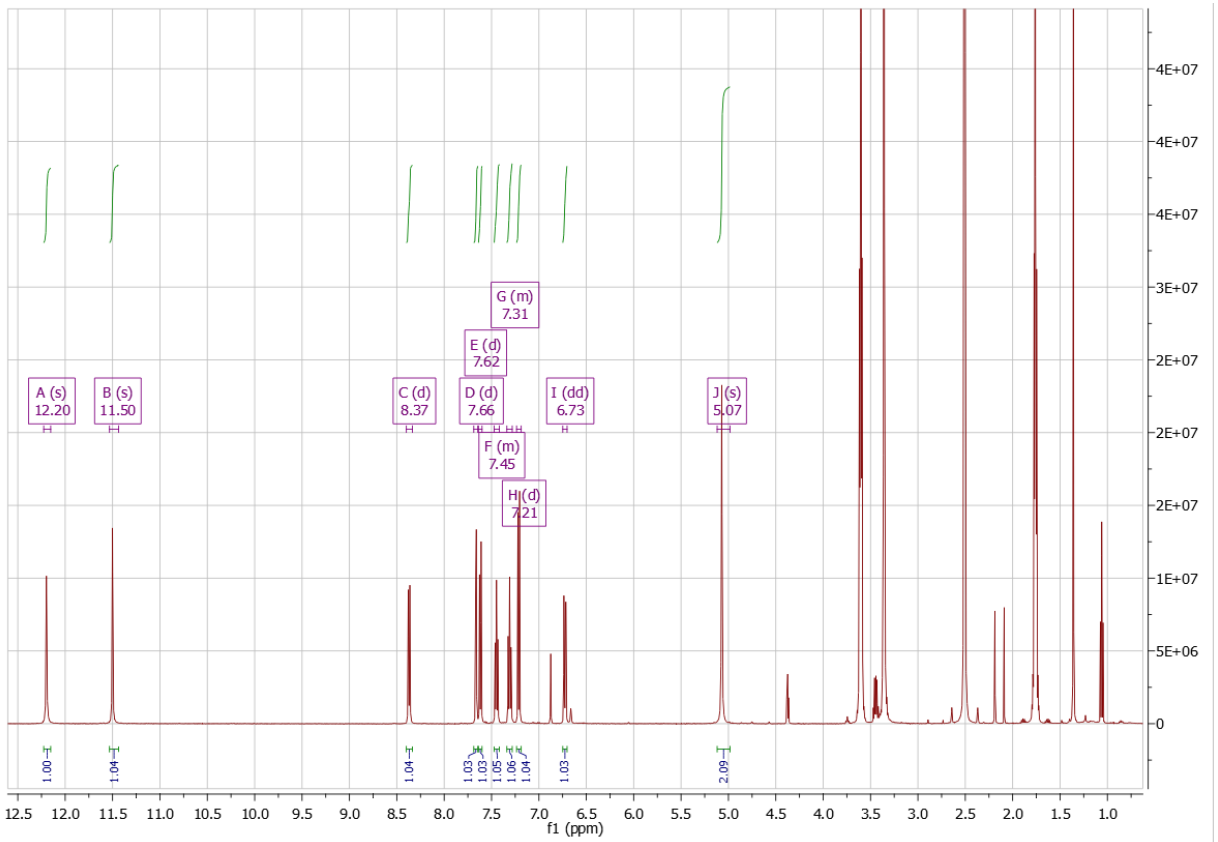


**Figure S5.** $^{1}$H NMR spectrum of **F** in DMSO-$d_6$. Residual THF and hexane proton signals are also seen.

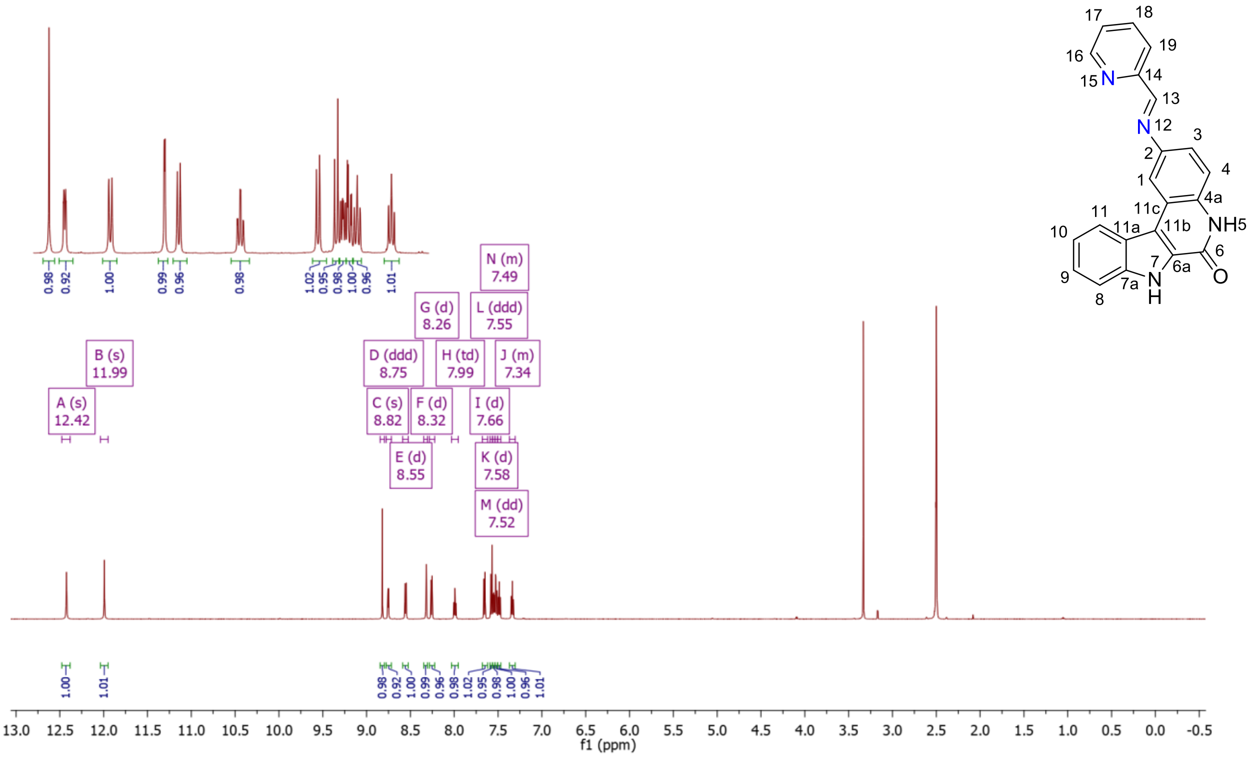


**Figure S6.** $^{1}$H NMR spectrum of IQPMA in DMSO-$d_6$. Inserted structure shows the numbering of peaks.

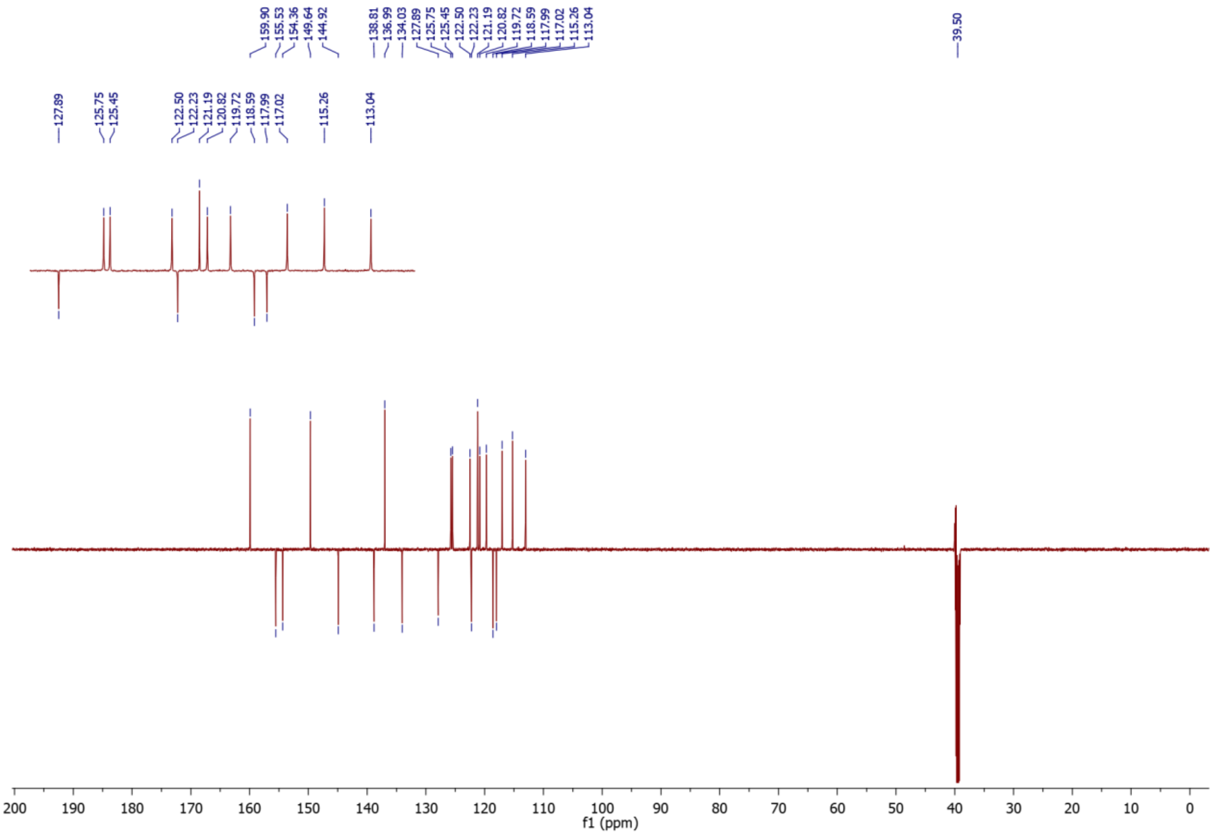


**Figure S7.** $^{13}C$ APT NMR spectrum of IQPMA in DMSO-$d_6$. Attached proton test method: CH and $CH_3$ peaks are positive, C and $CH_2$ peaks are negative.

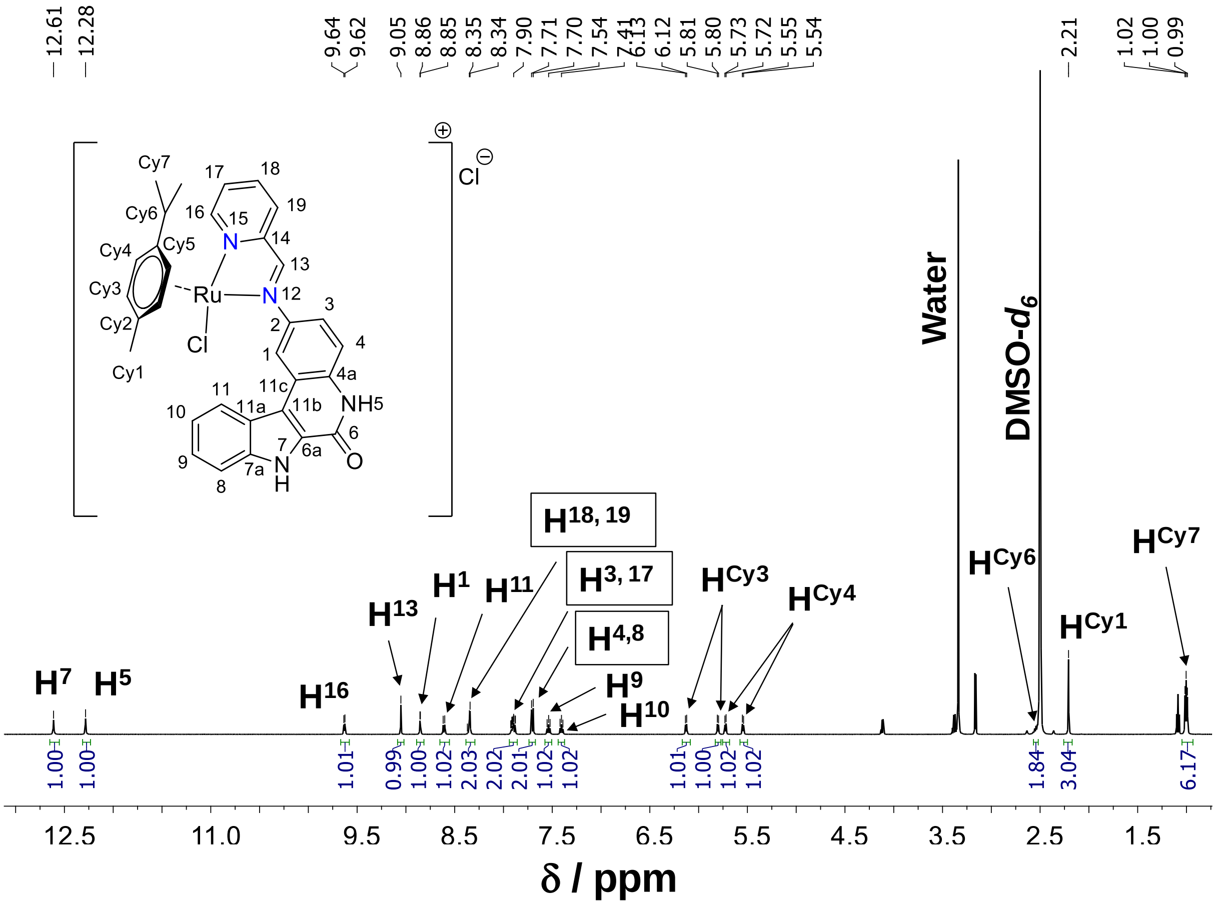


**Figure S8.** $^1$H NMR spectrum of [RuCym(IQPMA)Cl]Cl (**1a**) in DMSO-$d_6$. Inserted structure shows the numbering of peaks. (Peaks highlighted with rectangle are overlapping.)

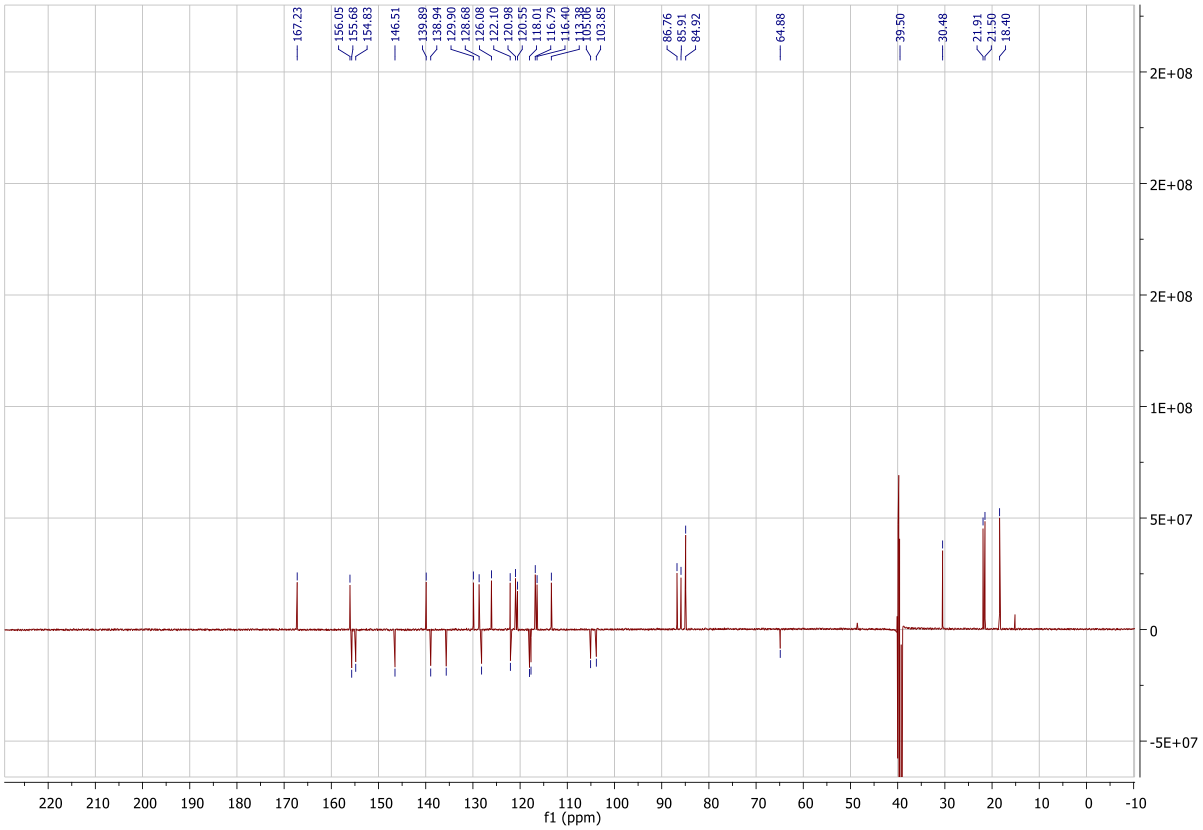


**Figure S9.** $^{13}C$ APT NMR spectrum of [RuCym(IQPMA)Cl]Cl (**1a**) in DMSO-$d_6$. Attached proton test method: CH and $CH_3$ peaks are positive, C and $CH_2$ peaks are negative.

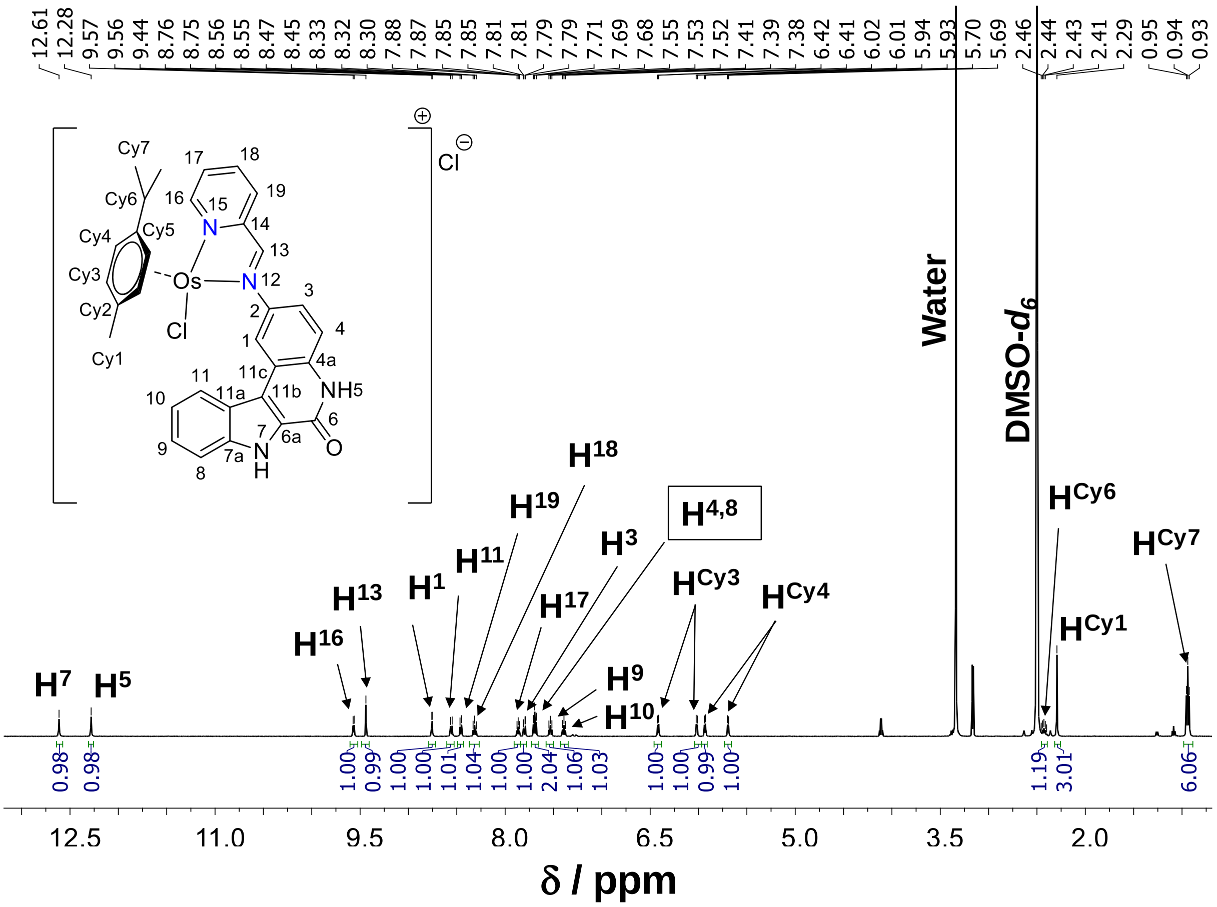


**Figure S10.** $^{1}H$ NMR spectrum of [OsCym(IQPMA)Cl]Cl (**2a**) in DMSO-$d_6$. Inserted structure shows the numbering of peaks. (Peaks highlighted with rectangle are overlapping.)

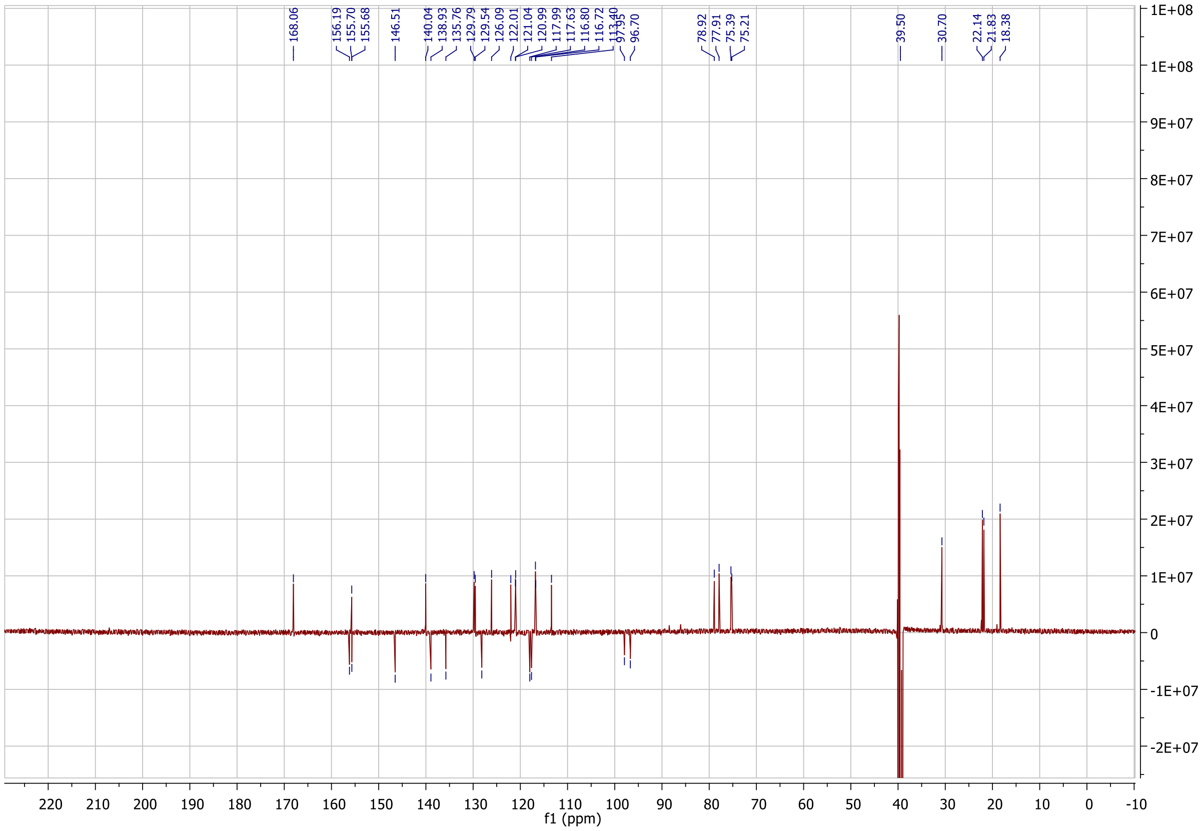


**Figure S11.** $^{13}C$ APT NMR spectrum of [OsCym(IQPMA)Cl]Cl (**2a**) in DMSO-$d_6$. Attached proton test method: CH and $CH_3$ peaks are positive, C and $CH_2$ peaks are negative.

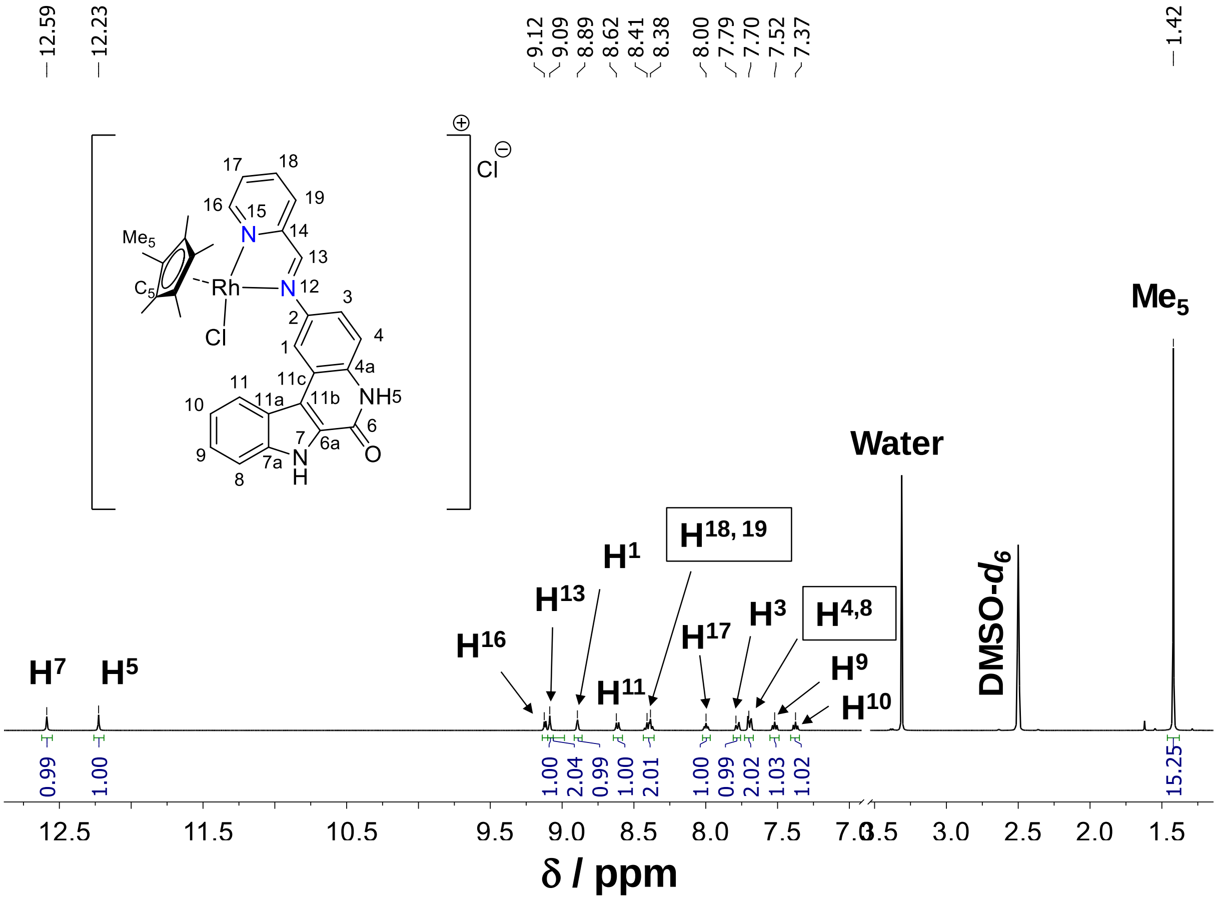


**Figure S12.** $^{1}H$ NMR spectrum of [RhCp*(IQPMA)Cl]Cl (**3a**) in DMSO-$d_6$. Inserted structure shows the numbering of peaks. (Peaks highlighted with rectangle are overlapping.)

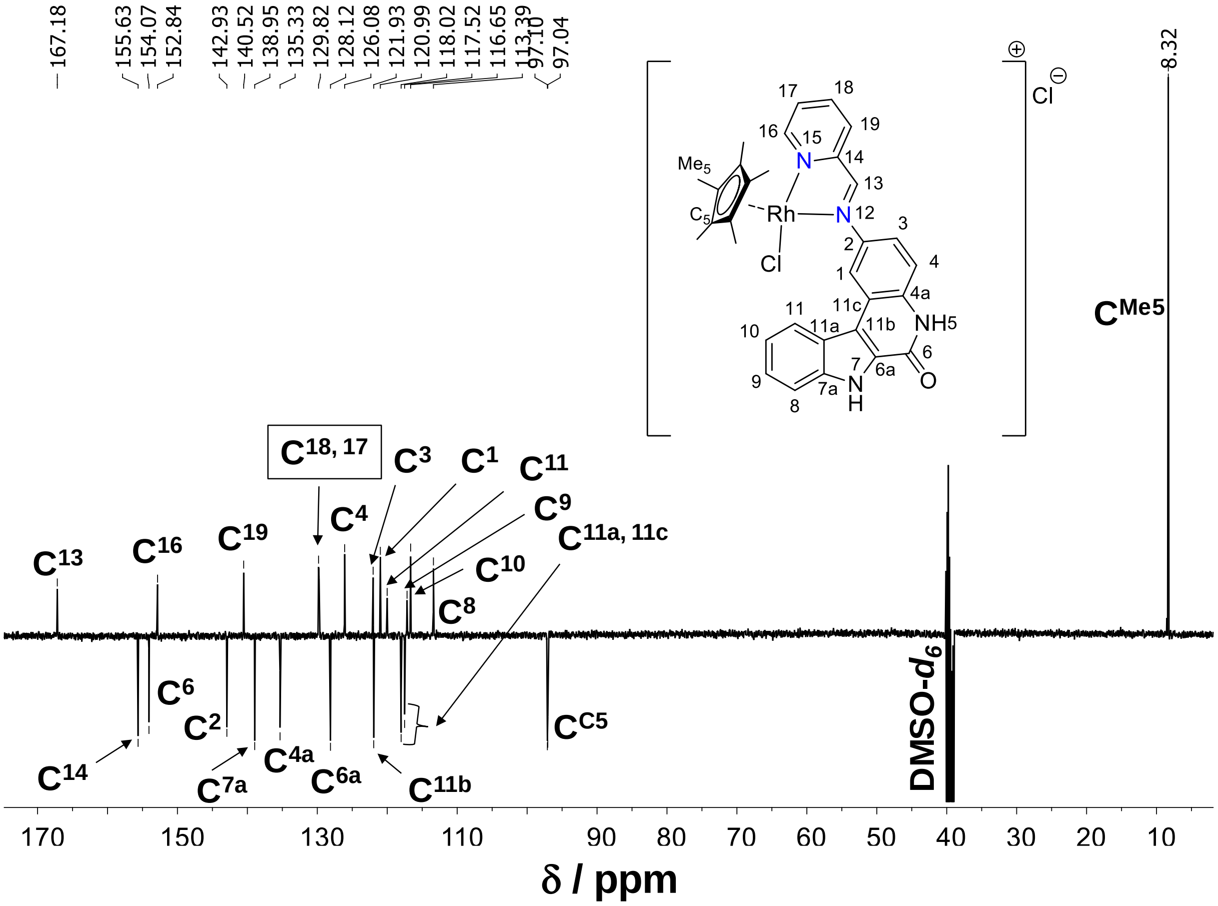


**Figure S13.** $^{13}C$ APT NMR spectrum of [RhCp*(IQPMA)Cl]Cl (**3a**) in $CD_3OD$. Attached proton test method: CH and $CH_3$ peaks are positive, C and $CH_2$ peaks are negative. (Peaks highlighted with rectangle are overlapping.)

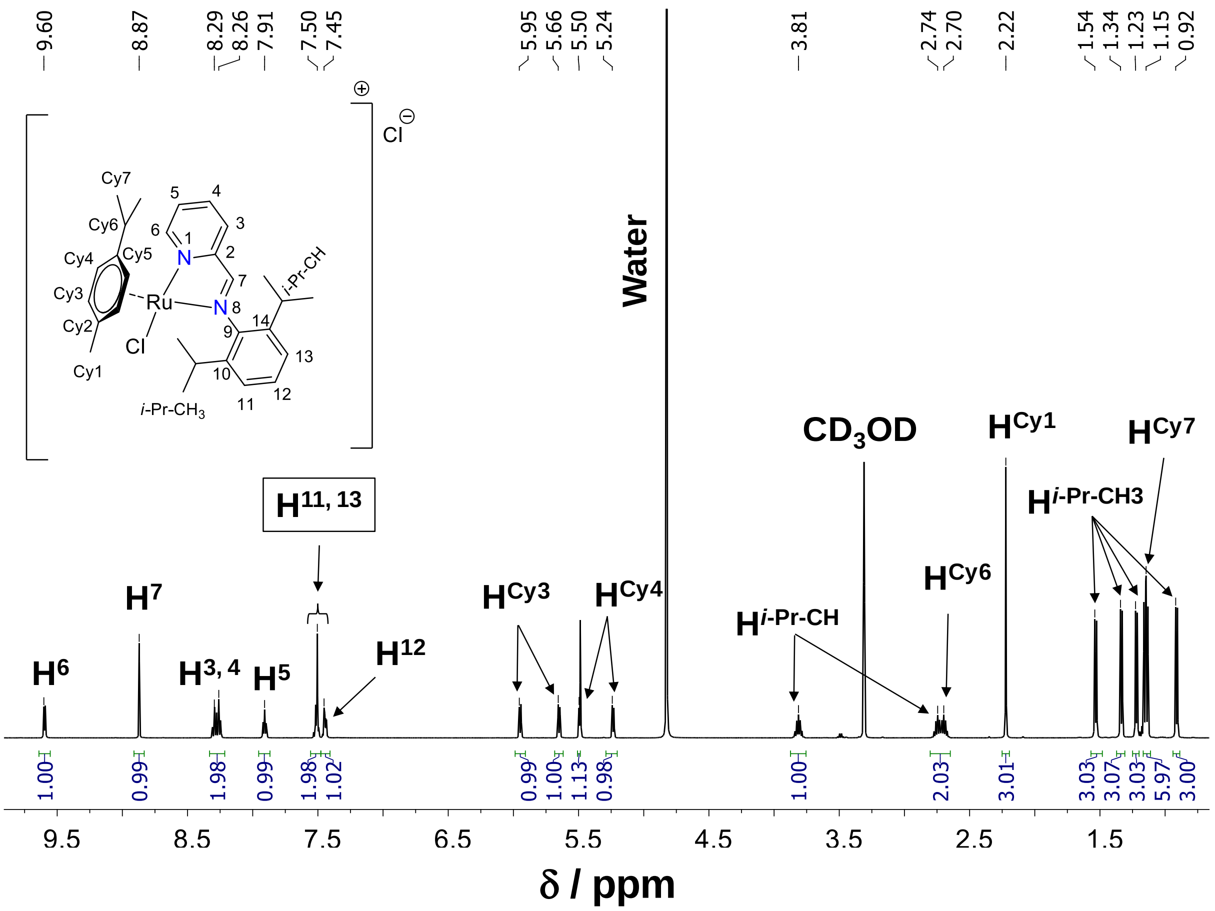


**Figure S14.** $^{1}$H NMR spectrum of [RuCym(DIPMA)Cl]Cl (**4a**) in $CD_3OD$. Inserted structure shows the numbering of peaks. (Peaks highlighted with rectangle are overlapping.)

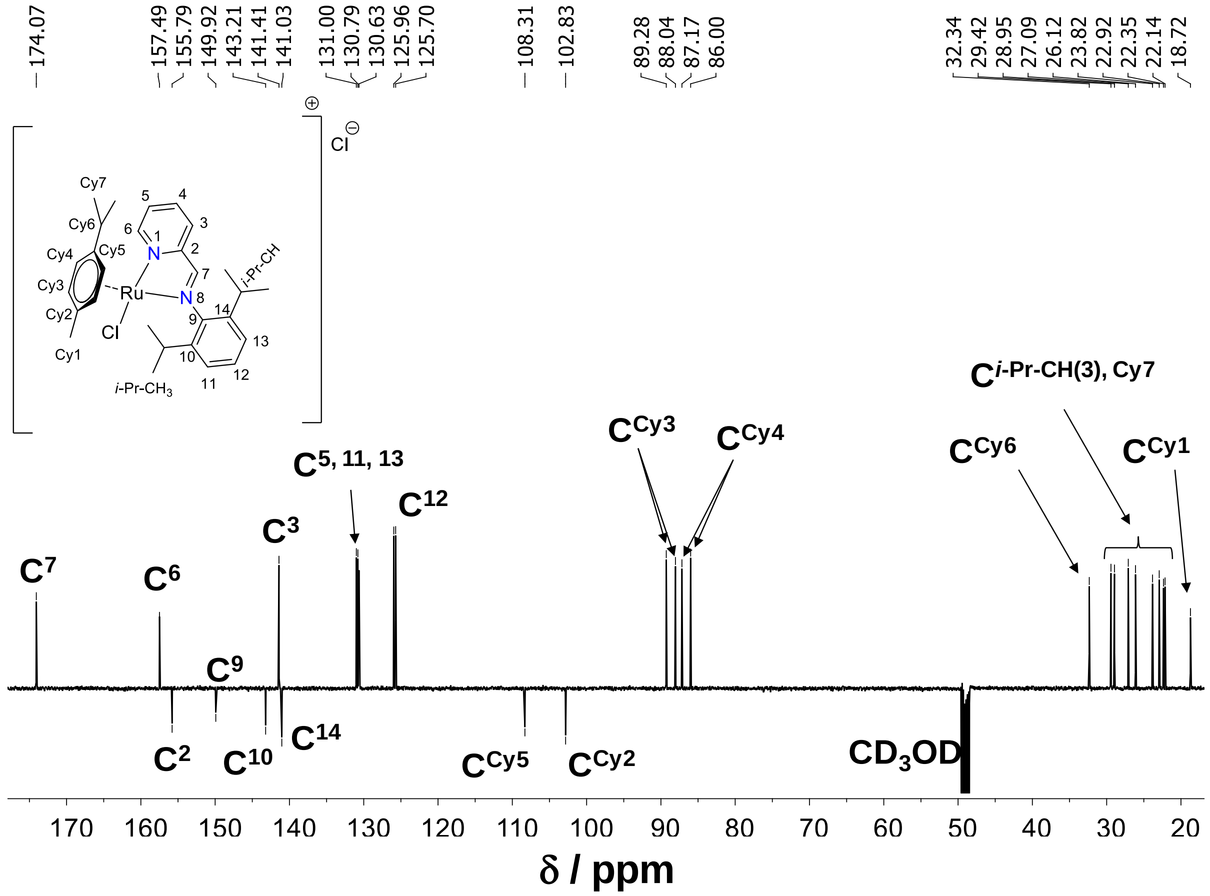


**Figure S15.** $^{13}C$ APT NMR spectrum of [RuCym(DIPMA)Cl]Cl (**4a**) in $CD_3OD$. Attached proton test method: CH and $CH_3$ peaks are positive, C and $CH_2$ peaks are negative.

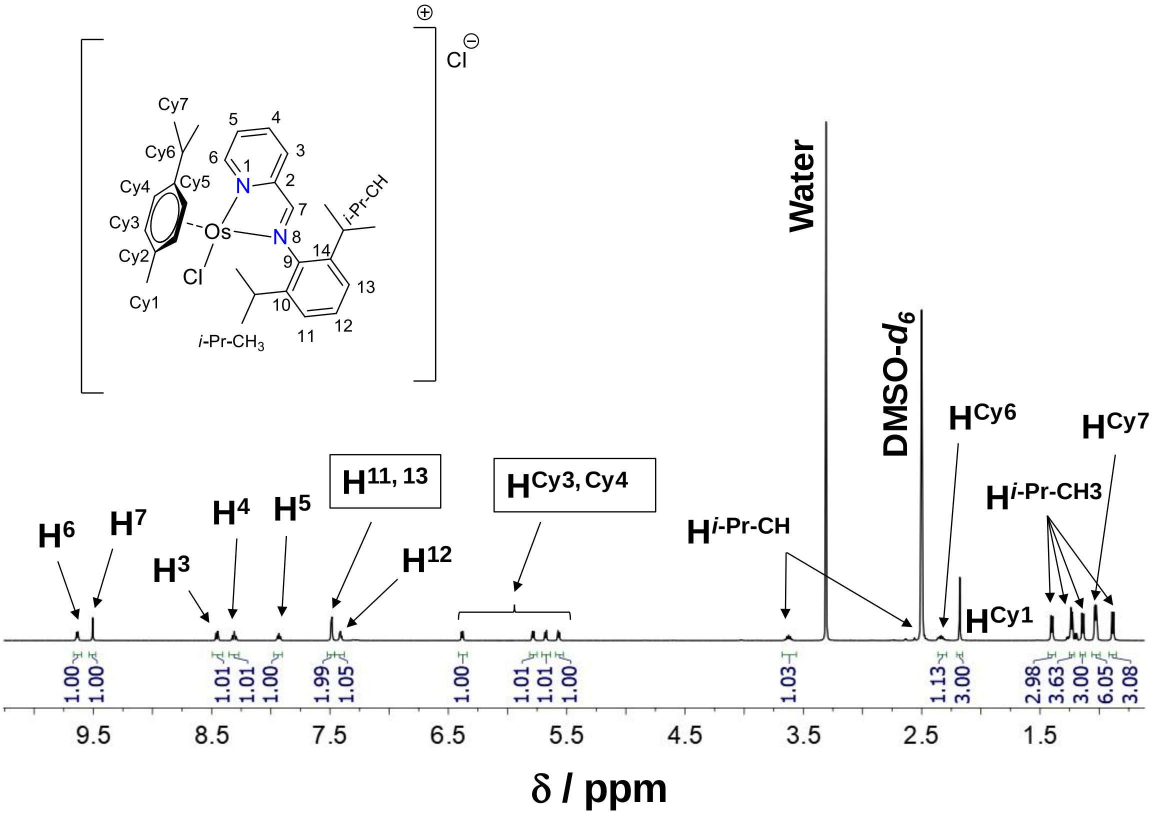


**Figure S16.** $^1$H NMR spectrum of [OsCym(DIPMA)Cl]Cl (**5a**) in DMSO-$d_6$. Inserted structure shows the numbering of peaks. (Peaks highlighted with rectangle are overlapping.)

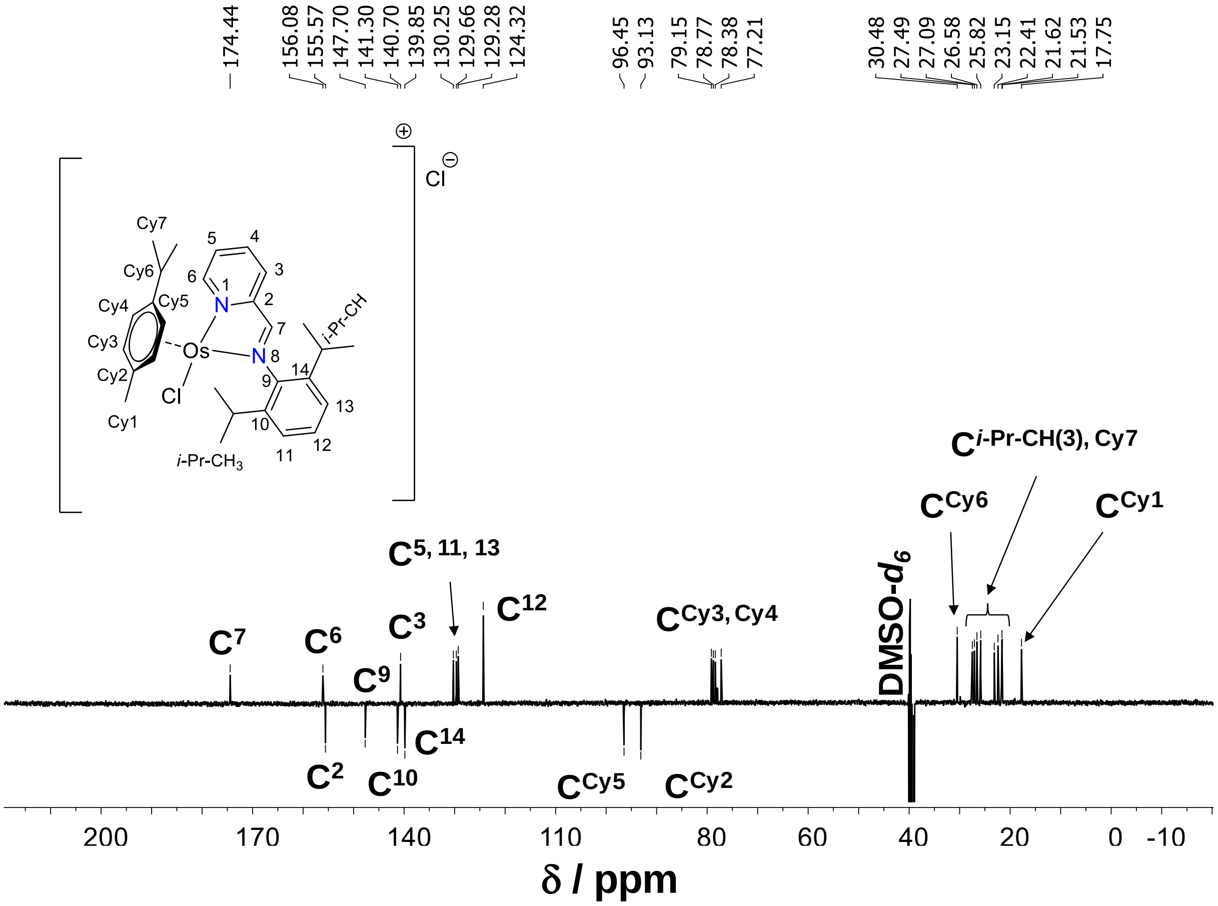


**Figure S17.** $^{13}$C APT NMR spectrum of [OsCym(DIPMA)Cl]Cl (**5a**) in DMSO-$d_6$. Attached proton test method: CH and $CH_3$ peaks are positive, C and $CH_2$ peaks are negative.

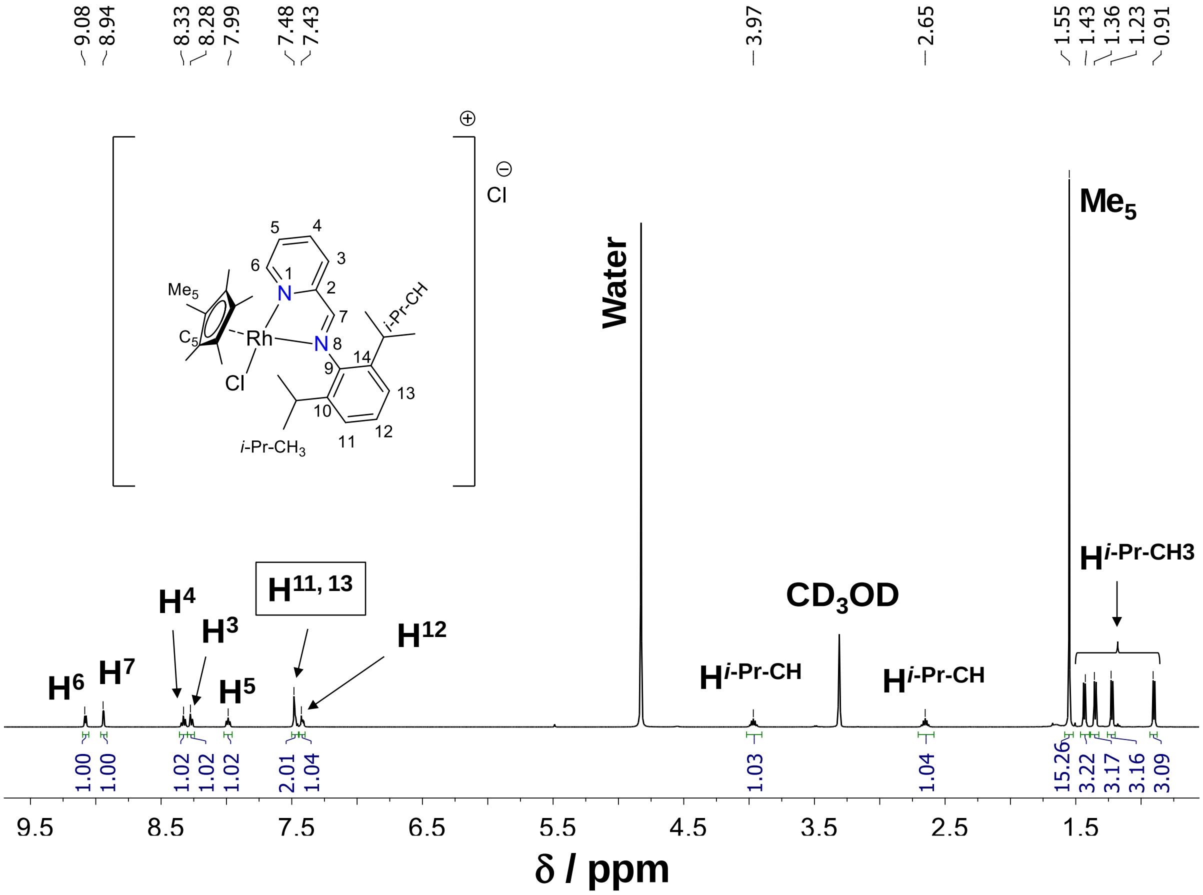


**Figure S18.** $^{1}$H NMR spectrum of [RhCp*(DIPMA)Cl]Cl (**6a**) in $CD_3OD$. Inserted structure shows the numbering of peaks. (Peaks highlighted with rectangle are overlapping.)

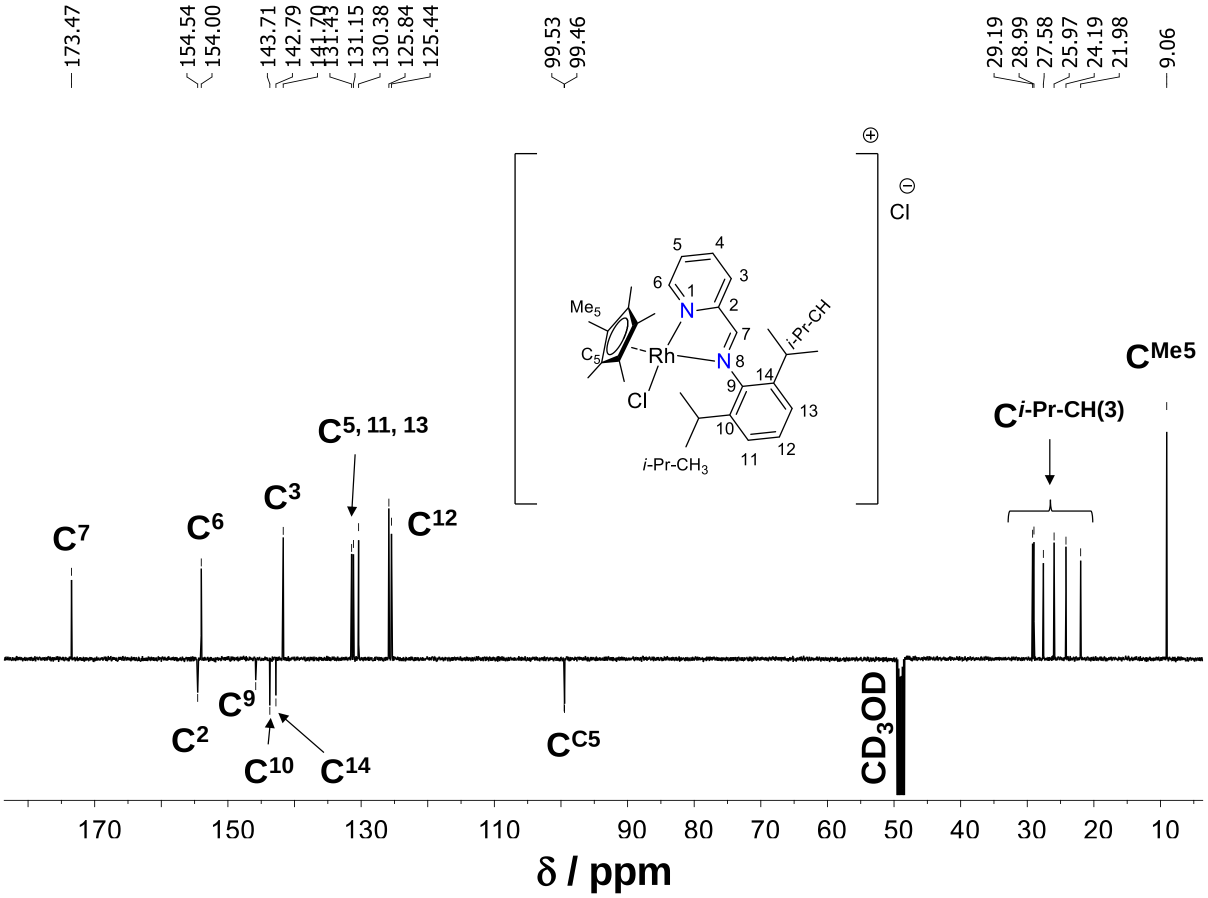


**Figure S19.** $^{13}C$ APT NMR spectrum of [RhCp*(DIPMA)Cl]Cl (**6a**) in $CD_3OD$. Attached proton test method: CH and $CH_3$ peaks are positive, C and $CH_2$ peaks are negative.

**Synthesis and characterization of IQPMA and its intermediates**

**2-Chloracetamido-5-nitrobenzophenone (B)**

To a suspension of 2-amino-5-nitrobenzophenone **(A)** (4.94 g, 20.0 mmol) in chloroform (25 mL) was added in one portion a solution of chloroacetyl chloride (2.39 mL, 30.0 mmol) in chloroform (5 mL) and the reaction mixture was refluxed for 30 min. The solution was cooled down to room temperature and the solvent removed under reduced pressure. The product was crystallized from ethanol (80 mL). Yield: 6.25 g (98%). $^1$H NMR (500 MHz, DMSO-$d_6$), *δ*, ppm: 11.01 (s, 1H), 8.49 (dd, *J* = 9.0, 2.7 Hz, 1H), 8.20 (d, *J* = 2.7 Hz, 1H), 8.05 (d, *J* = 9.0 Hz, 1H), 7.77 (dd, *J* = 8.2, 1.2 Hz, 2H), 7.74–7.68 (m, 1H), 7.57 (dd, *J* = 10.7, 4.8 Hz, 2H), 4.23 (s, 2H). ESI-MS (acetonitrile/methanol + 1% water), positive: *m/z* 319.06 $[M + H^+]^+$ (calcd *m/z* for $[C_{15}H_{11}ClN_2O_4 + H^+]^+$ 319.05).

**2-Azidoacetamido-5-nitrobenzophenone (C)**

A suspension of species **B** (6.24 g, 19.6 mmol) and $NaN_3$ (1.40 g, 29.0 mmol) in DMF (30 mL) was stirred at 60 °C for 30 min. Then water (94 mL) was added dropwise at 60 °C. The mixture was allowed to cool down to room temperature and placed in the fridge at +4 °C to stand overnight. On the next day the raw product was filtered off, washed with water and recrystallized from ethanol (80 mL). Yield: 4.56 g (72%). $^1$H NMR (500 MHz, DMSO-$d_6$) *δ*, ppm: 10.83 (s, 1H), 8.48 (dd, *J* = 9.0, 2.7 Hz, 1H), 8.20 (d, *J* = 2.7 Hz, 1H), 8.03 (d, *J* = 9.0 Hz, 1H), 7.78 – 7.75 (m, 2H), 7.71 (t, *J* = 7.4 Hz, 1H), 7.57 (t, *J* = 7.7 Hz, 2H), 3.98 (s, 2H). ESI-MS (acetonitrile/methanol + 1% water), positive: *m/z* 348.10 $[M+Na^+]^+$ (calcd *m/z* for $[C_{15}H_{11}N_5O_4 + Na^+]^+$: 348.07).

**3-Azido-6-nitro-3-phenylquinolin-2-one (D)**

To the suspension of **C** (12.7 g, 39.0 mmol) in ethanol (250 mL) were added 20 drops of NaOH (40%) and the mixture was stirred at 70 °C overnight. The reaction was allowed to cool to room temperature, then the mixture was stored at −18 °C overnight. On the next day the product was filtered off and washed with ice-cold ethanol. The product was obtained as a yellowish solid after drying in vacuo. Yield: 5.31 g (44%).$^1$H NMR (500 MHz, DMSO-$d_6$) *δ*, ppm: 12.98 (s, 1H), 8.29 (dd, *J* = 9.0, 2.2 Hz, 1H), 7.82 (d, *J* = 2.2 Hz, 1H), 7.61 – 7.53 (m, 4H), 7.43 – 7.38 (m, 2H). ESI-MS (acetonitrile/methanol + 1% water), negative: *m/z* 278.01 $[M–H^+]^–$ (calcd *m/z* for $[C_{15}H_9N_3O_3 – H^+]^–$: 278.06).

**2-Nitroindolo-[2,3-*c*]quinoline-6-one (E)**

A suspension of species **D** (170 mg, 0.55 mmol) in toluene (5 mL) was refluxed for 2 h. The reaction mixture was allowed to cool to room temperature. The product was filtered off and washed with toluene to give a yellow solid. Yield: 129 mg (80%). $^1$H NMR (500 MHz, DMSO-$d_6$) δ 12.68 (s, 1H), 12.45 (s, 1H), 9.07 (d, *J* = 2.4 Hz, 1H), 8.37 (d, *J* = 8.1 Hz, 1H), 8.27 (dd, *J* = 9.0, 2.4 Hz, 1H), 7.68 (d, *J* = 8.3 Hz, 1H), 7.64 (d, *J* = 9.0 Hz, 1H), 7.53 (t, *J* = 7.6 Hz, 1H), 7.41 (t, *J* = 7.5 Hz, 1H). ESI-MS

(acetonitrile/methanol + 1% water), negative: *m/z* 277.99 $[M–H^+]^–$ (calcd *m/z* for $[C_{15}H_9N_3O_3 – H^+]^–$: 278.06).

**2-Aminoindolo-[2,3-*c*]quinoline-6-one (F)**

To a suspension of species **E** (601 mg, 2.15 mmol) in deoxygenated THF (10 mL) was added Pd/C (10%, 229 mg, 0.215 mol) and stirred in $H_2$-atmosphere at 3 bar for 20 h. The reaction mixture was filtered over celite, rinsed with THF and the solvent was removed under reduced pressure to give an off-white solid. Yield: 486.5 mg (91%). $^1$H NMR (500 MHz, DMSO-$d_6$) *δ*, ppm: 12.20 (s, 1H), 11.50 (s, 1H), 8.37 (d, *J* = 8.2 Hz, 1H), 7.66 (d, *J* = 2.3 Hz, 1H), 7.62 (d, *J* = 8.2 Hz, 1H), 7.47 – 7.42 (m, 1H), 7.34 – 7.28 (m, 1H), 7.21 (d, *J* = 8.6 Hz, 1H), 6.73 (dd, *J* = 8.6, 2.3 Hz, 1H), 5.07 (s, 2H). ESI-MS (acetonitrile/methanol + 1% water), positive: *m/z* 250.10 $[M+H^+]^+$ (calcd *m/z* for $[C_{15}H_{11}N_3O + H^+]^+$: 250.10).

**IQPMA**

Through a suspension of species **F** (486 mg, 1.95 mmol) in deoxygenated ethanol (25 mL) 2-formylpyridine (205 µL, 1.1 equiv) was added. The reaction mixture was stirred at 85 °C overnight. On the next day the reaction mixture was cooled down to room temperature, the product was filtered off, washed with EtOH and dried in vacuum to give a yellow solid. Yield: 564 mg (85%). Elemental analysis; calcd for $C_{21}H_{14}N_4O·0.25H_2O$ ($M_r$ 342.87), %: C, 73.56; H, 4.26; N, 16.34. Found, %: C, 73.79; H, 4.09; N, 16.37. ESI-MS (acetonitrile/methanol + 1% water), positive: *m/z* 339.16 $[M+H^+]^+$ (calcd *m/z* for $[C_{21}H_{14}N_4O+H^+]^+$: 339.12). $^1$H NMR (600 MHz, DMSO-$d_6$) *δ* 12.42 (s, 1H, $H^7$), 11.99 (s, 1H, $H^5$), 8.82 (s, 1H, $H^{13}$), 8.75 (ddd, *J* = 4.8, 1.5, 1.0 Hz, 1H, $H^{16}$), 8.55 (d, *J* = 8.1 Hz, 1H, $H^{11}$), 8.32 (d, *J* = 2.1 Hz, 1H, $H^1$), 8.26 (d, *J* = 7.8 Hz, 1H, $H^{19}$), 7.99 (td, *J* = 7.6, 1.5 Hz, 1H, $H^{18}$), 7.66 (d, *J* = 8.3 Hz, 1H, $H^8$), 7.58 (d, *J* = 8.6 Hz, 1H, $H^4$), 7.55 (ddd, *J* = 7.5, 4.8, 1.1 Hz, 1H, $H^{17}$), 7.52 (dd, *J* = 8.6, 2.2 Hz, 1H, $H^3$), 7.50 – 7.47 (m, 1H, $H^9$), 7.37 – 7.30 (m, 1H, $H^{10}$). $^{13}$C NMR (151 MHz, DMSO-$d_6$) δ 159.90 (CH, $C^{13}$), 155.53 (Cq, $C^6$), 154.36 (Cq, $C^{14}$), 149.64 (CH, $C^{16}$), 144.92 (CH, $C^2$), 138.81 (Cq, $C^{7a}$), 136.99 (CH, $C^{18}$), 134.03 (Cq, $C^{4a}$), 127.89 (Cq, $C^{6a}$), 125.75 (CH, $C^9$), 125.45 (CH, $C^{17}$), 122.50 (CH, $C^{11}$), 122.23 (Cq, $C^{11a}$), 121.19 (CH, $C^{19}$), 120.82 (CH, $C^{10}$), 119.72 (CH, $C^3$), 118.59 (Cq, $C^{11c}$), 117.99 (Cq, $C^{11b}$), 117.02 (CH, $C^4$), 115.26 (CH, $C^1$), 113.04 (CH, $C^8$).

**Synthesis and characterization of the half-sandwich complexes**

**General method**

The synthesis of the half-sandwich complexes (**1a**–**6a**) was carried out according to procedure described for analogous (N,N)-coordinated compounds,[39,40] by reacting one equivalent of the dinuclear precursor [Ru($\eta^6$-*p*-cymene)$Cl_2$]$_2$, [Os($\eta^6$-*p*-cymene)$Cl_2$]$_2$ or [Rh($\eta^5$-$C_5Me_5$)$Cl_2$]$_2$ (dissolved in $CHCl_3$ or $CH_2Cl_2$) with two equivalents of the ligand IQPMA or DIPMA (dissolved in $CHCl_3$ or $CH_2Cl_2$ or methanol).

**Synthesis of [RuCym(IQPMA)Cl]Cl (1a)**

To a suspension of IQPMA (52.1 mg, 0.15 mmol) in MeOH (100 mL) at 60 °C [Ru(*p*-cymene)$Cl_2$]$_2$ (47.2 mg, 77 µmol) in $CHCl_3$ (3 mL) was added and the reaction mixture was stirred at 55 °C for 1 h. The solvent was reduced to *ca*. 1/3 and the product was precipitated with diethyl ether. The flask was placed in the fridge at +4 °C and left to stand overnight. Orange crystalline solid was filtered off, washed with diethyl ether (2 mL) and dried in air. Yield: 68.3 mg (64%). Elemental analysis; calcd for $C_{31}H_{28}Cl_2N_4ORu \times 3H_2O$ ($M_r$ 698.60), %: C, 53.29; H, 4.90; N, 8.01. Found, %: C, 53.34; H, 4.61; N, 8.13. ESI-MS (methanol, positive): calc. for [RuCym(IQPMA)Cl]$^+$ ($C_{31}H_{28}ClN_4ORu$): 609.10 (*m/z*); found: 609.10 (*m/z*). $^1$H NMR (600 MHz, DMSO-$d_6$, Figure S8) $\delta$, ppm: 12.60 (s, 1H, H$^7$), 12.28 (s, 1H, H$^5$), 9.64 (d, $J$ = 5.5 Hz, 1H, H$^{16}$), 9.06 (s, 1H, H$^{13}$), 8.86 (d, $J$ = 2.0 Hz, 1H, H$^1$), 8.61 (d, $J$ = 8.2 Hz, 1H, H$^{11}$), 8.35 (d, $J$ = 4.3 Hz, 2H, H$^{18}$, H$^{19}$), 7.93 – 7.87 (m, 2H, H$^3$, H$^{17}$), 7.71 (dd, $J$ = 8.4, 5.8 Hz, 2H, H$^4$, H$^8$), 7.54 (t, $J$ = 7.6 Hz, 1H, H$^9$), 7.40 (t, $J$ = 7.5 Hz, 1H, H$^{10}$), 6.13 (d, $J$ = 6.2 Hz, 1H, H$^{Cy4}$), 5.80 (d, $J$ = 6.2 Hz, 1H, H$^{Cy3}$), 5.73 (d, $J$ = 6.1 Hz, 1H, H$^{Cy4}$), 5.54 (d, $J$ = 6.0 Hz, 1H, H$^{Cy3}$), 2.55 (dt, $J$ = 11.4, 5.7 Hz, 1H, H$^{Cy6}$), 2.21 (s, 3H, H$^{Cy1}$), 1.01 (dd, $J$ = 6.8, 5.1 Hz, 6H, H$^{Cy7}$). $^{13}$C NMR (151 MHz, DMSO-$d_6$, Figure S9) δ 167.24 (CH, C$^{13}$), 156.06 (CH, C$^{16}$), 155.70 (Cq, C$^6$), 154.84 (Cq, C$^{14}$), 146.53 (Cq, C$^2$), 139.90 (CH, C$^{18}$), 138.95 (Cq, C$^{7a}$), 135.67 (Cq, C$^{4a}$), 129.94 (CH, C$^{19}$), 128.72 (CH, C$^{17}$), 128.19 (Cq, C$^{6a}$), 126.11 (CH, C$^9$), 122.09 (CH, C$^{11}$; Cq, C$^{11a}$), 121.01 (CH, C$^{10}$), 120.59 (CH, C$^3$), 118.04 (Cq, C$^{11c}$), 117.72 (Cq, C$^{11b}$), 116.82 (CH, C$^4$), 116.44 (CH, C$^1$), 113.41 (CH, C$^8$), 105.09 (Cq, C$^{Cy5}$), 103.87 (Cq, C$^{Cy2}$), 86.78 (CH, C$^{Cy4}$), 85.93 (CH, C$^{Cy4}$), 84.95 (CH, C$^{Cy3}$), 64.91 (CH, C$^{Cy3}$), 30.50 (CH, C$^{Cy6}$), 21.93 (2x $CH_3$, C$^{Cy7}$), 18.42 ($CH_3$, C$^{Cy1}$).

**Synthesis of [OsCym(IQPMA)Cl]Cl (2a)**

To a suspension of IQPMA (57.0 mg, 0.17 mmol) in MeOH (50 mL) at 55 °C [Os(*p*-cymene)$Cl_2$]$_2$ (66.6 mg, 84 µmol) in $CHCl_3$ (1.5 mL) was added and the reaction mixture was stirred at 55 °C for 1 h. The solvent was reduced to *ca*. 15 mL and the product was precipitated with diethyl ether. The flask was placed in the fridge at +4 °C and left to stand for 2 h. The red product was filtered off, washed with diethyl ether (2 mL) and dried in high vacuum. Yield: 116.6 mg (90%). Elemental analysis; calcd for $C_{31}H_{28}Cl_2N_4OOs \times 2.5H_2O$ ($M_r$ 778.76), %: C, 47.81; H, 4.27; N, 7.19. Found, %: C, 47.65; H, 3.94; N, 7.15. ESI-MS (methanol, positive): calc. for [OsCym(IQPMA)Cl]$^+$ ($C_{31}H_{28}ClN_4OOs$): 699.1561 (*m/z*); found: 699.1577 (*m/z*). $^1$H NMR (600 MHz, DMSO-$d_6$, Figure S10) δ 12.60 (s, 1H, H$^7$), 12.27 (s, 1H, H$^5$), 9.57 (d, $J$ = 5.6 Hz, 1H, H$^{16}$), 9.44 (s, 1H, H$^{13}$), 8.76 (d, $J$ = 2.2 Hz, 1H, H$^1$), 8.55 (d, $J$ = 8.2 Hz, 1H, H$^{11}$), 8.46 (d, $J$ = 7.3 Hz, 1H, H$^{19}$), 8.31 (td, $J$ = 7.8, 1.2 Hz, 1H, H$^{18}$), 7.86 (ddd, $J$ = 7.5, 5.8, 1.4 Hz, 1H, H$^{17}$), 7.80 (dd, $J$ = 8.7, 2.3 Hz, 1H, H$^3$), 7.70 (dd, $J$ = 8.4, 6.3 Hz, 2H, H$^4$, H$^8$), 7.53 (t, $J$ = 7.4 Hz, 1H, H$^9$), 7.39 (t, $J$ = 7.5 Hz, 1H, H$^{10}$), 6.41 (d, $J$ = 5.8 Hz, 1H, H$^{Cy4}$), 6.02 (d, $J$ = 5.8 Hz, 1H, H$^{Cy3}$), 5.93 (d, $J$ = 5.7 Hz, 1H, H$^{Cy4}$), 5.69 (d, $J$ = 5.7 Hz, 1H, H$^{Cy3}$), 2.43 (dt, $J$ = 13.7, 7.0 Hz, 1H, H$^{Cy6}$), 2.29 (s, 3H, H$^{Cy3}$), 0.94 (t, $J$ = 7.1 Hz, 6H, 2x H$^{Cy7}$). $^{13}$C NMR (151 MHz, DMSO-$d_6$, Figure S11) δ 168.56 (CH, C$^{13}$), 156.68 (Cq, C$^{14}$), 156.20 (CH, C$^{16}$), 156.18 (Cq, C$^6$), 147.00 (Cq, C$^2$), 140.53 (CH, C$^{18}$),

139.43 (Cq, $C^{7a}$), 136.26 (Cq, $C^{4a}$), 130.28 (CH, $C^{19}$), 130.03 (CH, $C^{17}$), 128.65 (Cq, $C^{6a}$), 126.58 (CH, $C^{9}$), 122.53 (Cq, $C^{11a}$), 122.50 (CH, $C^{11}$), 121.53 (CH, $C^{10}$), 121.48 (CH, $C^{3}$), 118.48(Cq, C11c), 118.13 (Cq, C11b), 117.31 (CH, C4), 117.21 (CH, C1), 113.88 (CH, C8), 98.45 (Cq, CCy2), 97.21 (Cq, $C^{Cy5}$), 79.43 (CH, $C^{Cy4}$), 78.41 (CH, $C^{Cy4}$), 75.89 (CH, $C^{Cy3}$), 75.71 (CH, $C^{Cy3}$), 31.20 (CH, $C^{Cy6}$), 22.63 ($CH_3$, $C^{Cy7}$), 22.32 ($CH_3$, $C^{Cy7}$), 18.88 ($CH_3$, $C^{Cy1}$).

**Synthesis of [RhCp*(IQPMA)Cl]Cl (3a)**

The precursor [Rh($\eta^5$-$C_5Me_5$)$Cl_2$]$_2$ (10.5 mg, 17.0 µmol) and IQPMA (11.5 mg, 33.5 µmol) were reacted in $CH_2Cl_2$ at room temperature for 24 h. The solvent was partly evaporated under reduced presure, and the final product was obtained by the addition of diethyl ether, followed by vacuum filtration and washing with diethyl ether and *n*-hexane. The yellow solid was then dried at 45 °C for 4 h. Yield: 13.5 mg (57%). Elemental analysis; calcd for $C_{31}H_{29}Cl_2N_4ORh$×3 $H_2O$ ($M_r$ = 701.45), %: C, 53.08; H, 5.03; N, 7.99; O, 9.12. Found, %: C, 53.51; H, 4.68; N, 7.91; O, 8.85. ESI-MS (methanol, positive): calc. for $[RhCp^*(IQPMA)Cl]^+$ ($C_{31}H_{29}ClN_4ORh$): 611.1085 (*m/z*); found: 611.1091 (*m/z*). $^1$H NMR (DMSO-$d_6$, δ/ppm, 500 MHz, Figure S12): 12.59 (s, 1H, $H^7$), 12.23 (s, 1H, $H^5$), 9.12 (d, $J$ = 5.4 Hz, 1H, $H^{16}$), 9.09 (s, 1H, $H^{13}$), 8.89 (d, 1H, $H^1$), 8.61 (d, $J$ = 8.2 Hz, 1H, $H^{11}$), 8.42 – 8.37 (m, 2H, $H^{18}$, $H^{19}$), 8.00 (dt, $J$ = 6.3, 1.6 Hz, 1H, $H^{17}$), 7.78 (dd, $J$ = 8.6, 2.0 Hz, 1H, $H^3$), 7.70 (dd, $J$ = 8.5, 3.1 Hz, 2H, $H^4$, $H^8$), 7.52 (t, $J$ = 7.7 Hz, 1H, $H^9$), 7.38 (t, $J$ = 7.6 Hz, 1H, $H^{10}$), 1.42 (s, 15H, C5Me5). $^{13}$C NMR (DMSO-$d_6$, δ/ppm, 151 MHz, Figure S13): 167.18 (CH, $C^{13}$), 155.63 (Cq, $C^{14}$), 154.07 (Cq, $C^6$), 152.85 (CH, $C^{16}$), 142.93 (Cq, $C^2$), 140.53 (CH, $C^{19}$), 138.95 (Cq, $C^{7a}$), 135.33 (Cq, $C^{4a}$), 129.82 (CH, $C^{18}$), 129.75 (CH, $C^{17}$), 128.12 (Cq, $C^{6a}$), 126.09 (CH, $C^4$), 122.02 (CH, $C^3$), 121.93 (Cq, $C^{11b}$), 120.99 (CH, $C^1$), 120.01 (CH, $C^{11}$), 118.02 (Cq, $C^{11a}$), 117.52 (Cq, $C^{11c}$), 117.17 (CH, $C^9$), 116.66 (CH, $C^{10}$), 113.40 (CH, $C^8$), 97.10 (Cq, $C^{C5}$), 97.04 (Cq, $C^{C5}$), 8.32 ($CH_3$, $C^{Me5}$).

**Synthesis of [RuCym(DIPMA)Cl]Cl (4a)**

The precursor [Ru($\eta^6$-*p*-cymene)$Cl_2$]$_2$ (35.2 mg, 57.5 µmol) and DIPMA (30.6 mg, 114.9 µmol) were reacted in $CH_2Cl_2$ at room temperature for 24 h. Then the solvent was partly evaporated, and the final product was obtained by the addition of diethyl ether, followed by vacuum filtration and washing with diethyl ether and *n*-hexane. The orange solid was dried at 45 °C for 4 h. Yield: 57.3 mg (83%). Elemental analysis; calcd for $C_{28}H_{36}Cl_2N_2Ru$×1.5$H_2O$ ($M_r$ = 599.60), %: C, 56.09; H, 6.56; N, 4.67; O, 4.00. Found, %: C, 56.33; H, 6.25; N, 4.66; O, 4.32. ESI-MS (methanol, positive): calc. for $[RuCym(DIPMA)Cl]^+$ ($C_{28}H_{36}ClN_2Ru$): 537.1611 (*m/z*); found: 537.1627 (*m/z*). $^1$H NMR ($CD_3OD$, δ/ppm, 500 MHz, Figure S14): 9.60 (d, $J$ = 5.5 Hz, 1H, $H^6$), 8.87 (s, 1H, $H^7$), 8.31 – 8.25 (m, 2H, $H^3$, $H^4$), 7.91 (m, 1H, $H^5$), 7.53 – 7.49 (m, 2H, $H^{11}$, $H^{13}$), 7.44 (dd, $J$ = 6.2; 3.0 Hz, 1H, $H^{12}$), 5.95 (d, $J$ = 6.4 Hz, 1H, $H^{Cy3}$), 5.65 (d, $J$ = 6.4 Hz, 1H, $H^{Cy3}$), 4.59 (d, $J$ = 4.4 Hz, 1H, $H^{Cy4}$), 5.24 (d, $J$ = 6.1 Hz, 1H, $H^{Cy4}$), 3.81 (m, 1H, $H^{i\text{-Pr-CH}}$), 2.75 (m, 1H, $H^{i\text{-Pr-CH}}$), 2.70 (m, 1H, $H^{Cy6}$), 2.22 (s, 1H, $H^{Cy1}$), 1.53 (d, $J$ = 6.8 Hz, 3H, $H^{i\text{-Pr-CH3}}$), 1.34 (d, $J$ = 6.7 Hz, 3H, $H^{i\text{-Pr-CH3}}$), 1.22 (d, $J$ = 6.7 Hz, 3H, $H^{i\text{-Pr-CH3}}$), 1.15 (dd, $J$ = 8.5, 7.1 Hz, 6H, $H^{Cy7}$), 0.91 (d,

*J* = 6.7 Hz, 3H, $H^{i\text{-Pr-CH3}}$). $^{13}C$ NMR ($CD_3OD$, δ/ppm, 151 MHz, Figure S15): 174.07 (CH, $C^7$), 157.49 (CH, $C^6$), 155.79 (Cq, $C^2$), 149.92 (Cq, $C^9$), 143.21 (Cq, $C^{10}$), 141.41 (CH, $C^3$), 141.03 (Cq, $C^{14}$), 131.00 (CH, $C^5$), 130.79 (CH, $C^{11}$), 130.63 (CH, $C^{13}$), 125.96, 125.70 (CH, $C^{12}$), 108.31 (Cq, $C^{Cy5}$), 102.83 (Cq, $C^{Cy2}$), 89.28 (CH, $C^{Cy3}$), 88.04 (CH, $C^{Cy3}$), 87.17 (CH, $C^{Cy4}$), 86.00 (CH, $C^{Cy4}$), 32.24 (CH, $C^{Cy6}$), 29.42 (CH, $C^{i\text{-Pr-CH}}$), 28.95 (CH, $C^{i\text{-Pr-CH}}$), 27.09 ($CH_3$, $C^{Cy7}$), 26.12 ($CH_3$, $C^{Cy7}$), 23.82 ($CH_3$, $C^{i\text{-Pr-CH3}}$), 22.93 ($CH_3$, $C^{i\text{-Pr-CH3}}$), 22.35 ($CH_3$, $C^{i\text{-Pr-CH3}}$), 22.14 ($CH_3$, $C^{i\text{-Pr-CH3}}$), 18.72 ($CH_3$, $C^{Cy1}$).

**Synthesis of [OsCym(DIPMA)Cl]Cl (5a)**

The [Os($\eta^6$-*p*-cymene)$Cl_2$]$_2$ (10.8 mg, 13.7 µmol) and DIPMA (7.3 mg, 27.4 µmol) were reacted in $CH_2Cl_2$ at room temperature for 24 h. After partial evaporation of the solvent under reduced pressure, the product was precipitated by addition of diethyl ether, filtered under vacuum, washed with diethyl ether and *n*-hexane, and dried at 45 °C for 4 h to give a red solid. Yield: 10.4 mg (51%). Elemental analysis; calcd for $C_{28}H_{36}Cl_2N_2Os\times CH_2Cl_2$ ($M_r$ = 746.67), %: C, 46.65; H, 5.13; N, 3.75. Found, %: C, 46.59; H, 5.28; N, 3.68. ESI-MS (methanol, positive): calc. for $[OsCym(DIPMA)Cl]^+$ ($C_{28}H_{36}ClN_2Os$): 627.2182 (*m/z*); found: 627.2176 (*m/z*). $^1H$ NMR (DMSO-$d_6$, δ/ppm, 500 MHz, Figure S16): 9.64 (d, *J* = 5.6 Hz, 1H, $H^6$), 9.51 (s, 1H, $H^7$), 8.46 (d, *J* = 7.6 Hz, 1H, $H^3$), 8.31 (t, *J* = 7.8 Hz, 1H, $H^4$), 7.93 (t, *J* = 6.4 Hz, 1H, $H^5$), 7.49 (d, *J* = 4.6 Hz, 2H, $H^{11}$, $H^{13}$), 7.41 (t, *J* = 4.6 Hz, 1H, $H^{12}$), 6.38 (d, *J* = 6.0 Hz, 1H, $H^{Cy3}$), 5.78 (d, *J* = 6.0 Hz, 1H, $H^{Cy3}$), 5.68 (d, *J* = 5.8 Hz, 1H, $H^{Cy4}$), 5.57 (d, *J* = 5.8 Hz, 1H, $H^{Cy4}$), 3.63 (m, 1H, $H^{i\text{-Pr-CH}}$), 2.48 (m, Under solvent peak, 1H, $H^{i\text{-Pr-CH}}$), 2.34 (m, 1H, $H^{Cy6}$), 2.18 (s, 3H, $H^{Cy1}$), 1.40 (d, *J* = 7.0 Hz, 3H, $H^{i\text{-Pr-CH3}}$), 1.23 (d, *J* = 6.6 Hz, 3H, $H^{i\text{-Pr-CH3}}$), 1.14 (d, *J* = 6.7 Hz, 3H, $H^{i\text{-Pr-CH3}}$), 1.03 (dd, *J* = 6.8, 2.7 Hz, 6H, $H^{Cy7}$), 0.89 (d, *J* = 6.7 Hz, 3H, $H^{i\text{-Pr-CH3}}$). $^{13}C$ NMR (DMSO-$d_6$, δ/ppm, 151 MHz, Figure S17): 177.44 (CH, $C^7$), 156.08 (CH, $C^6$), 155.57 (Cq, $C^2$), 147.70 (Cq, $C^9$), 141.30 (Cq, $C^{10}$), 140.70 (CH, $C^3$), 139.85 (Cq, $C^{14}$), 130.25 (CH, $C^5$), 129.66 (CH, $C^{11}$), 129.29 (CH, $C^{13}$), 124.32 (CH, $C^{12}$), 96.46 (Cq, $C^{Cy5}$), 93.13 (Cq, $C^{Cy2}$), 79.15 (CH, $C^{Cy3}$), 78.77 (CH, $C^{Cy3}$), 78.38 (CH, $C^{Cy4}$), 77.22 (CH, $C^{Cy4}$), 30.48 (CH, $C^{Cy6}$), 27.49 (CH, $C^{i\text{-Pr-CH}}$), 27.09 (CH, $C^{i\text{-Pr-CH}}$), 26.58 ($CH_3$, $C^{Cy7}$), 25.82 ($CH_3$, $C^{Cy7}$), 23.15 ($CH_3$, $C^{i\text{-Pr-CH3}}$), 22.41 ($CH_3$, $C^{i\text{-Pr-CH3}}$), 21.62 ($CH_3$, $C^{i\text{-Pr-CH3}}$), 21.53 ($CH_3$, $C^{i\text{-Pr-CH3}}$), 17.75 ($CH_3$, $C^{Cy1}$).

**Synthesis of [RhCp*(DIPMA)Cl]Cl (6a)**

The complex was prepared with the same procedure used for complex **3a**, using [Rh($\eta^5$-$C_5Me_5$)$Cl_2$]$_2$ (11.2 mg, 18.1 µmol) and DIPMA (9.6 mg, 36.0 µmol). Yield: 16.0 mg (72%). Elemental analysis; calcd for $C_{28}H_{37}Cl_2N_2Rh\times 1.75H_2O\times 0.15CH_2Cl_2$ ($M_r$ = 619.70), %: C, 55.30; H, 6.52; N, 4.58. Found, %: C, 55.31; H, 6.10; N, 4.52. ESI-MS (methanol, positive): calc. for $[RhCp^*(DIPMA)Cl]^+$ ($C_{28}H_{37}ClN_2Rh$): 539.1700 (*m/z*); found: 539.1694 (*m/z*). $^1H$ NMR ($CD_3OD$, δ/ppm, 500 MHz, Figure S18): 9.08 (d, *J* = 5.3 Hz, 1H, $H^6$), 8.94 (d, *J* = 2.7 Hz, 1H, $H^7$), 8.33 (dt, *J* = 7.7; 1.1 Hz, 1H, $H^4$), 8.27 (d, *J* = 7.0 Hz, 1H, $H^3$), 7.99 (dddd, 1H, $H^5$), 7.49 – 7.46 (m, 2H, $H^{11}$, $H^{13}$), 7.42 (dd, *J* = 6.0; 3.2 Hz, 1H, $H^{12}$), 3.97 (m, 1H,

$H^{i\text{-Pr-CH}}$), 2.65 (m, 1H, $H^{i\text{-Pr-CH}}$), 1.55 (s, 15H, C5Me5), 1.43 (d, *J* = 6.7 Hz, 3H, $H^{i\text{-Pr-CH3}}$), 1.35 (d, *J* = 6.8 Hz, 3H, $H^{i\text{-Pr-CH3}}$), 1.22 (d, *J* = 6.7 Hz, 3H, $H^{i\text{-Pr-CH3}}$), 0.90 (d, *J* = 6.6 Hz, 3H, $H^{i\text{-Pr-CH3}}$). $^{13}$C NMR ($CD_3OD$, δ/ppm, 151 MHz, Figure S19): 173.47 (CH, $C^7$), 154.54 (Cq, $C^2$), 154.00 (CH, $C^6$), 145.82 (Cq, $C^9$), 143.71 (Cq, $C^{10}$), 142.79 (Cq, $C^{14}$), 141.70 (CH, $C^3$), 131.43 (CH, $C^5$), 131.16 (CH, $C^{11}$), 130.38 (CH, $C^{13}$), 125.84, 125.45 (CH, $C^{12}$), 99.53 (Cq, $C^{C5}$), 99.46 (Cq, $C^{C5}$), 29.19 (CH, $C^{i\text{-Pr-CH}}$), 28.99 (CH, $C^{i\text{-Pr-CH}}$), 27.58 ($CH_3$, $C^{i\text{-Pr-CH3}}$), 25.97 ($CH_3$, $C^{i\text{-Pr-CH3}}$), 24.19 ($CH_3$, $C^{i\text{-Pr-CH3}}$), 21.99 ($CH_3$, $C^{i\text{-Pr-CH3}}$), 9.07 ($CH_3$, $C^{Me5}$).

**Methods used for the characterization of the isolated solid compounds**

**NMR Spectroscopy**

NMR spectroscopy was performed on Bruker Avance III HD Ascend 500 Plus or BrukerAV III 600 or AV NEO 500 spectrometer at room temperature. $^1$H and $^{13}$C NMR spectra were recorded at 600.25/500.10 MHz and 150.95 MHz, respectively. Chemical shifts are referenced to residual peaks of the deuterated solvent MeOD-$d_4$ at 3.31 ($^1$H) and 49.00 ($^{13}$C) ppm or DMSO-$d_6$ at 2.50 ($^1$H) and 39.52 ($^{13}$C) ppm. Chemical shifts (δ) and coupling constants (J) are given in ppm and Hz, respectively.

**Electrospray Ionization Mass Spectrometry**

A Waters Q-TOF Premier (Micromass MS Technologies) or a Bruker maXis ESI-Qq-TOF mass spectrometer with an electrospray ion source was used to perform high-resolution or low-resolution ESI-MS experiments, respectively. Samples contained 100 µM compounds (ligand or complex) in methanol (LC-MS grade). Accordingly, *m*/*z* values are given with four decimal places for high-resolution MS (**2a**−**6a**) and with two decimal places for low-resolution (IQPMA, **1a**) measurements.

**Elemental analysis**

Elemental analysis was performed on a Perkin Elmer 2400 CHN Elemental Analyzer at the Microanalytical Laboratory of the University of Vienna.

**Infrared Spectroscopy**

The FT-IR spectra of complexes **1a**−**4a** (see Figure S57) were recorded on an ALPHA FT-IR (Bruker) spectrometer using the KBr pellet technique. 4-5 mg of sample was finely ground with 100 mg of KBr and pressed into pellets. Prior to pellet preparation, KBr was dried at 130 °C for 12 h in a drying oven. The background spectrum was recorded using the same dried KBr matrix.

## X-ray data collection, structure solution and refinement

X-ray diffraction data were collected on a Rigaku RAXIS-RAPID II diffractometer except of crystals **I** and **3b** which were measured on a XtaLAB Synergy R HyPix diffractometer. All crystals were measured

using Cu-Kα radiation at low temperature (100-113 K) except **6b** which were measured at room temperature (293 K). At 103 K the crystals of **6b** broke apart, that is why the measurement were only possible at room temperature. Numerical absorption correction was carried out using RAPID AUTO [SI-1] except for crystals **I** and **3b** for which empirical absorption correction was carried out using the program CrysAlisPro [SI-2]. In all cases, software Olex2 [SI-3] were used to solve the structures by SHELXS [SI-4] structure solution program using Direct Methods and refined with the olex2.refine [SI-5] refinement package using Gauss-Newton minimization. Refinement of non-hydrogen atoms was carried out with anisotropic temperature factors. Hydrogen atoms were placed into geometric positions. They were included in structure factor calculations but they were not refined. The isotropic displacement parameters of the hydrogen atoms were approximated from the U(eq) value of the atom they were bonded to. In case of crystals **1b** and **2b**, we found residual electron density in the difference Fourier map that we could not clearly assign to a solvent. 32 $e^-$ per asymmetric unit in the cavities of crystal **1a** were taken into account with the solvent mask option during the refinement. This can be assigned to 0.8 diethyl-ether molecule per asymmetric unit. Similarly, in crystal **2b**, 54 $e^-$ per asymmetric unit were taken into account with the squeeze option during the refinement. This is consistent with the presence of disorder 1.3 diethyl-ether solvents per asymmetric unit. In this crystal, $PF_6^-$ counter ion was found in two disorder position: F1-F6 was refined with occupancy of 66.7% and F1a – F6a with 33.3%. The summary of data collection and refinement parameters are collected in Tables S1 and S2. Graphical representations were generated using Mercury [SI-6].

**Table S1.** Crystal data and structure refinement for crystals **I**, **1b** and **2b**.

| Identification code | IQPMA (**I**) | [RuCym(IQPMA)Cl]$PF_6$×DMF (**1b**×DMF) | [OsCym(IQPMA)Cl] $PF_6$ ×DMF (**2b**×DMF) |
|---|---|---|---|
| CCDC number | 2529816 | 2529817 | 2529818 |
| Empirical formula | $C_{21}H_{14}N_4O$ | $C_{34}H_{35}ClF_6N_5O_2PRu$ [+solvent] | $C_{38}H_{43}ClF_6N_5O_3OsP$ [+ solvent] |
| Moiety formula | $C_{21}H_{14}N_4O$ | $C_{31}H_{28}ClN_4ORu$, $PF_6$, $C_3H_7NO$ [+ solvent] | $C_{31}H_{28}ClN_4OOs$, $PF_6$, $C_3H_7NO$ [+ solvent] |
| Formula weight | 338.372 | 901.294 | 988.438 |
| Temperature/K | 100.0(5) | 103(2) | 110(2) |
| Crystal system | monoclinic | triclinic | triclinic |
| Space group | $P2_1/n$ | *P*-1 | *P*-1 |
| a/Å | 18.3666(16) | 9.2052(2) | 9.2877(2) |
| b/Å | 4.3459(9) | 11.9382(3) | 14.7234(3) |
| c/Å | 20.1714(10) | 18.4452(4) | 16.5998(3) |
| α/° | 90 | 81.185(6) | 65.362(5) |
| β/° | 92.129(5) | 75.684(5) | 85.382(6) |
| γ/° | 90 | 86.192(6) | 84.828(6) |
| Volume/Å$^3$ | 1609.0(4) | 1940.00(9) | 2052.59(11) |
| Z/Z' | 4/1 | 2/1 | 2/1 |
| $\rho_{calc}$ / g/cm$^3$ | 1.397 | 1.543 | 1.599 |
| μ/mm$^{-1}$ | 0.720 | 4.936 | 7.450 |
| F(000) | 706.3 | 928.8 | 979.7 |
| Crystal size/mm$^3$ | 0.5 × 0.4 × 0.1 | 0.5 × 0.25 × 0.25 | 0.5 × 0.2 × 0.2 |
| Radiation | Cu Kα (λ = 1.54184) | Cu (λ = 1.54187) | Cu Kα (λ = 1.54187) |
| 2Θ range for data collection/° | 6.4 to 136.48 | 7.5 to 136.44 | 6.62 to 136.5 |
| Index ranges | $-22 \le h \le 21$, $-5 \le k \le 4$, $-25 \le l \le 25$ | $-11 \le h \le 11$, $-13 \le k \le 13$, $-22 \le l \le 22$ | $-11 \le h \le 10$, $-17 \le k \le 17$, $-19 \le l \le 19$ |
| Reflections collected | 37795 | 21990 | 24007 |
| Independent reflections ($R_{int}$, $R_{sigma}$) | 2866 (0.0405, 0.0178) | 6800 (0.0814, 0.0686) | 7380 (0.0776, 0.0686) |
| Data/restraints/parameters | 2866/0/236 | 6800/0/463 | 7380/12/514 |
| Goodness-of-fit on $F^2$ | 1.039 | 1.037 | 1.036 |
| Final R indexes [I>=2σ (I)] ($R_1$,$wR_2$) | 0.0648, 0.1908 | 0.0531, 0.1392 | 0.0562, 0.1388 |
| Final R indexes [all data] ($R_1$,$wR_2$) | 0.0667, 0.1918 | 0.0549, 0.1407 | 0.0592, 0.1409 |
| Largest diff. peak/hole / e Å$^{-3}$ | 0.33/-0.31 | 1.61/-0.90 | 2.78/-1.66 |

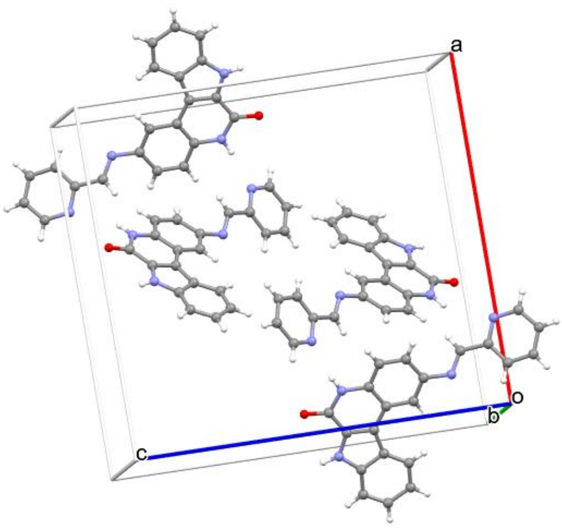


**Figure S20.** Unit cell of crystal IQPMA (**I**).

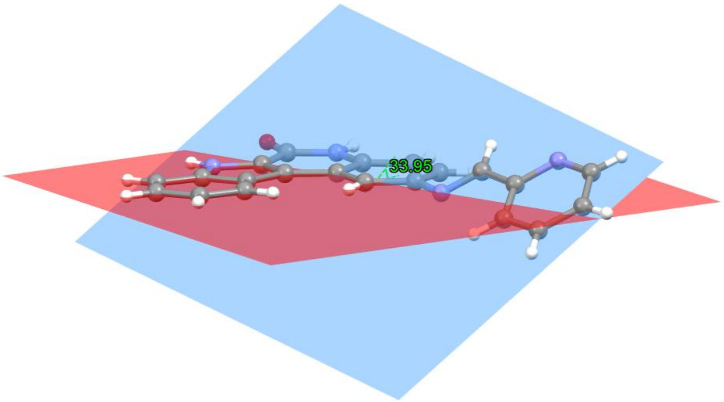


**Figure S21.** Conformation of IQPMA in crystal **I** showing the angle between the aromatic ring planes of 33.95(12)°.

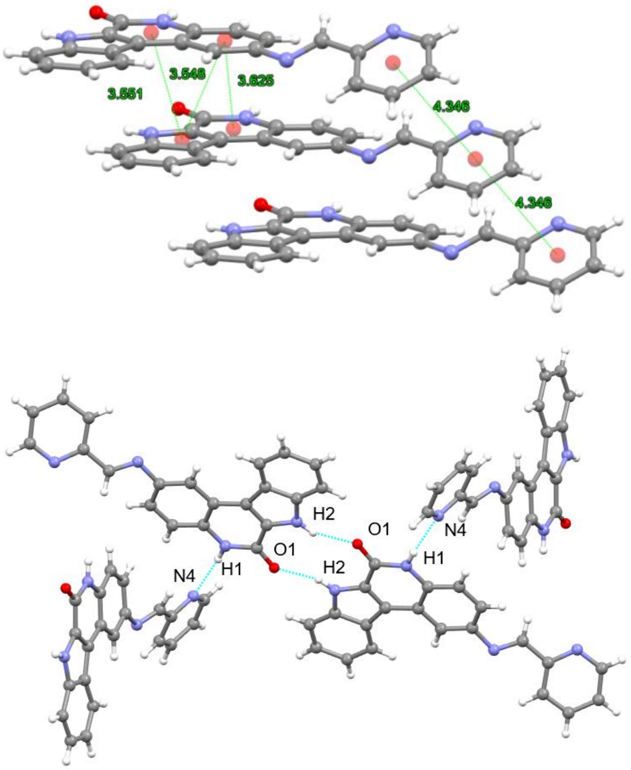


**Figure S22.** Packing arrangement in crystal IQPMA (**I)** showing the off-centered parallel stacking interactions with ring distances in units of Å (up) and the intermolecular H-bonds between neighboring molecules (bottom). The molecules are arranged into a herringbone structure and are held together in the crystal by off-centered parallel stacking interactions with interplanar separation of 3.300(1) Å. Two molecules are turned towards each other by an inversion center with O1 and N2 atoms and are connected by a symmetrical hydrogen bond interactions with the N2-H2...O1 bond.

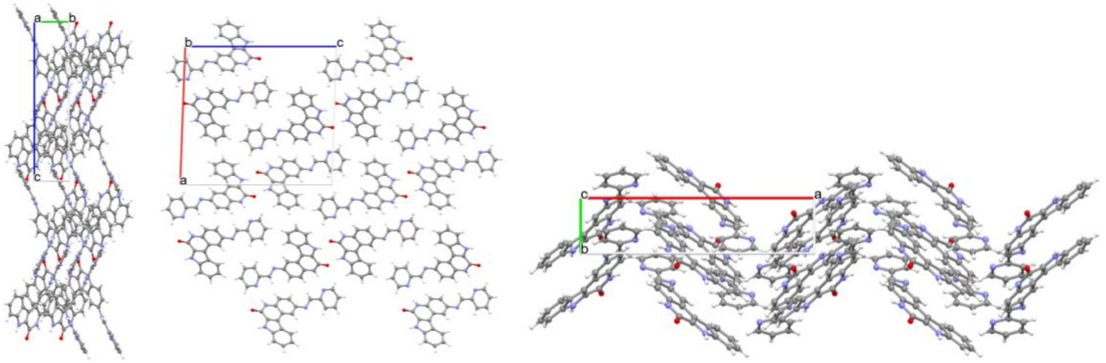


**Figure S23.** Packing arrangements in crystal IQPMA (**I**) viewed from the crystallographic directions ’a’, ’b’ and ’c’.

**Table S2.** Selected bond lengths and angles for IQPMA (**I**).

| Bond lengths | | | | | |
|---|---|---|---|---|---|
| **Atom** | **Atom** | **Length/Å** | **Atom** | **Atom** | **Length/Å** |
| O1 | C1 | 1.242(3) | C14 | C13 | 1.373(4) |
| N1 | C1 | 1.370(4) | C14 | C15 | 1.399(4) |
| N1 | C9 | 1.391(4) | C9 | C8 | 1.401(4) |
| N2 | C2 | 1.369(4) | C9 | C4 | 1.414(4) |
| N2 | C15 | 1.372(4) | N4 | C17 | 1.344(4) |
| N3 | C6 | 1.422(4) | N4 | C21 | 1.341(4) |
| N3 | C16 | 1.281(4) | C17 | C16 | 1.469(4) |
| C5 | C4 | 1.406(4) | C17 | C18 | 1.385(4) |
| C5 | C6 | 1.382(4) | C13 | C12 | 1.409(4) |
| C3 | C2 | 1.383(4) | C10 | C11 | 1.406(4) |
| C3 | C4 | 1.440(4) | C10 | C15 | 1.422(4) |
| C3 | C10 | 1.436(4) | C11 | C12 | 1.375(4) |
| C1 | C2 | 1.442(4) | C19 | C20 | 1.384(5) |
| C7 | C8 | 1.372(4) | C19 | C18 | 1.381(5) |
| C7 | C6 | 1.409(4) | C21 | C20 | 1.378(5) |

| Bond Angles | | | | | | | |
|---|---|---|---|---|---|---|---|
| **Atom** | **Atom** | **Atom** | **Angle/°** | **Atom** | **Atom** | **Atom** | **Angle/°** |
| C5 | C6 | N3 | 118.5(3) | C16 | C17 | N4 | 114.3(3) |
| C7 | C6 | N3 | 121.9(3) | C17 | C16 | N3 | 122.8(3) |
| C16 | N3 | C6 | 117.3(2) | | | | |

| Torsion Angles | | | | | | | | | |
|---|---|---|---|---|---|---|---|---|---|
| **A** | **B** | **C** | **D** | **Angle/°** | **A** | **B** | **C** | **D** | **Angle/°** |
| N3 | C16 | C17 | N4 | 174.1(3) | C7 | C6 | N3 | C16 | 37.4(3) |
| N3 | C16 | C17 | C18 | -4.6(4) | C6 | N3 | C16 | C17 | -172.0(3) |
| C5 | C6 | N3 | C16 | -147.7(3) | C2 | N2 | C15 | C10 | 0.6(3) |

**Table S3.** Crystal data and structure refinement for crystals **3b**, **4b** and **6b**.

| Identification code | [RhCp*(IQPMA)Cl]$PF_6$×DMF (**3b**×DMF) | [RuCym(DIPMA)Cl]$PF_6$ (**4b**) | [RhCp*(DIPMA)Cl]$PF_6$ (**6b**) |
|---|---|---|---|
| CCDC number | 2529819 | 2529820 | 2529821 |
| Empirical formula | $C_{65}H_{65}Cl_2F_{12}N_9O_3P_2Rh_2$ | $C_{28}H_{36}ClF_6N_2PRu$ | $C_{28}H_{37}ClF_6N_2PRh$ |
| Moiety formula | 2($C_{31}H_{29}ClN_4ORh$), 2($PF_6$), $C_3H_7NO$ | $C_{28}H_{36}ClN_2Ru$, $PF_6$ | $C_{28}H_{37}ClN_2Rh$, $PF_6$ |
| Formula weight | 1586.944 | 682.093 | 684.92 |
| Temperature/K | 100.0(5) | 113(2) | 293(2) |
| Crystal system | monoclinic | monoclinic | orthorhombic |
| Space group | $P2_1/n$ | $P2_1/n$ | *Pbca* |
| a/Å | 7.4408(2) | 17.3124(4) | 13.8225(3) |
| b/Å | 28.614(2) | 10.0592(2) | 19.9152(4) |
| c/Å | 15.9388(4) | 17.6150(4) | 22.1679(4) |
| α/° | 90 | 90 | 90 |
| β/° | 101.226(2) | 108.506(8) | 90 |
| γ/° | 90 | 90 | 90 |
| Volume/$Å^3$ | 3328.6(3) | 2909.01(18) | 6102.3(2) |
| Z/Z' | 2/1 | 4/1 | 8/1 |
| $\rho_{calc}$ / $g/cm^3$ | 1.583 | 1.557 | 1.491 |
| $\mu/mm^{-1}$ | 5.959 | 6.262 | 6.332 |
| F(000) | 1617.0 | 1399.8 | 2816.1 |
| Crystal size/$mm^3$ | 0.15 × 0.05 × 0.02 | 0.2 × 0.2 × 0.1 | 0.3 × 0.3 × 0.2 |
| Radiation | Cu Kα (λ = 1.54184) | Cu Kα (λ = 1.54187) | CuKα (λ = 1.54178) |
| 2Θ range for data collection/° | 6.18 to 149.22 | 6.24 to 136.48 | 7.98 to 136.444 |
| Index ranges | -9 ≤ h ≤ 9, -35 ≤ k ≤ 35, -19 ≤ l ≤ 19 | -20 ≤ h ≤ 20, -12 ≤ k ≤ 12, -21 ≤ l ≤ 20 | -16 ≤ h ≤ 15, -23 ≤ k ≤ 23, -26 ≤ l ≤ 26 |
| Reflections collected | 67569 | 30740 | 66463 |
| Independent reflections ($R_{int}$, $R_{sigma}$) | 6800 (0.0522, 0.0220) | 5313 (0.0680, 0.0599) | 5564 (0.0718, 0.0430) |
| Data/restraints/parameters | 6800/31/459 | 5313/0/359 | 5564/0/361 |
| Goodness-of-fit on $F^2$ | 1.037 | 1.061 | 1.081 |
| Final R indexes [I>=2σ (I)] ($R_1$,$wR_2$) | 0.0505, 0.1316 | 0.0550, 0.1298 | 0.0722, 0.1559 |
| Final R indexes [all data] ($R_1$,$wR_2$) | 0.0517, 0.1325 | 0.0673, 0.1358 | 0.0895, 0.1649 |
| Largest diff. peak/hole / e $Å^{-3}$ | 1.65/-0.88 | 1.19/-0.83 | 1.04/-1.21 |

**Table S4.** Selected bond length and angles together with ring plane angles in crystals **1b-4b**, **6b** and related crystals used in the statistical investigation.[a]

| | Distance (Å) | | | | Angle (°) | | | | | | Ring plane angle (°) | | |
|---|---|---|---|---|---|---|---|---|---|---|---|---|---|
| | M-Cl | M-N | M-$N_{pyr}$ | M-Cg(A) | N-M-Cl | N-M-$N_{pyr}$ | $N_{pyr}$-M-Cl | Cg(A)-M-Cl | Cg(A)-M-N | Cg(A)-M-$N_{pyr}$ | A – BC | A – D | D - BC |
| RuCym(IQPMA) (**1b**) | 2.390 | 2.091 | 2.076 | 1.681 | 87.2 | 77.0 | 85.6 | 128.7 | 131.2 | 129.6 | 52.0 | 12.0 | 63.9 |
| OsCym(IQPMA) (**2b**) | 2.391 | 2.085 | 2.088 | 1.683 | 84.3 | 75.0 | 82.7 | 128.4 | 132.8 | 133.8 | 64.9 | 9.4 | 65.1 |
| RhCp*(IQPMA) (**3b**) | 2.386 | 2.118 | 2.124 | 1.785 | 89.3 | 76.4 | 91.1 | 124.3 | 130.8 | 129.9 | 49.4 | 5.0 | 45.1 |
| RuCym(DIPMA) (**4b**) | 2.382 | 2.108 | 2.075 | 1.696 | 89.4 | 76.9 | 80.3 | 124.8 | 132.4 | 134.8 | 65.5 | 21.5 | 86.6 |
| RuCym(DIPMA) [ROLDIJ] | 2.397 | 2.099 | 2.090 | 1.693 | 87.9 | 77.1 | 79.6 | 126.2 | 131.6 | 135.6 | 69.4 | 10.4 | 78.5 |
| OsCym(DIPMA) [TELBAQ] | 2.392 | 2.102 | 2.087 | 1.701 | 88.7 | 76.0 | 79.9 | 125.2 | 133.0 | 135.2 | 65.2 | 21.5 | 86.2 |
| RhCp*(DIPMA) (**6b**) | 2.401 | 2.191 | 2.095 | 1.794 | 93.0 | 76.8 | 83.8 | 125.2 | 130.6 | 131.1 | 58.3 | 4.3 | 62.6 |
| RhCp*(DIPMA) [ACUDAH] | 2.408 | 2.142 | 2.114 | 1.805 | 90.7 | 76.6 | 81.4 | 121.8 | 134.0 | 134.8 | 72.5 | 11.5 | 79.7 |
| IrCp*(DIPMA) [DOSXAN] | 2.398 | 2.103 | 2.104 | 1.804 | 88.1 | 76.2 | 81.1 | 124.6 | 134.0 | 134.2 | 69.6 | 6.8 | 71.6 |
| IrCp*(DIPMA) [LIYSAQ] | 2.410 | 2.129 | 2.099 | 1.800 | 92.7 | 76.3 | 81.4 | 123.8 | 132.4 | 133.0 | 62.1 | 6.8 | 68.9 |
| IrCp*(DIPMA) [SEPWUI] | 2.399 | 2.108 | 2.094 | 1.812 | 89.4 | 76.4 | 80.3 | 121.9 | 135.2 | 135.1 | 73.5 | 11.8 | 81.2 |
| RuCym(PMA) [DEQNAS] | 2.399 | 2.100 | 2.085 | 1.690 | 86.5 | 76.7 | 86.2 | 127.2 | 132.3 | 130.5 | 54.0 | 10.2 | 48.2 |
| RhCp*(PMA) [AQECOQ] | 2.393 | 2.119 | 2.106 | 1.789 | 87.7 | 76.6 | 85.9 | 125.7 | 132.8 | 131.0 | 57.7 | 6.9 | 51.6 |
| IrCp*(PMA) [AQECIK] | 2.400 | 2.108 | 2.096 | 1.803 | 86.7 | 76.2 | 84.8 | 126.1 | 133.3 | 131.8 | 58.9 | 7.4 | 52.3 |

[a]Cg(A) refers to the centre-of-gravity of Cp* or Cym ring. A, BC and D refer to the ring planes (see Figure S34). The largest SD of distance data is ±0.005 Å and angle data is ±1°.

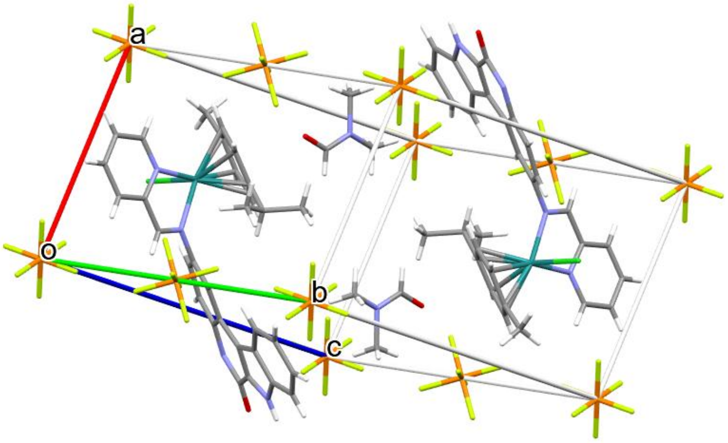


**1b**

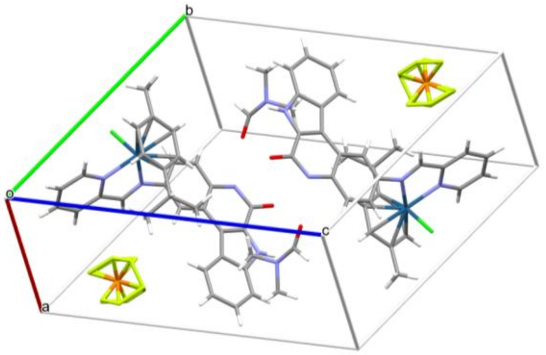


**2b**

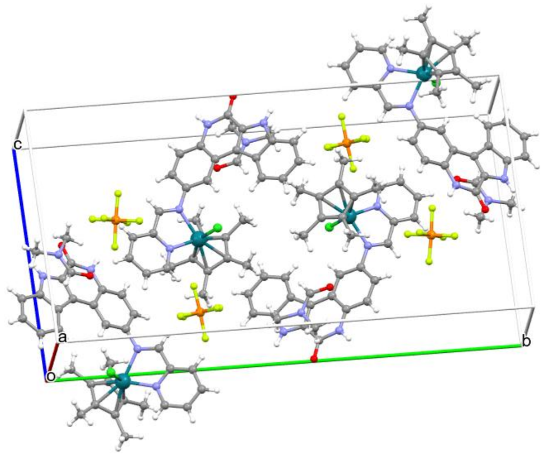


**3b**

**Figure S24.** Unit cell of [RuCym(IQPMA)Cl]$PF_6$×DMF (+solvent) (**1b**), [OsCym(IQPMA)Cl]$PF_6$×DMF (+solvent) (**2b**) and [RhCp*(IQPMA)Cl]$PF_6$×DMF (**3b**).

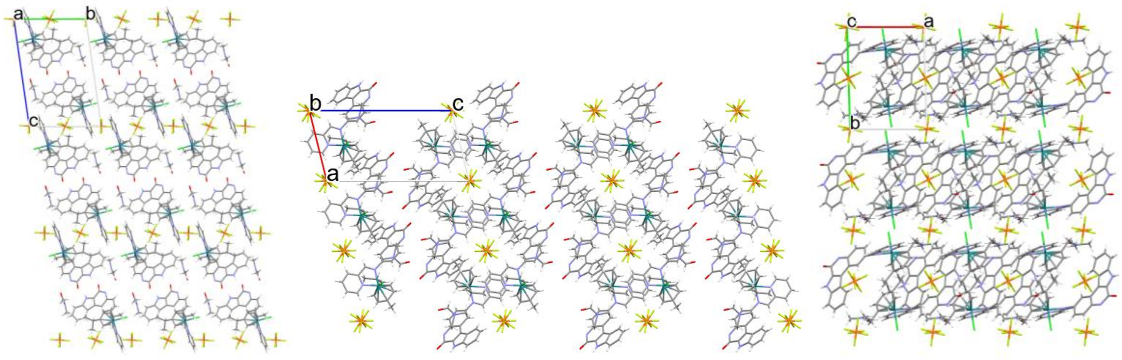

**1b**

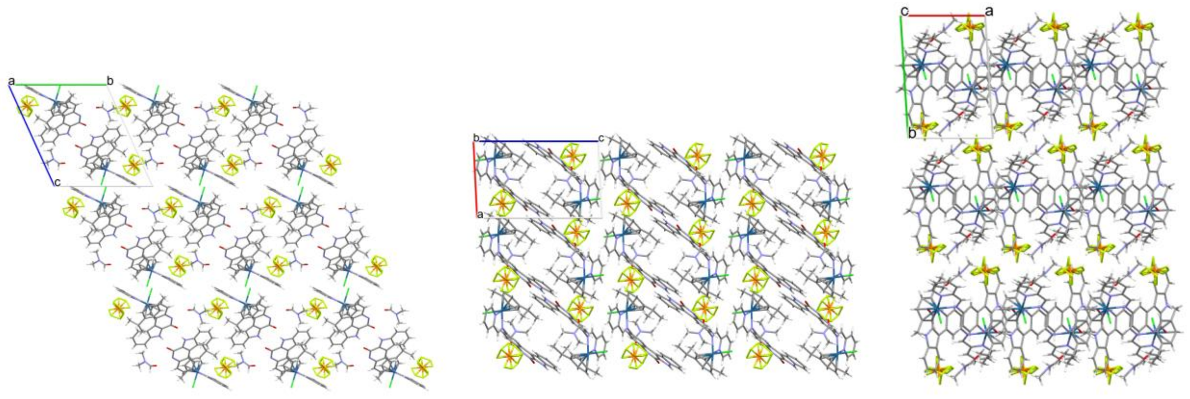

**2b**

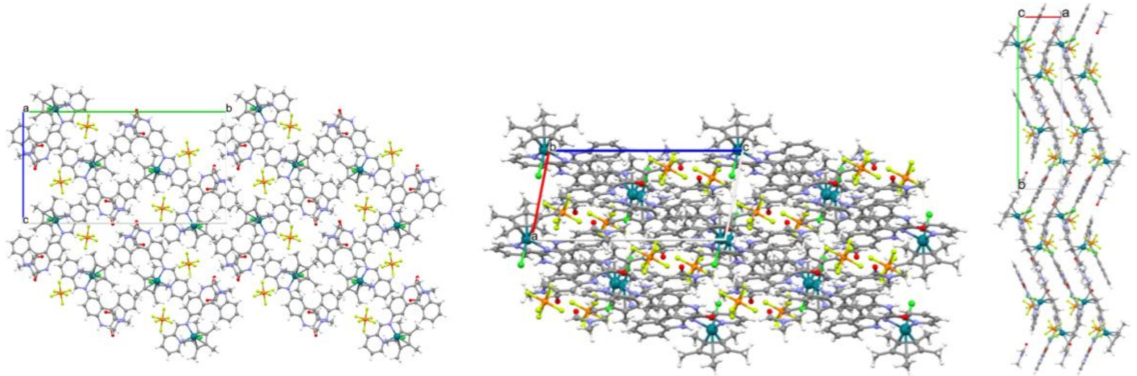

**3b**

**Figure S25.** Packing arrangements in crystal [RuCym(IQPMA)Cl]$PF_6$×DMF (+solvent) (**1b**), [OsCym(IQPMA)Cl]$PF_6$×DMF (+solvent) (**2b**) and [RhCp*(IQPMA)Cl]$PF_6$×DMF (**3b**) viewed from the crystallographic directions ’a’, ’b’ and ’c’.

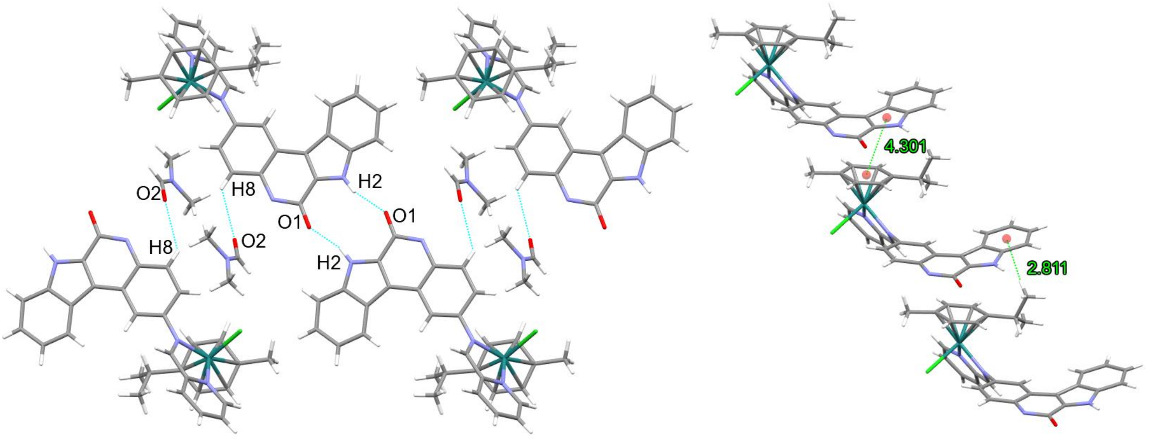


**1b**

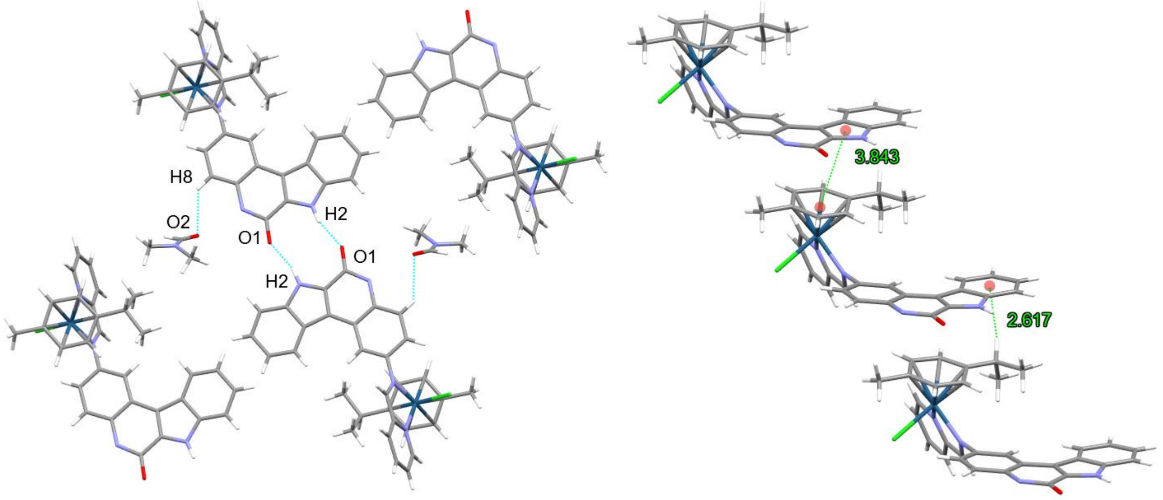


**2b**

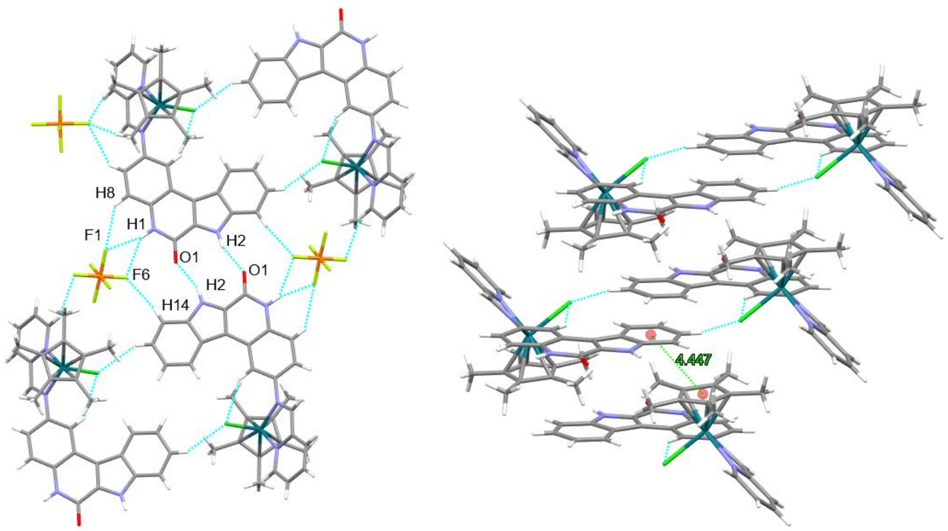


**3b**

**Figure S26.** Packing arrangements in crystal [RuCym(IQPMA)Cl]$PF_6$×DMF (+solvent) (**1b**), [OsCym(IQPMA)Cl]$PF_6$×DMF (+solvent) (**2b**) and [RhCp*(IQPMA)Cl]$PF_6$×DMF (**3b**) showing the intermolecular H-bonds between neighbouring molecules (left) and off-centered parallel stacking arrangements (right).

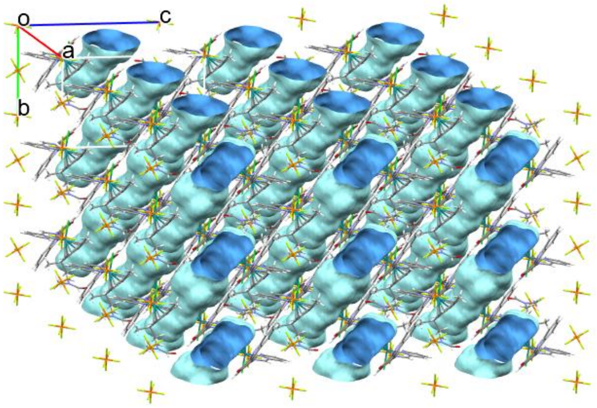


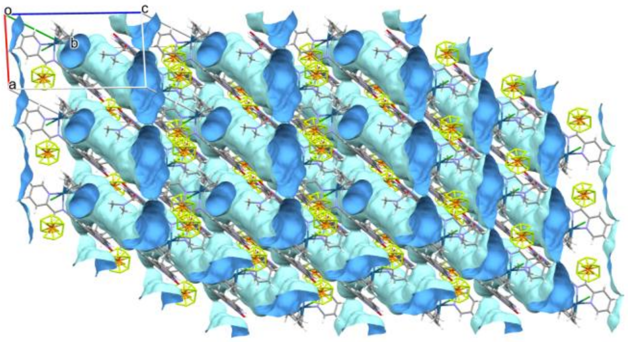


**Figure S27**. Channels are shown in blue in crystals [RuCym(IQPMA)Cl]$PF_6$×DMF (+solvent) (**1b**) with 16.9% of unit cell volume (328.3 Å$^3$) (up) and in crystal [OsCym(IQPMA)Cl]$PF_6$×DMF (+solvent) (**2b**) with 21.8% of unit cell volume (448.2 Å$^3$) (bottom). The DMF molecule is located in two possible positions, with occupancy 0.5. The channels contain disordered diethyl ether solvent. Crystals **1b** and **2b** contain a significant amount of crystal solvent located in cavities and channels within the crystal. These channels account for 16.9% and 21.8% of the volume of the unit cell in case of **1b** and **2b**, respectively

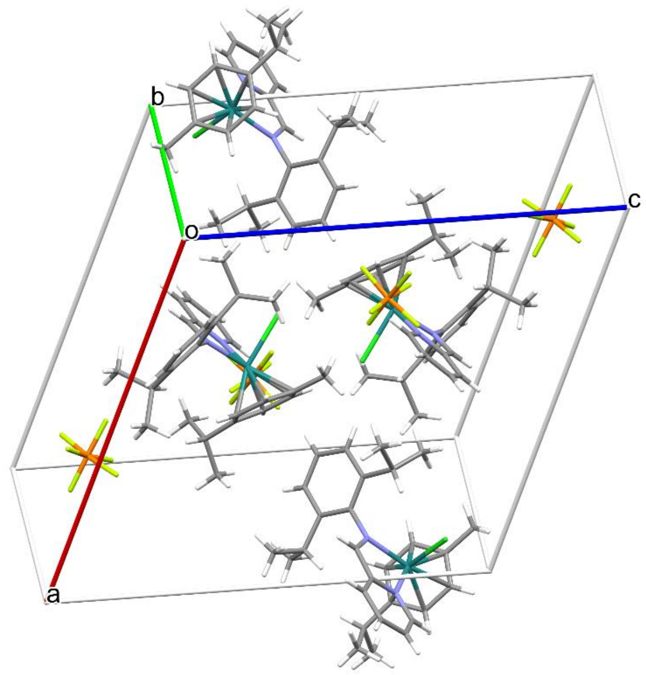


**4b**

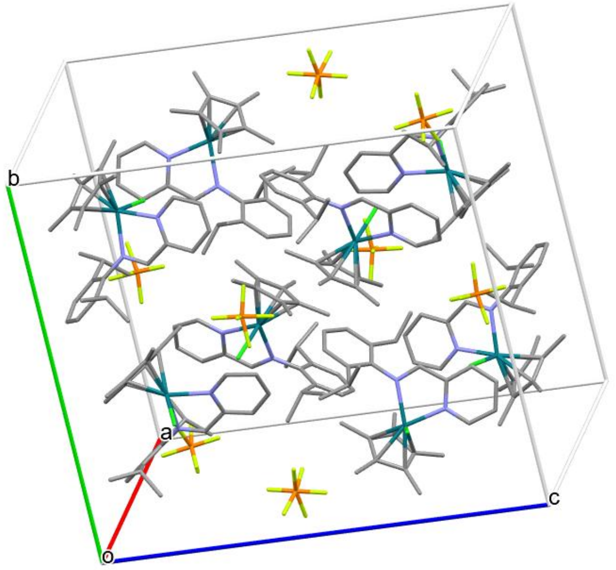


**6b**

**Figure S28.** Unit cell containing eight complexes in the crystal of [RuCym(DIPMA)Cl]$PF_6$ (**4b**) and RhCp*(DIPMA)Cl]$PF_6$ (**6b**).

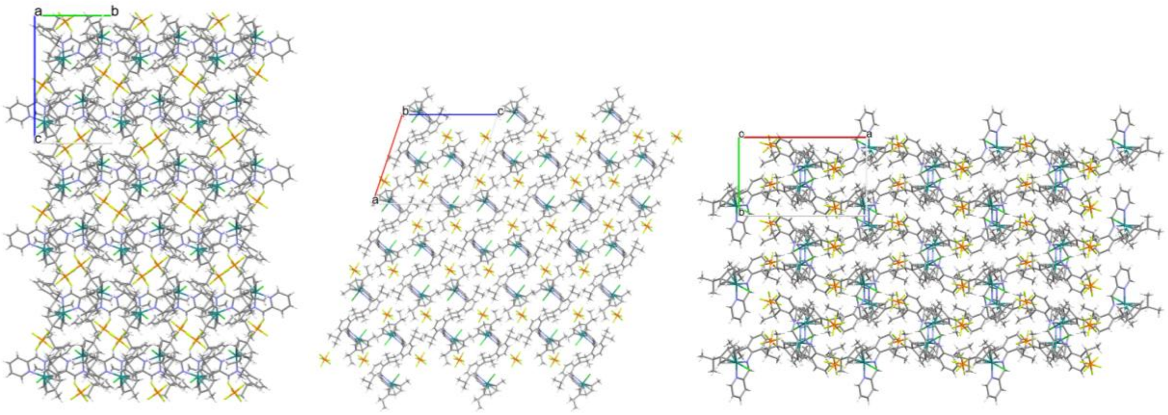

**4b**

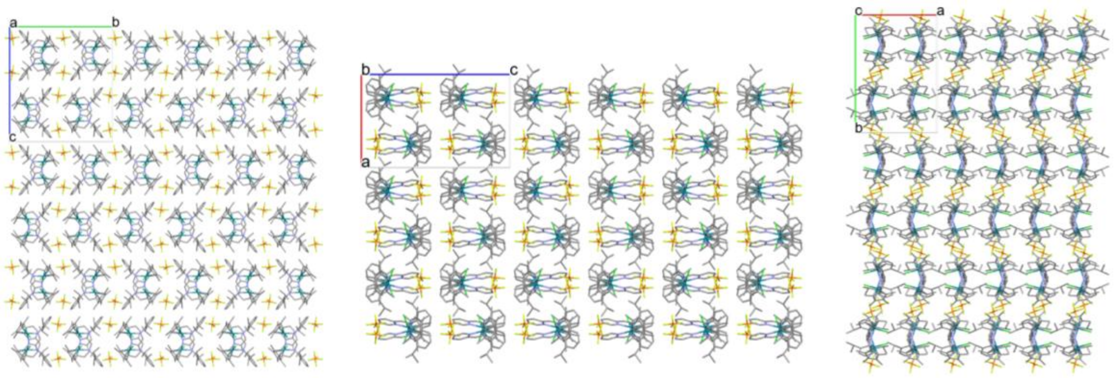

**6b**

**Figure S29.** Packing arrangements in crystals [RuCym(DIPMA)Cl]$PF_6$ (**4b**) and RhCp*(DIPMA)Cl]$PF_6$ (**6b**) viewed from the crystallographic directions ’a’, ’b’ and ’c’.

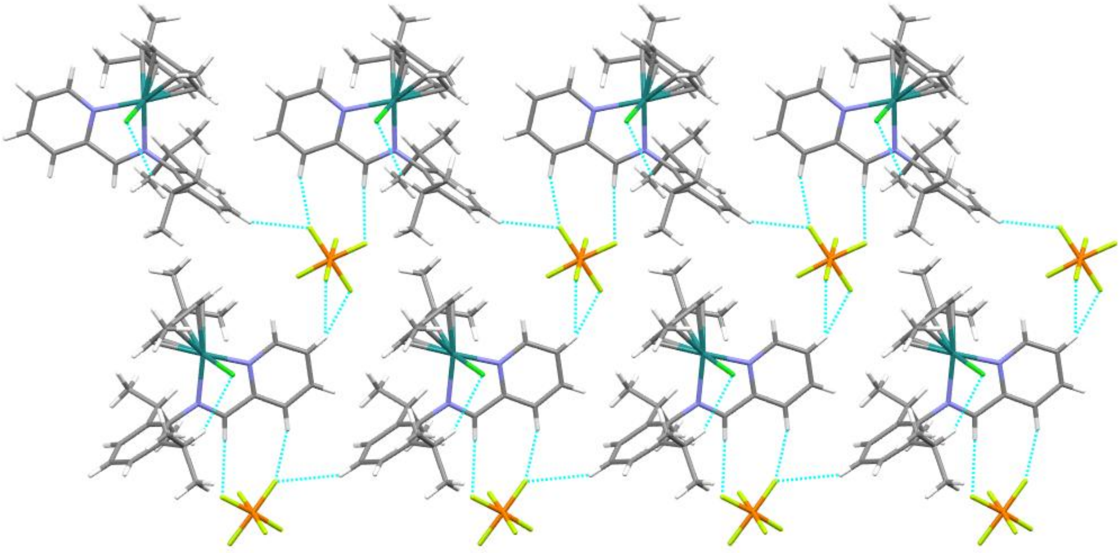

**4b**

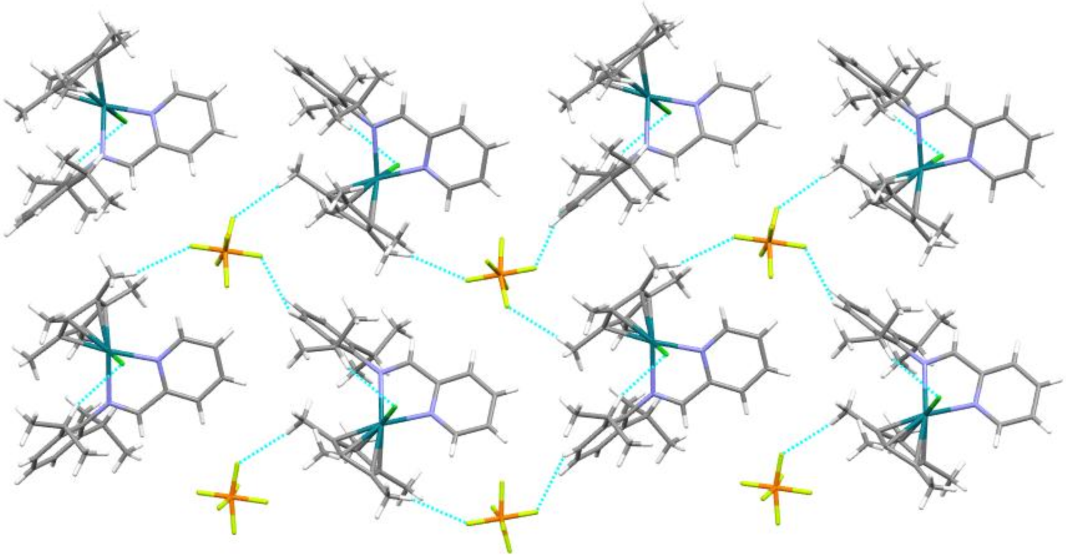

**6b**

**Figure S30.** Packing arrangements in crystals [RuCym(DIPMA)Cl]$PF_6$ (**4b**) and [RhCp*(DIPMA)Cl]$PF_6$ (**6b**) showing main H-bond interactions between neighboring complexes and the $PF_6^-$ counter ions.

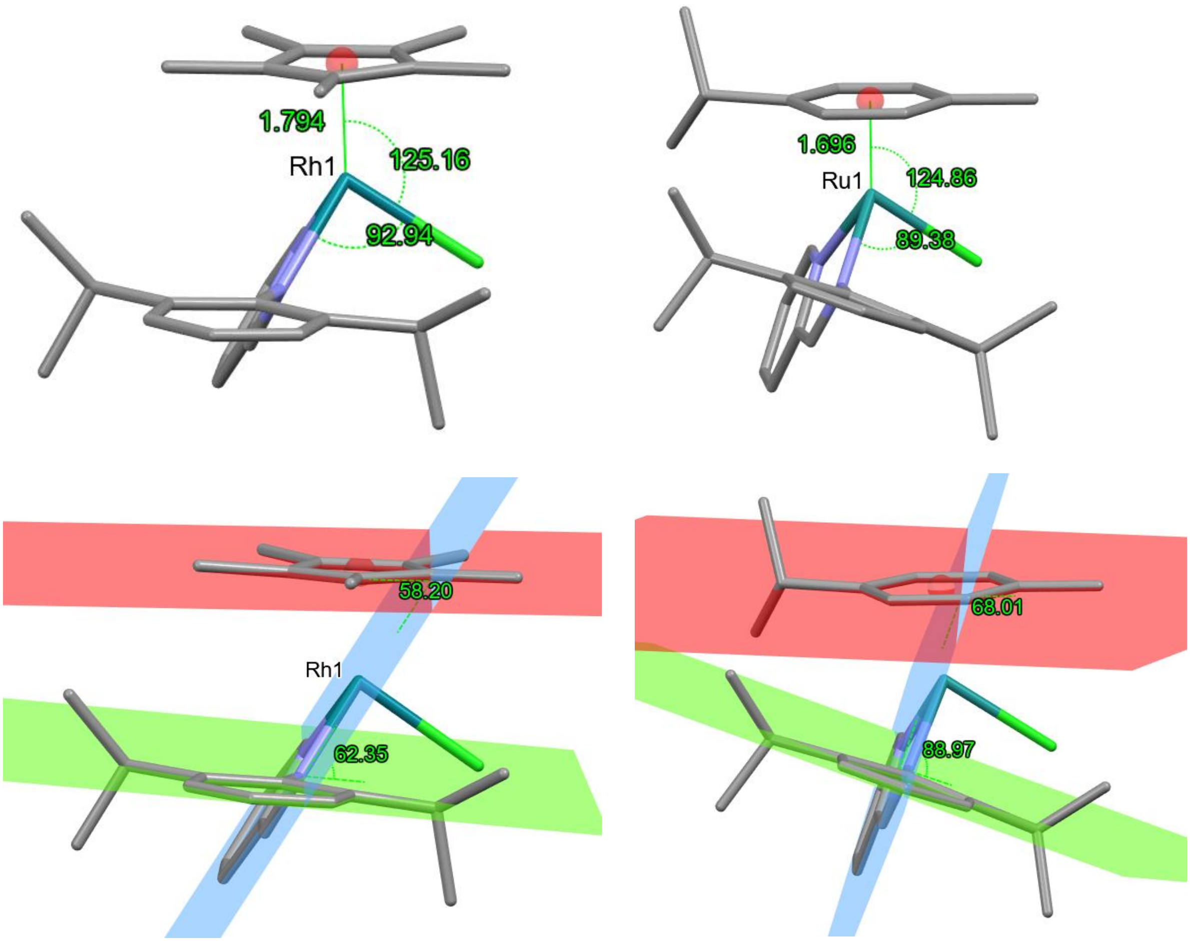


**Figure S31.** Comparison of the coordination sphere in crystal [RuCym(DIPMA)Cl]$PF_6$ (**4b**) (right) and [RhCp*(DIPMA)Cl]$PF_6$ (**6b**) (left). Angles between ring planes are shown (ring A (red), ring BC (blue) and ring D (green)).The figure shows that there are significant differences between the angles enclosed by the Cp*/*p*-cymene and coordinating pyridine rings and the planes of the pyridine and non-coordinating aromatic rings. This is also shown in Figure 4, where the metal ion and the associated $N_{pyridine}$, N, and Cl atoms are aligned for complexes of IQPMA and DIPMA.

**Table S5.** Investigated complexes for structural comparison.

| Complex | Ref. Code | reference |
|---|---|---|
| [RuCym(IQPMA)Cl]$PF_6$×DMF (+solvent) | **1b** | This work |
| [OsCym(IQPMA)Cl]$PF_6$×DMF (+solvent) | **2b** | This work |
| [RhCp*(IQPMA)Cl]$PF_6$×DMF | **3b** | This work |
| [RuCym(DIPMA)Cl]$PF_6$ | **4b** | This work |
| [RhCp*(DIPMA)Cl]$PF_6$ | **6b** | This work |
| [IrCp*(DIPMA)Cl]$PF_6$×toluene | [DOSXAN] | [SI-7] |
| [IrCp*(DIPMA)Cl]4-methylbenzene-1-sulfonate | [LIYSAQ] | [SI-8] |
| [IrCp*(DIPMA)Cl] $PF_6$×$CH_2Cl_2$ | [SEPWUI] | [SI-9] |
| [RuCym(DIPMA)Cl]$BF_4$ | [ROLDIJ] | [SI-10] |
| [OsCym(DIPMA)Cl]$PF_6$ | [TELBAQ] | [SI-11] |
| [RhCp*(DIPMA)Cl]$PF_6$× $CH_2Cl_2$ | [ACUDAH] | [SI-12] |
| [RuCym(PMA)Cl]$PF_6$×acetone | [DEQNAS] | [SI-13] |
| [RhCp*(PMA)Cl]$PF_6$ | [AQECOQ] | [SI-14] |
| [IrCp*(PMA)Cl] $PF_6$ | [AQECIK] | [SI-15] |

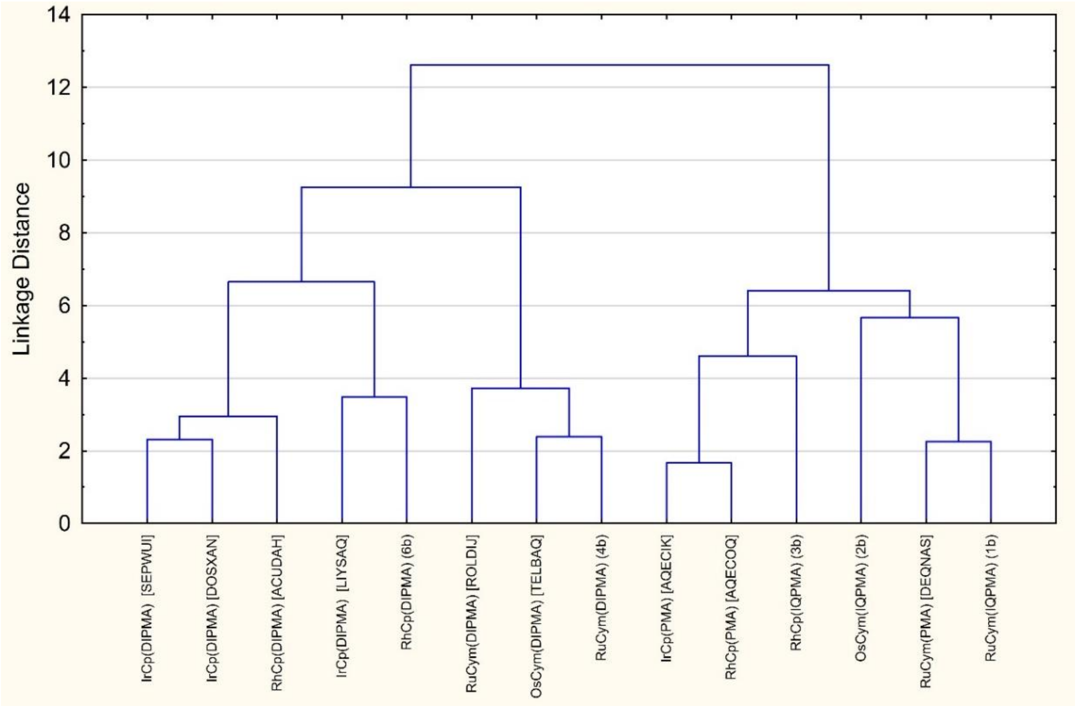


**Figure S32.** Tree diagram calculated by cluster analysis for 14 cases obtained for data in Table S5 by Ward's method and Euclidean distances. The parameters that are close in linkage distance are correlated with each other.

**Table S6.** Correlation data (Pearson's values (R)), between the variables of structural data collected in Table S4. Correlations marked with yellow are significant with $R > 0.70$ at $p < 0.05$.

| Variable | **Means** | **Std.Dev.** | **M-Cl** | **M-N** | **M-$N_{pyr}$** | **M-Cg(A)** | **N-M-Cl** | **N-M-$N_{pyr}$** | **$N_{pyr}$-M-Cl** | **Cg(A)-M-Cl** | **Cg(A)-M-N** | **Cg(A)-M-$N_{pyr}$** | **A – BC** | **A – D** | **D - BC** |
|---|---|---|---|---|---|---|---|---|---|---|---|---|---|---|---|
| **M-Cl** | 2.3962 | 0.00785 | 1.0000 | 0.4372 | 0.2809 | 0.5386 | 0.4459 | 0.0371 | -0.2765 | -0.4090 | 0.3226 | 0.1337 | 0.3271 | -0.4077 | 0.0391 |
| **M-N** | 2.1145 | 0.02653 | 0.4372 | 1.0000 | 0.3684 | 0.5579 | 0.8115 | 0.3065 | 0.0642 | -0.4452 | -0.2487 | -0.1648 | -0.0422 | -0.3767 | -0.0627 |
| **M-$N_{pyr}$** | 2.0952 | 0.01364 | 0.2809 | 0.3684 | 1.0000 | 0.7302 | 0.2978 | -0.1065 | 0.4026 | -0.5525 | 0.0938 | -0.1349 | -0.0339 | -0.5931 | -0.3553 |
| **M-Cg(A)** | 1.7526 | 0.05624 | 0.5386 | 0.5579 | 0.7302 | 1.0000 | 0.5378 | 0.0063 | 0.0886 | -0.7207 | 0.3759 | -0.0347 | 0.1742 | -0.5541 | -0.1562 |
| **N-M-Cl** | 88.6686 | 2.34021 | 0.4459 | 0.8115 | 0.2978 | 0.5378 | 1.0000 | 0.4054 | -0.1745 | -0.6728 | -0.1344 | 0.0749 | 0.1229 | -0.1170 | 0.2577 |
| **N-M-$N_{pyr}$** | 76.4336 | 0.51795 | 0.0371 | 0.3065 | -0.1065 | 0.0063 | 0.4054 | 1.0000 | 0.0517 | -0.1355 | -0.3434 | -0.1959 | -0.1560 | 0.0477 | 0.0443 |
| **$N_{pyr}$-M-Cl** | 83.1400 | 3.27076 | -0.2765 | 0.0642 | 0.4026 | 0.0886 | -0.1745 | 0.0517 | 1.0000 | 0.2934 | -0.5090 | -0.8973 | -0.8783 | -0.5229 | -0.9041 |
| **Cg(A)-M-Cl** | 125.2821 | 2.05074 | -0.4090 | -0.4452 | -0.5525 | -0.7207 | -0.6728 | -0.1355 | 0.2934 | 1.0000 | -0.5088 | -0.4385 | -0.5333 | -0.0216 | -0.3921 |
| **Cg(A)-M-N** | 132.5950 | 1.29982 | 0.3226 | -0.2487 | 0.0938 | 0.3759 | -0.1344 | -0.3434 | -0.5090 | -0.5088 | 1.0000 | 0.5839 | 0.7241 | 0.2212 | 0.4103 |
| **Cg(A)-M-$N_{pyr}$** | 132.8829 | 2.15951 | 0.1337 | -0.1648 | -0.1349 | -0.0347 | 0.0749 | -0.1959 | -0.8973 | -0.4385 | 0.5839 | 1.0000 | 0.9357 | 0.5242 | 0.8733 |
| **A – BC** | 62.3443 | 7.59280 | 0.3271 | -0.0422 | -0.0339 | 0.1742 | 0.1229 | -0.1560 | -0.8783 | -0.5333 | 0.7241 | 0.9357 | 1.0000 | 0.3303 | 0.8086 |
| **A – D** | 10.3921 | 5.32100 | -0.4077 | -0.3767 | -0.5931 | -0.5541 | -0.1170 | 0.0477 | -0.5229 | -0.0216 | 0.2212 | 0.5242 | 0.3303 | 1.0000 | 0.7038 |
| **D – BC** | 67.2479 | 14.10955 | 0.0391 | -0.0627 | -0.3553 | -0.1562 | 0.2577 | 0.0443 | -0.9041 | -0.3921 | 0.4103 | 0.8733 | 0.8086 | 0.7038 | 1.0000 |

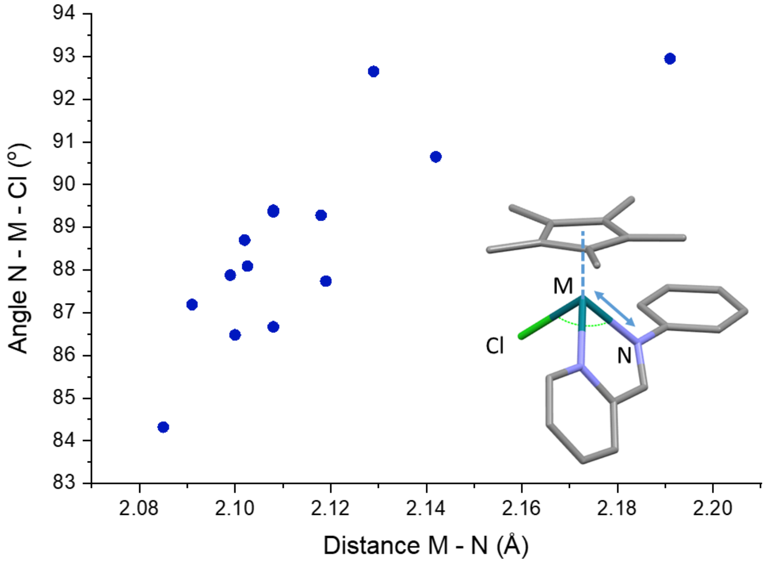


**Figure S33.** Correlation diagram of angle N−M−Cl versus distance M-N for 14 organometallic complexes based on their crystallographic data. R = 0.8115. Considering the intra- and intermolecular interactions of Cl, we cannot find a clear reason for this effect, so the different electron distribution caused by the D−BC angle may be one of the reasons. The same effect may also occur in that the M−N bond distance correlates positively with the N−M−Cl angle.

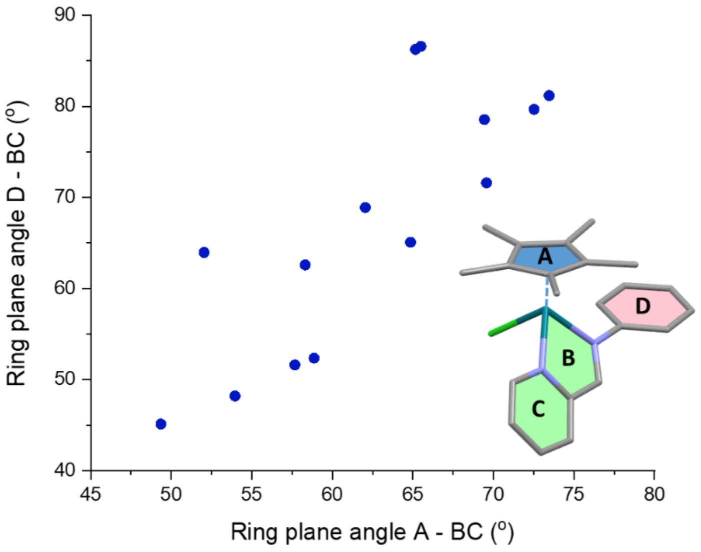


**Figure S34.** Correlation diagram between ring plane angles D-BC versus A-BC for 14 organometallic complexes based on their crystallographic data. (R = 0.8086)

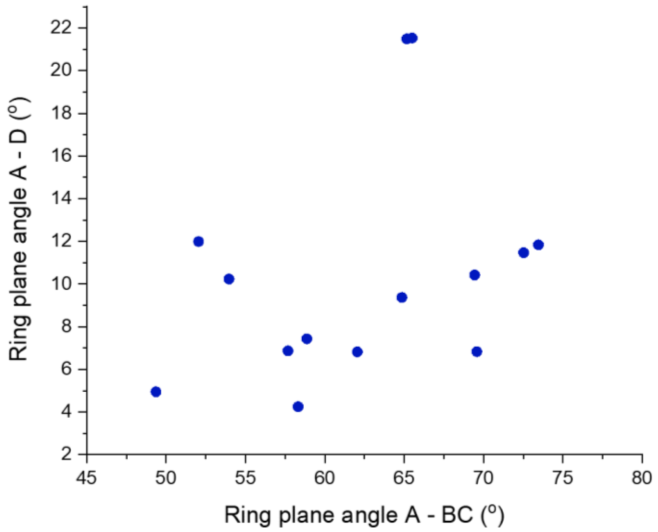


**Figure S35.** Correlation diagram between ring plane angles A-D versus A-BC for 14 organometallic complexes based on their crystallographic data. Crystal [RuCym(DIPMA)Cl]$PF_6$ (**4b**) and [OsCym(DIPMA)Cl]$PF_6$ [TELBAQ] are exceptions, where ring plane angles A-D are 21.5 degrees. The steric hindrance is most likely between rings A and D, since angles A−BC and D−BC correlate (R = 0.8086) (Figure S34) in such a way that rings A−D strive to remain parallel in every complex (angle < 12 degrees).

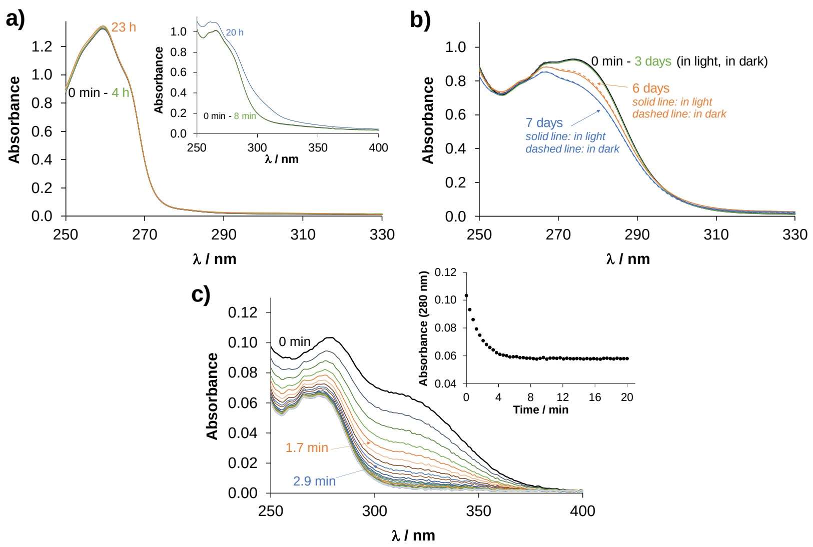


**Figure S36.** UV-vis spectra of PMA a) at pH = 0.7 with the inserted spectra at pH 13.0, and b) at pH = 7.4 (PBS' buffer) followed over time in pure aqueous solutions. c) The UV-vis spectra of PMA at pH 7.4 in 1% (*v*/*v*) DMSO/$H_2O$ diluted from a pure DMSO stock solution (1 mM) with PBS buffer (pH 7.4). Spectra revealed that PMA is not light-sensitive and remains stable for 23 h at pH 0.7 and for 3 days at pH 7.4 in aqueous solutions, whereas under basic conditions the spectra were unchanged for only *ca.* 8 min. {$c_{PMA}$ = 200 µM for figures a) and b); ~15 µM for c); $\ell$ = 1 cm (UV-vis); $T$ = 25.0 °C}

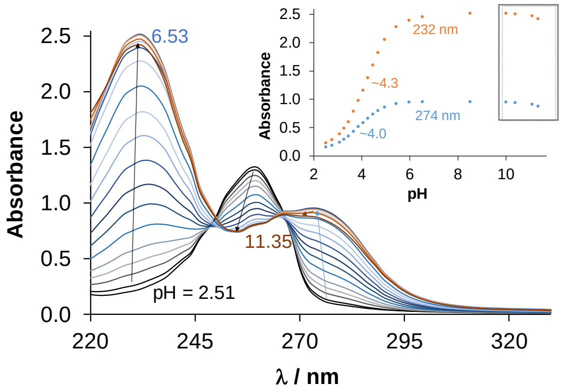


**Figure S37.** UV-vis spectra of PMA recorded at different pH values. The unusual spectral changes at pH > ~10 are most probably due to hydrolysis as in this pH range no proton dissociation process is expected. Inserted figure shows the absorbance values at 232 nm and 274 nm plotted against the pH with the indication of their different inflection points (pH 4.0 and 4.3) due to the co-existence of Z and E isomers. The grey frame indicates the pH range in which hydrolysis occurs. Spectral changes at pH > ~10 are attributed to the ligand hydrolysis. {$c_{PMA}$ = 200 µM; $\ell$ = 1 cm (UV-vis); $I$ = 0.10 M (KCl); $T$ = 25.0 °C}

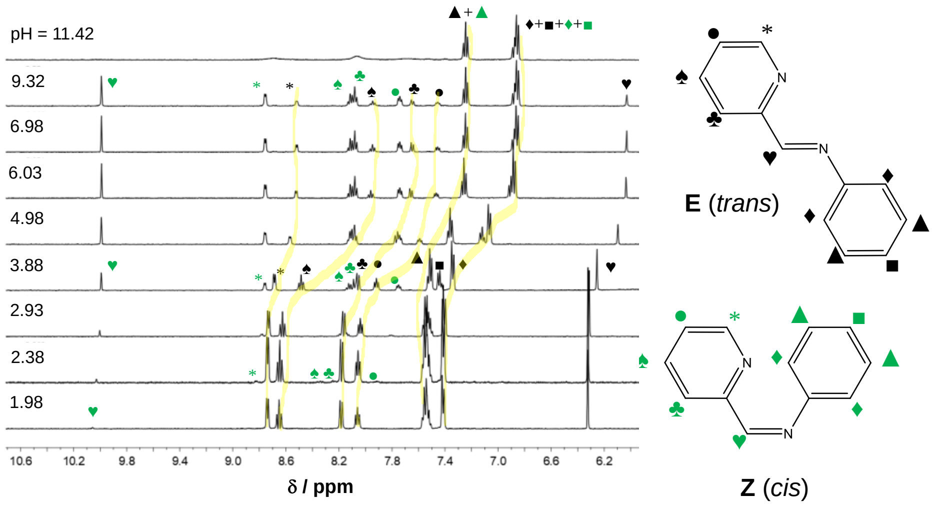


**Figure S38.** $^{1}$H NMR spectra of PMA at different pH values, the structure of the *Z* and *E* isomers in their neutral forms and the symbols for peak assignations. At pH 11.42, a dynamic equilibrium is established due to the hydrolysis of the Schiff base (PMA), yielding picolinaldehyde and aniline. While the resonances attributed to the benzene-ring CH protons appear as distinct peaks, as these nuclei are less sensitive to the ongoing hydrolysis, the pyridine CH and imine (CH=N) signals are broadened or not visible, owing to fast exchange processes between PMA and its hydrolysis product, picolinaldehyde. {$c_{PMA}$ = 730 μM; 10% (v/v) $D_2O/H_2O$; $I$ = 0.10 M (KCl); $T$ = 25.0 $^{o}$C}

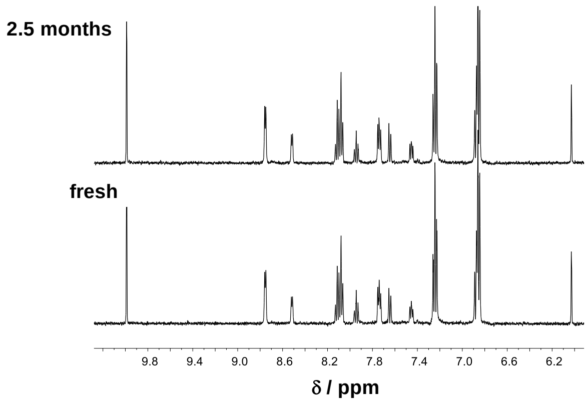


**Figure S39.** $^{1}$H NMR spectra of the stock solution of PMA: freshly prepared and after 2.5 months; peak assignations can be found in Figure S38. The spectrum shows the presence of the two isomeric forms, with no detectable changes. Notably, some spectral changes were seen by UV-vis spectroscopy using 200 μM concentration at pH 7.4 after 3 days (Figure S36b). {$c_{PMA}$ = 3 mM; pH ~8; 10% (v/v) $D_2O/H_2O$; $I$ = 0.10 M (KCl); $T$ = 25.0 $^{o}$C}

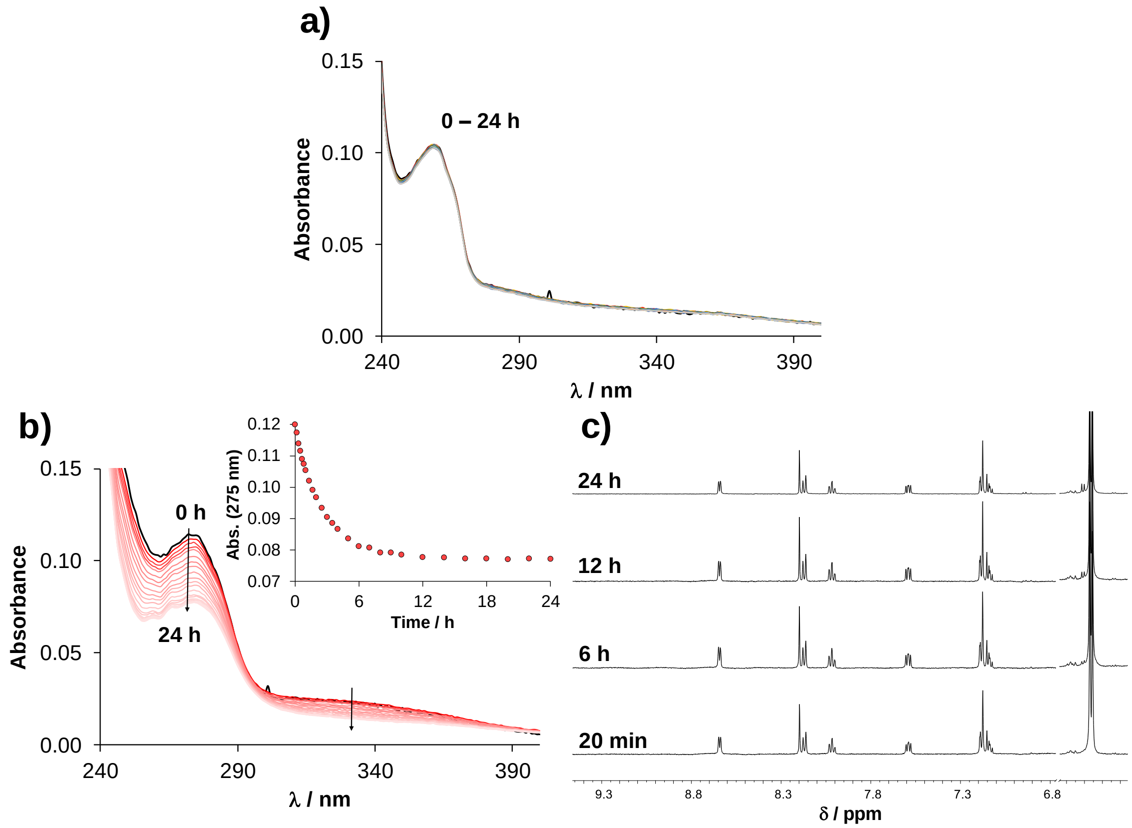


**Figure S40.** UV-vis spectra of DIPMA in DMSO/$H_2O$ solvent mixtures a) in 0.1 M HCl (pH = 1.0) with 1% (v/v) DMSO, and b) in PBS' buffer with 1% (v/v) DMSO (pH = 7.4), followed over time. Precipitate formation was also observed. c) $^1$H NMR spectra of DIPMA in 50% (v/v) DMSO-$d_6$/PBS' (pH = 7.4) followed over time showing no changes under the conditions used. {$c_{DIPMA}$ = 10 μM (UV-vis) and 300 μM ($^1$H NMR); ℓ = 1 cm (UV-vis), T = 25.0 °C}

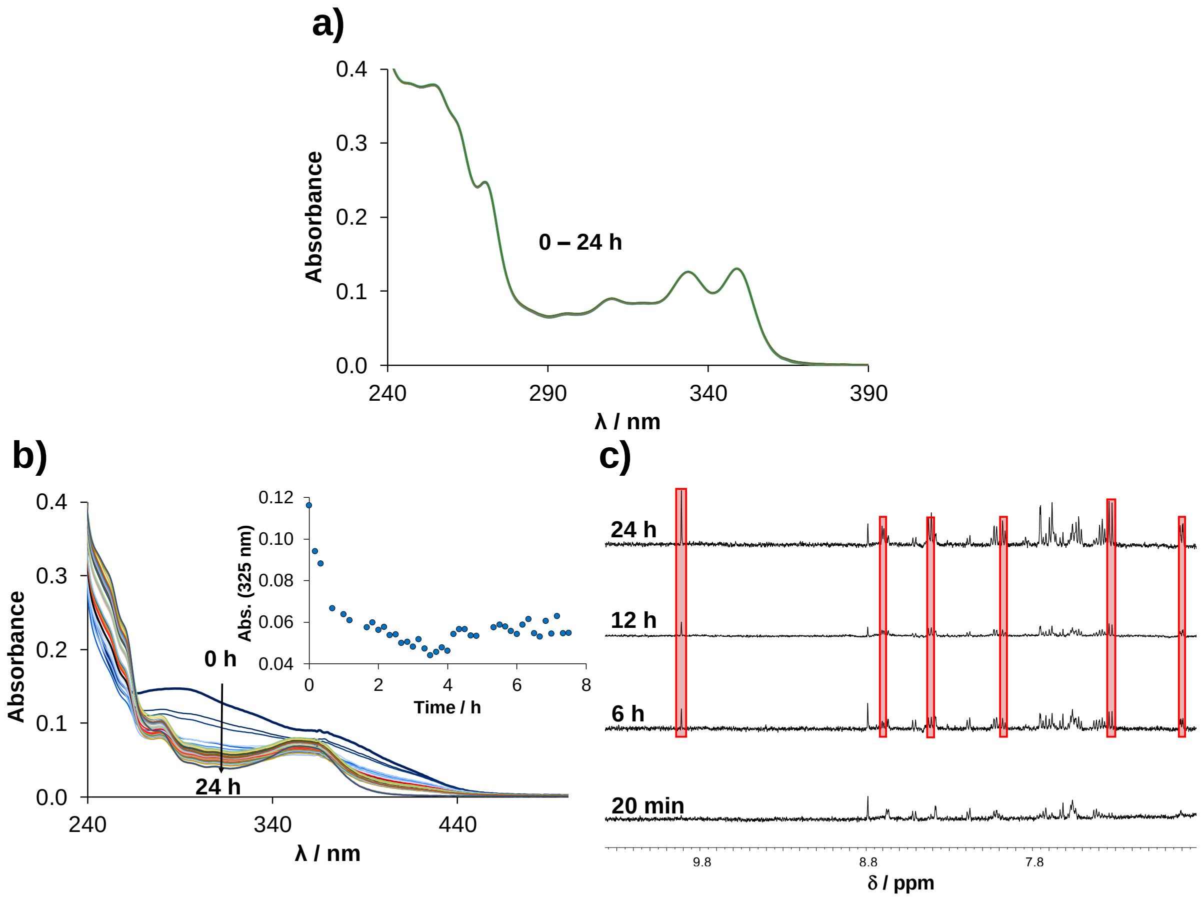


**Figure S41.** UV-vis spectra of IQPMA a) in 0.1 M HCl (pH = 1.0) and b) in PBS' buffer (pH = 7.4), followed over time. The spectral changes also indicate the appearance of precipitate. c) $^{1}$H NMR spectra of IQPMA in PBS' buffer (pH = 7.4) followed over time. Precipitate formation was observed. Red rectangles indicate the appearance of new peak set. {$c_{\mathrm{IQPMA}}$ = 10 (UV-vis) and 300 μM ($^{1}$H NMR); $\ell$ = 1 cm (UV-vis), 1% (*v/v*) DMSO/PBS' (UV-vis) or 50% (*v/v*) DMSO-$d_6$/PBS' ($^{1}$H NMR), $T$ = 25.0 $^{o}$C}

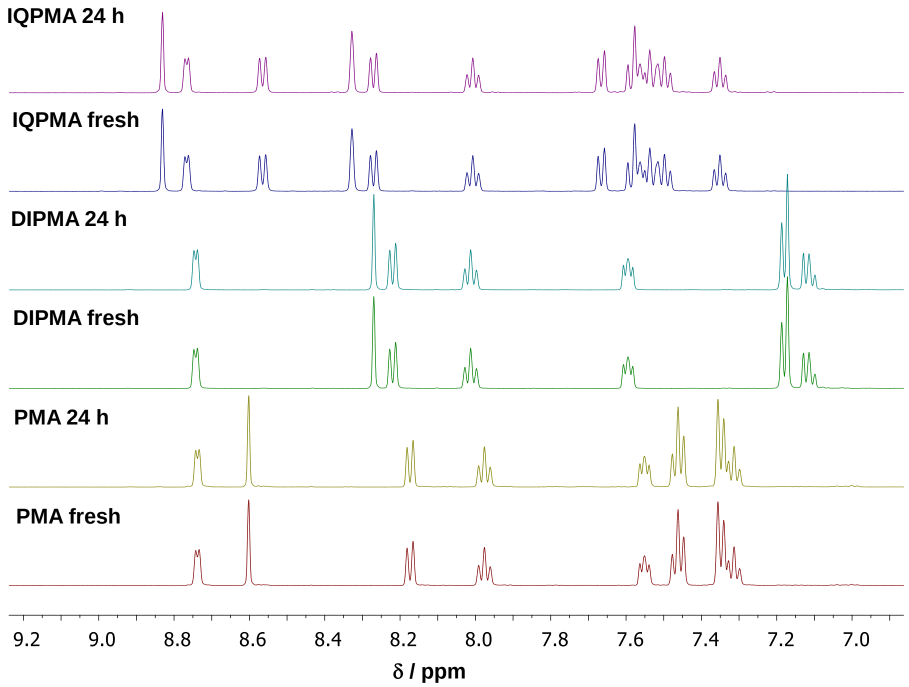


**Figure S42.** $^{1}$H NMR spectra of the stock solutions of the free ligands in the chemical shift range from 6.9 to 9.2 ppm: freshly prepared in DMSO-$d_6$ and after 24 h. {$c_{\text{ligand}}$ = 1 mM}

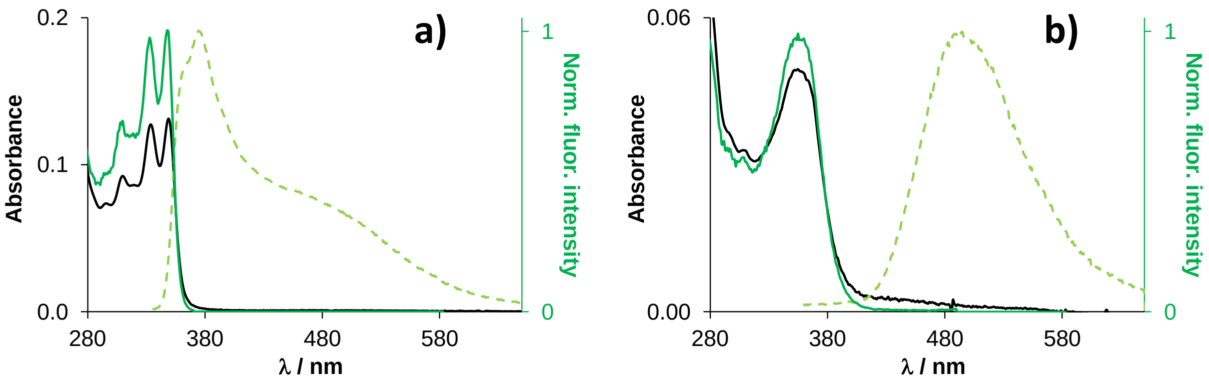


**Figure S43.** Fluorescence excitation (solid green) and emission (dashed green) spectra of IQPMA at (a) pH 1.0 and (b) pH 7.4 after 24 h waiting together with their UV-vis absorption spectra (black). {$c$ = 10 µM (a), 5 µM (b), 1% (*v*/*v*) DMSO/HCl solution or PBS'; $T$ = 25 °C}

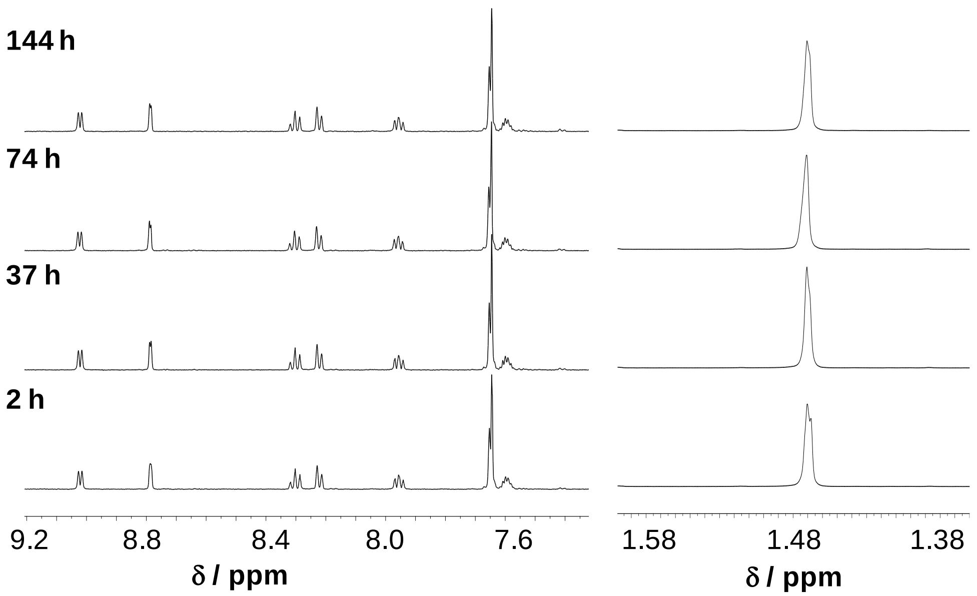


**Figure S44.** $^{1}$H NMR spectra of the RhCp* – PMA (1:1) system recorded at pH 1.0 at different time points. {$c_{PMA}$ = 200 µM; 10% (*v*/*v*) $D_2O/H_2O$; *I* = 0.10 M (KCl); *T* = 25.0 °C}

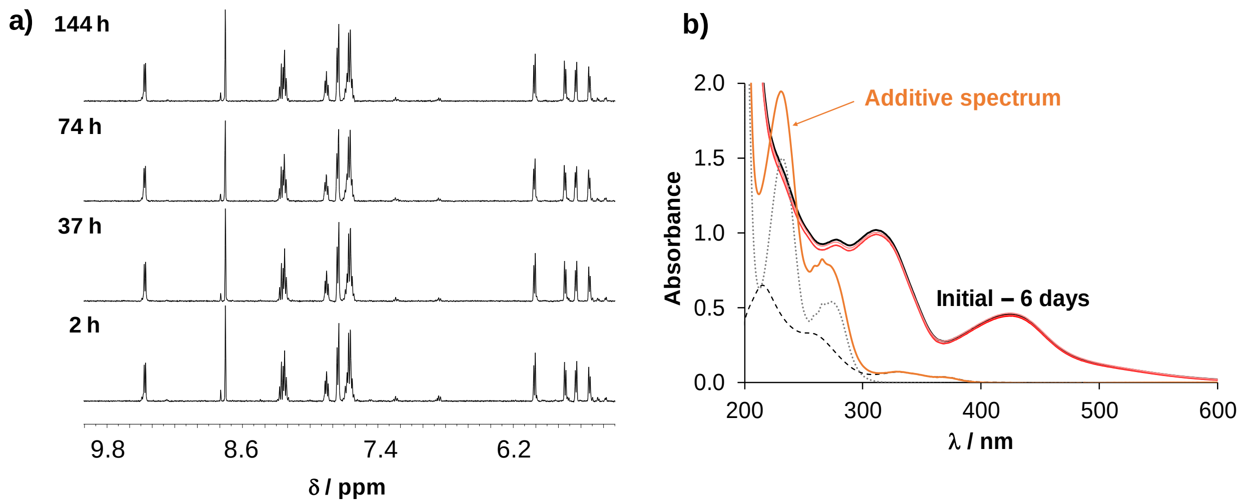


**Figure S45.** a) $^{1}$H NMR spectra of RuCym – PMA (1:1) system at pH 7.4 over time. {$c_{complex}$ = 200 µM; 10% (*v*/*v*) $D_2O$/PBS'; *T* = 25.0 °C} b) UV-vis spectra of OsCym – PMA (1:1) system at pH 7.4 over time. The spectrum of organometallic triaqua cation (black, dashed line), ligand (grey, dotted line) and their additive spectrum (orange, solid line) are also indicated. The great difference between the additive spectrum and the initial measured spectrum clearly shows the great extent of complex formation at this pH. {$c_{complex}$ = 125 µM, PBS'; $\ell$ = 1 cm, *T* = 25.0 °C}

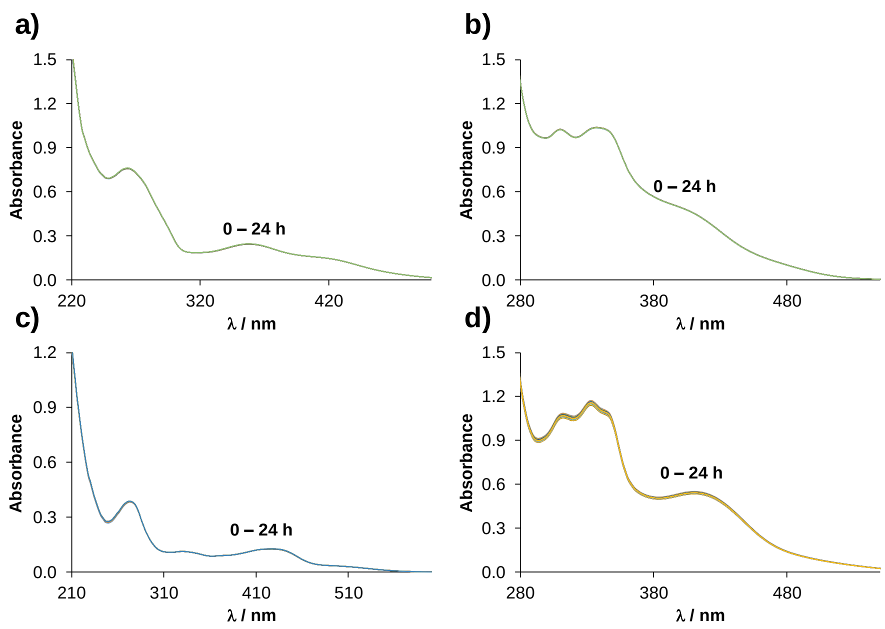


**Figure S46.** UV-vis spectra of a) [RuCym(DIPMA)Cl]Cl (**4a**), b) [RuCym(IQPMA)Cl]Cl (**1a**), c) [OsCym(DIPMA)Cl]Cl (**5a**) and d) [OsCym(IQPMA)Cl]Cl (**2a**) complex at pH = 1.0 (0.1 M HCl), followed over time. {$c_{complex}$ = 40 – 70 µM; $\ell$ = 1 cm, $T$ = 25.0 °C}

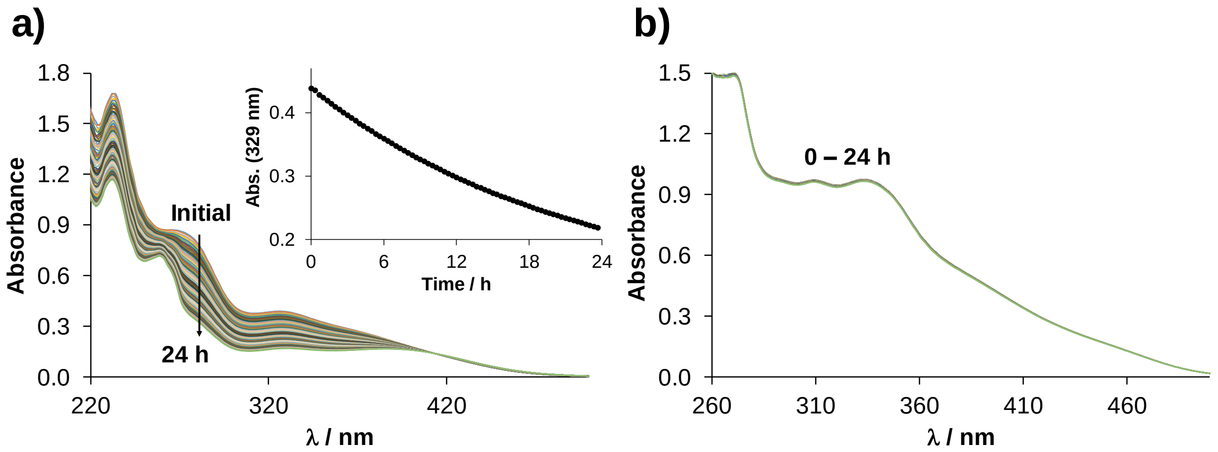


**Figure S47.** UV-vis spectra of a) [RhCp*(DIPMA)Cl]Cl (**6a**) and b) [RhCp*(IQPMA)Cl]Cl (**3a**) at pH = 1.0 (0.1 M HCl) followed over time. {$c_{complex}$ = 77 and 62 µM; $\ell$ = 1 cm, $T$ = 25.0 °C}

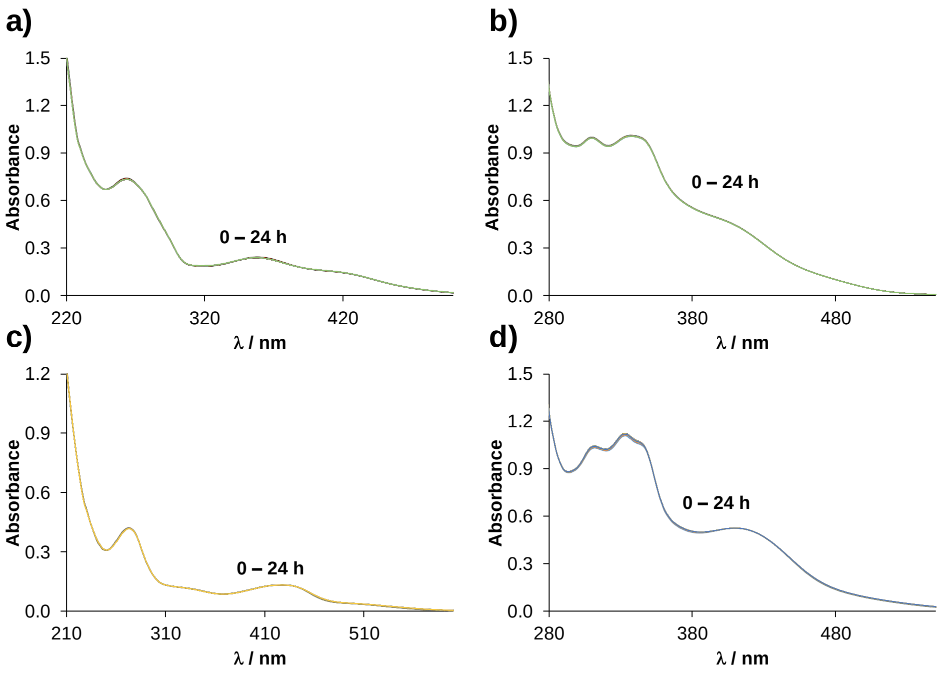


**Figure S48.** UV-vis spectra of a) [RuCym(DIPMA)Cl]Cl (**4a**), b) [RuCym(IQPMA)Cl]Cl (**1a**), c) [OsCym(DIPMA)Cl]Cl (**5a**) and d) [OsCym(IQPMA)Cl]Cl (**2a**) complex in PBS' buffer at pH = 7.4, followed over time. {$c_{complex}$ = 40 – 70 μM; $\ell$ = 1 cm, $T$ = 25.0 °C}

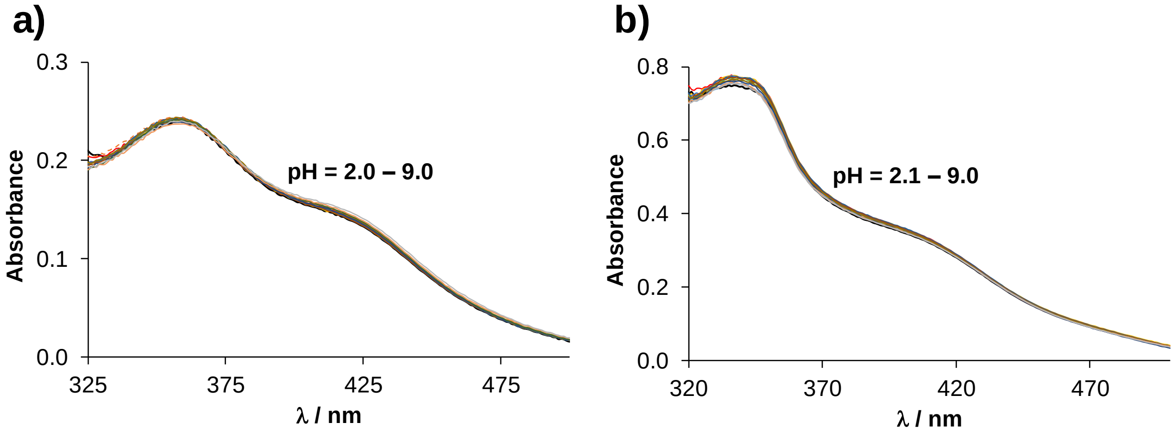


**Figure S49.** UV-vis spectra recorded for a) [RuCym(DIPMA)Cl]Cl (**4a**) and b) [RuCym(IQPMA)Cl]Cl (**1a**) complex at increasing pH values in $H_2O$. {$c_{complex}$ = 50 μM, $I$ = 0.2 M $KNO_3$, $\ell$ = 1 cm, $T$ = 25.0 °C}

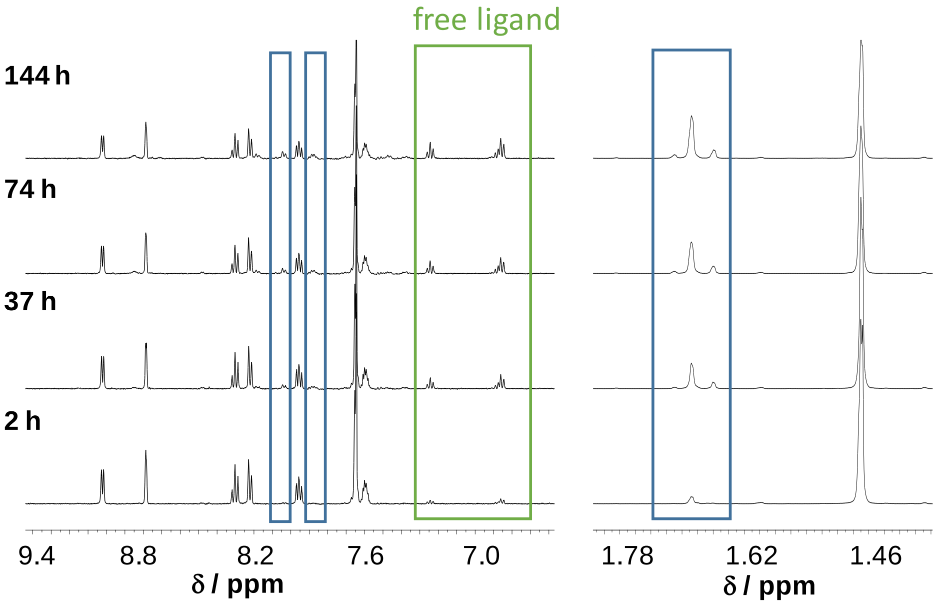


**Figure S50.** $^{1}H$ NMR spectra of the RhCp* – PMA (1:1) system recorded at pH 7.4 at different time points. The framed peaks appeared as new signals. The peaks indicated as free ligand show its hydrolyzed form. {$c_{PMA}$ = 200 μM; 10% (*v*/*v*) $D_2O$/PBS'; *T* = 25.0 °C}

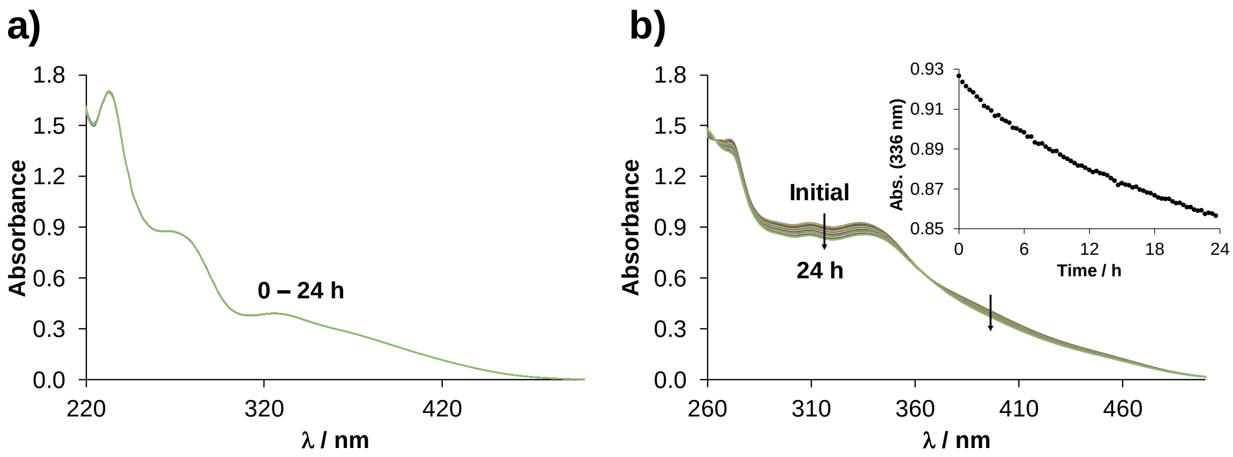


**Figure S51.** UV-vis spectra of a) [RhCp*(DIPMA)Cl]Cl (**6a**) and b) [RhCp*(IQPMA)Cl]Cl (**3a**) in PBS' buffer at pH = 7.4 followed over time. Insets show the absorbance changes as a function of time. {$c_{complex}$ = 77 and 62 µM; $\ell$ = 1 cm, $T$ = 25.0 °C}

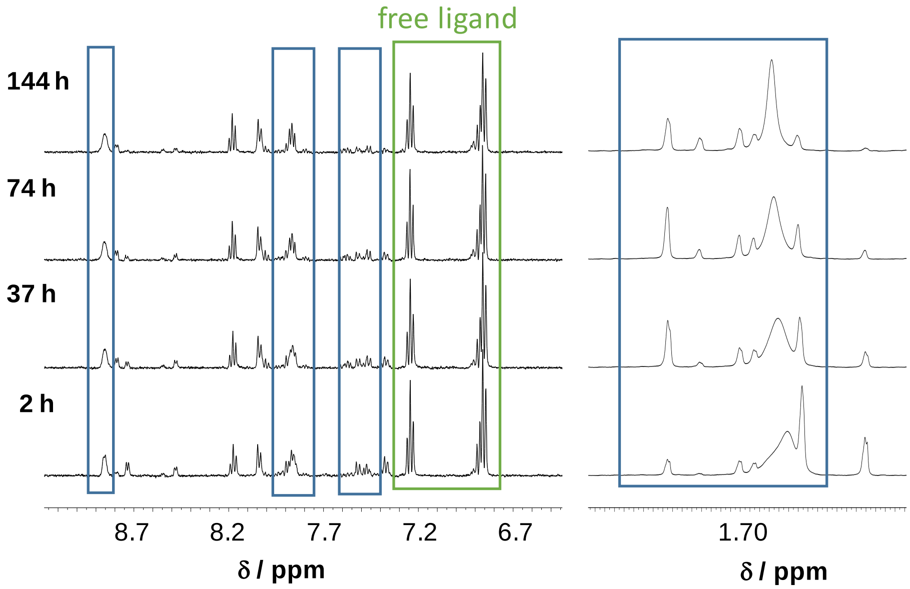


**Figure S52.** $^{1}$H NMR spectra of the RhCp* – PMA (1:1) system recorded at pH 9.4 at different time points. The framed peaks appeared as new signals. The peaks shown as free ligand indicate its hydrolyzed form. {$c_{PMA}$ = 200 µM; 10% ($v/v$) $D_2O/H_2O$; $T$ = 25.0 °C}

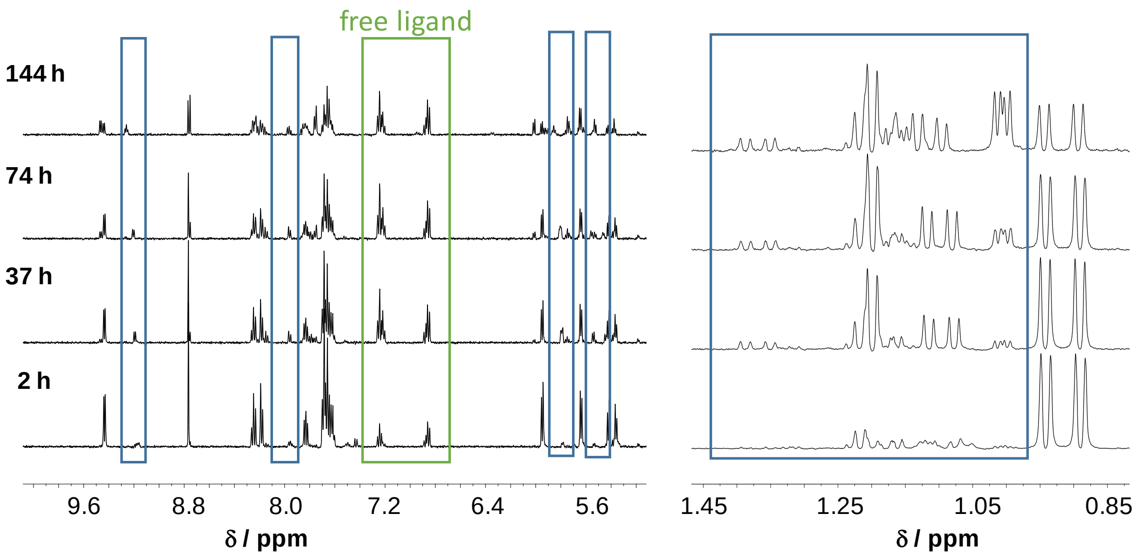


**Figure S53.** $^{1}$H NMR spectra of the RuCym – PMA (1:1) system recorded at pH 9.4 at different time points. The framed peaks appeared as new signals. The peaks shown as free ligand indicate its hydrolyzed form. {$c_{PMA}$ = 200 μM; 10% (*v*/*v*) $D_2O/H_2O$; *T* = 25.0 $^{o}$C}

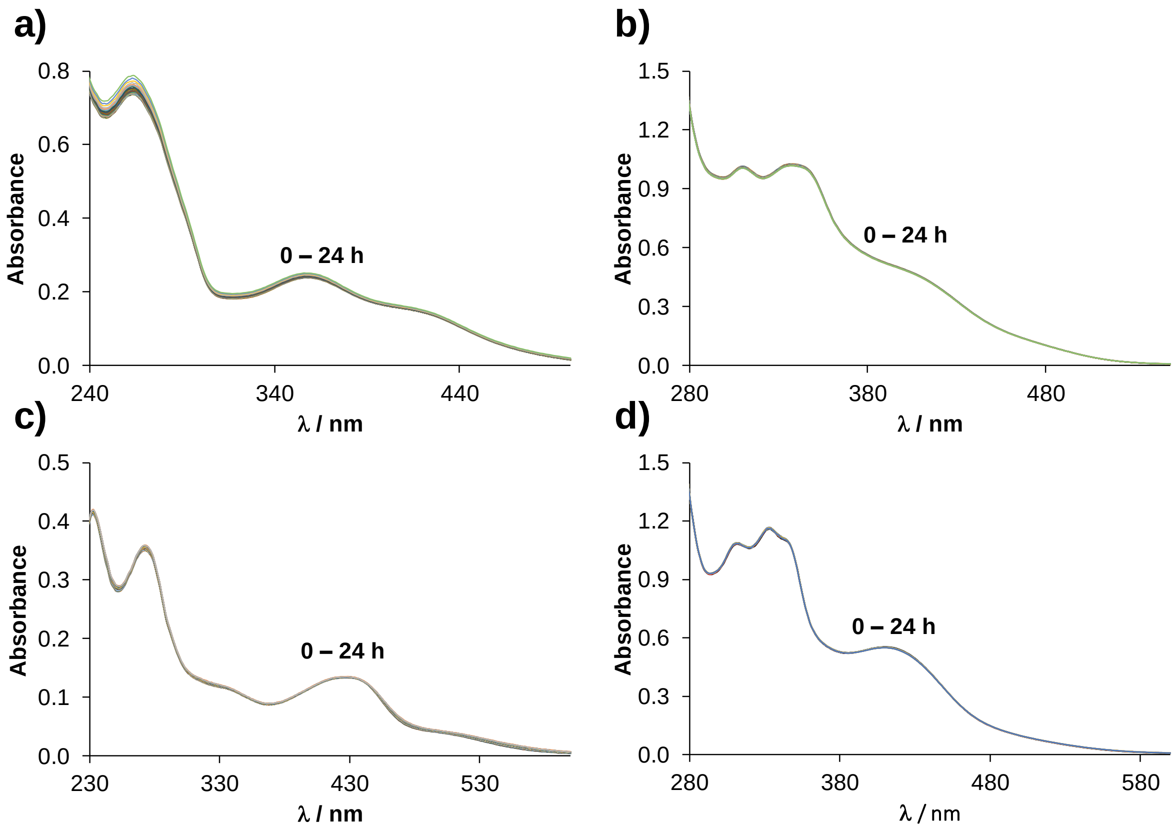


**Figure S54.** UV-vis spectra of a) [RuCym(DIPMA)Cl]Cl (**4a**), b) [RuCym(IQPMA)Cl]Cl (**1a**), c) [OsCym(DIPMA)Cl]Cl (**5a**) and d) [OsCym(IQPMA)Cl]Cl (**2a**) complex in Eagle's Minimum Essential Medium (EMEM) medium (with 1% (*v*/*v*) DMSO) followed over time. {$c_{complex}$ = 40 – 70 µM; $\ell$ = 1 cm, $T$ = 25.0 $^{o}$C}

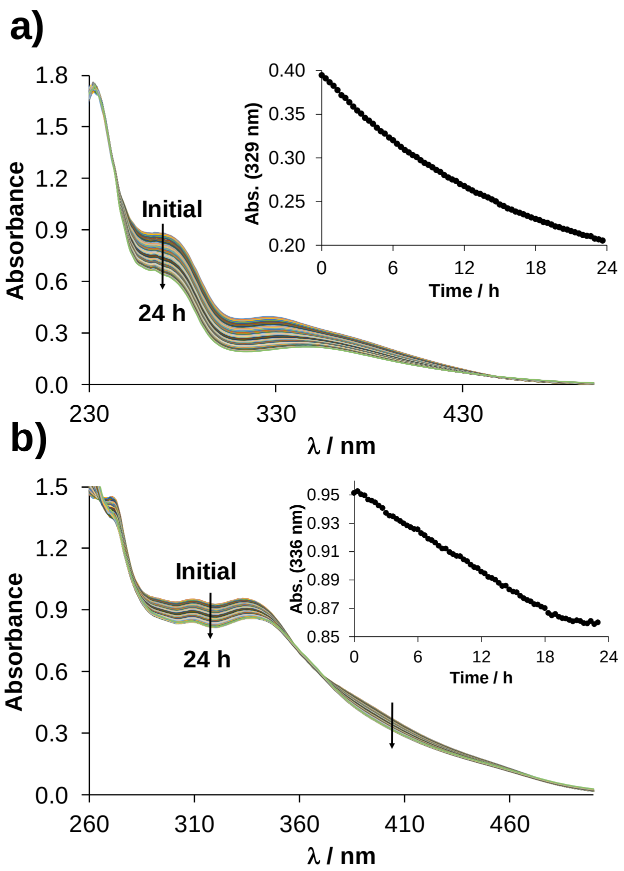


**Figure S55.** UV-vis spectra of a) [RhCp*(DIPMA)Cl]Cl (**6a**) and b) [RhCp*(IQPMA)Cl]Cl (**3a**)complex in Eagle's minimum essential medium (EMEM) (with 1% (*v*/*v*) DMSO), followed over time. {$c_{\text{complex}}$ = 77 and 62 µM; $\ell$ = 1 cm, $T$ = 25.0 $^{o}$C}

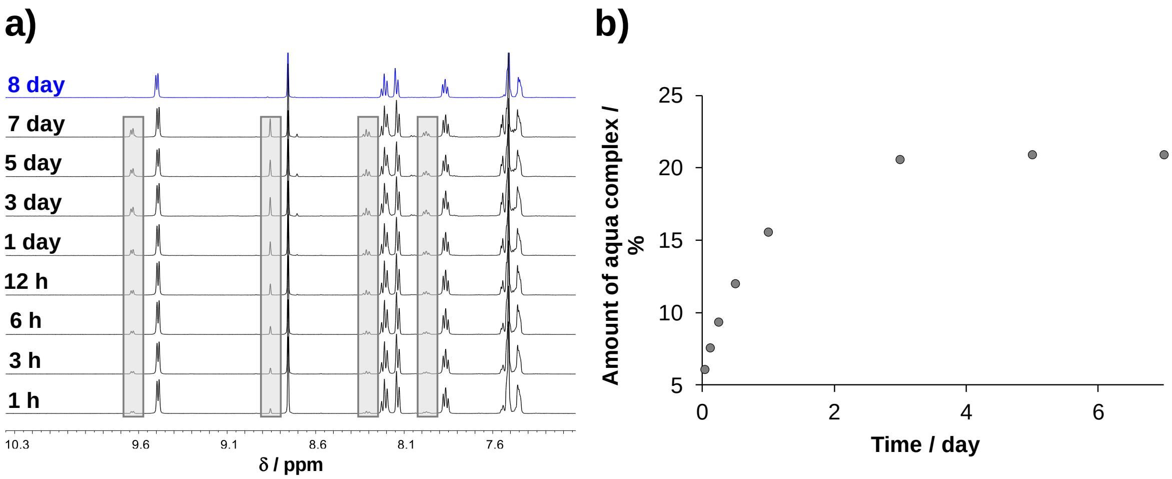


**Figure S56.** a) $^{1}$H NMR spectra in the low-field region recorded for **4a** complex followed over time. Grey rectangles indicate the appearance of peaks belonging to aqua complex. After seven days, an additional amount of KCl (+ ~5 equiv.) was added to the sample to enable the identification of the chlorido and aqua species; and the spectrum was recorded on the 8$^{th}$ day (blue spectrum). b) The amount (%) of aqua complex, calculated by integration of the corresponding peaks belong to aqua and chlorido forms, plotted against time. {$c_{complex}$ = 6.26 mM, $c_{Cl^-}$ = 12.52 mM, pH = 6.0 (20 mM phosphate buffer), 10% (*v/v*) $D_2O/H_2O$, $T$ = 25.0 °C}

**Table S7.** Distribution coefficients ($D_{7.4}$) of the synthesized metal complexes and their corresponding ligands, determined at three different chloride concentrations. The phase separation was performed after 4 h (or 2 h for RhCp* complexes) partitioning. {*n*-octanol/20 mM phosphate buffer, pH = 7.4; $t$ = 25 °C}

| **log$D_{7.4}$** | **$c$(Cl$^-$) = 4 mM** | **$c$(Cl$^-$) = 24 mM** | **$c$(Cl$^-$) = 100 mM** |
|---|---|---|---|
| **IQPMA** | n.d. | n.d. | +2.70 ± 0.09 |
| **1a** | n.d. | n.d. | +1.19 ± 0.01 |
| **2a** | n.d. | n.d. | +1.65 ± 0.01 |
| **3a** | +0.36 ± 0.03 | +0.46 ± 0.02 | +0.62 ± 0.02 |
| **DIPMA** | n.d. | n.d. | +2.87 ± 0.09 |
| **4a** | n.d. | n.d. | +0.90 ± 0.01 |
| **5a** | n.d. | n.d. | +1.26 ± 0.02 |
| **6a** | +0.07 ± 0.05 | +0.27 ± 0.05 | +0.56 ± 0.06 |

**Determination of distribution coefficients**

The traditional shake-flask method was employed to determine the distribution coefficients of both the ligands and complexes in *n*-octanol/buffered aqueous solution (20 mM phosphate, pH = 7.4) at varying

chloride ion concentrations (4, 24, and 100 mM). UV-Vis spectrophotometry was utilized for the analysis. The compounds were dissolved in the buffered aqueous solutions, which had been pre-saturated with *n*-octanol. The *n*-octanol and aqueous phases were then gently mixed in different volume ratios: 1:60 for the ligands, and 1:12.5 for the RhCp* and RuCym complexes. The mixtures were incubated for 4 h (due to the decomposition of RhCp* complexes at pH = 7.4, only a 2 h incubation period was used for these cases) before phase separation. Following this, the UV-Vis spectrum of either the aqueous or *n*-octanol phase was recorded and compared to a reference spectrum. Distribution coefficients were calculated by the following equations (Eqs. 3, 4):

When the stock solution was made in the aqueous phase:

$$D_{pH} = ((A_{stock} / A_{aq}) - 1) \times V_{aq}/V_{n\text{-octanol}} \qquad \text{(Eq. 3)}$$

When the stock solution was made in the *n*-octanol phase:

$$D_{pH} = ((A_{n\text{-octanol}} / A_{stock}) / (1 - (A_{n\text{-octanol}} / A_{stock})) \times V_{aq}/V_{n\text{-octanol}} \qquad \text{(Eq. 4)}$$

where $A_{stock}$: absorbance of the stock solution; $A_{aq}$: absorbance of the aqueous phase after separation; $V_{aq}$: volume of the aqueous phase; $V_{n\text{-octanol}}$: volume of the *n*-octanol phase; $A_{n\text{-octanol}}$: absorbance of the *n*-octanol phase after separation.

## Preparation of PLGA-based nanoformulations

PLGA 50:50 (poly(D,L-lactide-co-glycolide, lactide:glycolide (50:50), $M_n$ =30,000-60,000 Sigma-Aldrich) or PLGA 75:25 (poly(D,L-lactide-co-glycolide, lactide:glycolide (75:25), $M_n$ = 66,000-107,000, Sigma-Aldrich) were used for the PLGA-based nanoformulations. The PLGA solution (10 mg/mL) was prepared in DMSO, and the solid compound (**1a**, **4a**) was dissolved in 1 mL PLGA solution to obtain a final concentration of 1 mg/mL. Then, this solution was added dropwise to 10 mL of aqueous Pluronic (PLUR, Pluronic® F-127, cell culture tested, Sigma-Aldrich), solution prepared in Milli-Q water at concentrations of 0.5, 1, and 2 mg/mL. The resulting dispersion was stirred overnight at room temperature. Afterwards, 8 mL of this dispersion was centrifuged at 1500 rpm for 10 min, and 5 mL of the supernatant was collected for further processing. A 3 mL aliquot of this supernatant was dialyzed against 500 mL of Milli-Q water using a cellulose-based dialysis membrane (width: 25 mm / 1.0 in.) for 4 h to remove residual solvent and unincorporated materials. Following dialysis, the resulting volume was 2 mL, which was subsequently lyophilized. During dialysis, the samples initially exhibited slight opalescence, which gradually decreased over time, resulting in visibly clearer dispersions. In some cases, partial adsorption of the complex onto the cellulose membrane was observed, as indicated by the yellow coloration of the membrane corresponding to the compound’s characteristic color, while the

dialyzed sample became transparent. The PLGA-based nanoformulations were characterized for the $d_H$ and $\zeta$ values similarly as the liposomes.

### Determination of drug loading in the PLGA-based nanoformulations

The drug loading efficiency (DL%) was calculated according to the following equation:

$$\text{DL\%} = \frac{\text{mass of encapsulated drug}}{\text{total mass of nanoparticles}} \times 100$$

The nanoformulations were lyophilized, and the resulting dry powders were subsequently dissolved in DMSO. The concentration of the incorporated compound was quantified spectrophotometrically with a calibration curve obtained for samples prepared under identical solvent conditions (Figure S57), using an Agilent Cary 3500 diode array spectrophotometer. The total mass of the nanoparticles was obtained directly from the initial sample weights used for lyophilization.

### Preparation and evaluation of PLGA-PLUR polymeric formulations

The polymer-based nanocarriers developed in this study were constructed from a polymeric matrix composed of PLGA. The PLGA matrix was stabilized using the nonionic surfactant PLUR, a synthetic block copolymer, forming a stable colloidal system in which PLUR acted similarly to micellar structures, preventing particle aggregation. PLGA is both biodegradable and biocompatible, hydrolyzing *in vivo* into lactic and glycolic acid – naturally occurring metabolic intermediates – making it an excellent candidate for biomedical drug delivery applications. PLUR block copolymers are FDA-approved excipients widely employed in pharmaceutical formulations to enhance solubility and stabilize biocompatible nanocarriers [SI-9]. From a formulation standpoint, PLGA plays a central role as a copolymer composed of lactic acid (PL) and glycolic acid (GA) units. The relative ratio of these monomers dictates the overall hydrophobic-hydrophilic balance of the polymer: an increased lactic acid content (*e.g.* PLGA 75:25) yields a more hydrophobic matrix, whereas a higher glycolic acid content (*e.g.* PLGA 50:50) increases hydrophilicity [SI-10].

Two different PLGA compositions, PLGA 50:50 and PLGA 75:25, were examined to evaluate their influence on DL%. The hydrodynamic diameter, zeta potential, and drug loading for the different complex-loaded systems are shown in Tables S8,S9.

**Table S8.** Physico-chemical characterization of PLGA(75:25)–based formulations stabilized by PLUR before and after dialysis. Parameters include hydrodynamic diameter ($d_H$), zeta potential ($\zeta$), and drug loading (DL%) for complex-loaded systems.

| | DMSO phase | | Milli-Q phase | *before dialysis* | | after dialysis | | |
|---|---|---|---|---|---|---|---|---|
| **sample** | **$c_{SUBSTANCE}$ (mg·mL$^{-1}$)** | **$c_{PLGA\ 75:25}$ (mg/mL)** | **$c_{PLUR}$ (mg/mL)** | ***$d_H$ ± SD (nm)*** | ***$\zeta$ ± SD (mV)*** | **$d_H$ ± SD (nm)** | **$\zeta$ ± SD (mV)** | **DL%** |
| **empty** | – | 10 | 0.5 | *289 ± 15* | *–13 ± 3* | **243 ± 2** | **–27 ± 2** | **–** |
| | | | 1 | *298 ± 11* | *–10 ± 1* | **261 ± 4** | **–29 ± 3** | |
| **4a** | 1 | 10 | 0.5 | *310 ± 10* | *–3 ± 1* | **258 ± 10** | **–29 ± 2** | **0.71** |
| | | | 1 | *298 ± 5* | *–3 ± 2* | **239 ± 7** | **–26 ± 2** | **0.33** |
| | | | 2 | *285 ± 6* | *–2 ± 2* | **236 ± 9** | **–27 ± 2** | **0.31** |
| **1a** | 1 | 10 | 0.5 | *279 ± 4* | *9 ± 2* | **260 ± 54** | **–26 ± 2** | **0.25** |
| | | | 1 | *290 ± 11* | *7 ± 4* | **249 ± 6** | **–33 ± 2** | **0.13** |
| | | | 2 | *282 ± 7* | *8 ± 3* | **250 ± 1** | **–26 ± 1** | **0.09** |

**Table S9.** Physico-chemical characterization of PLGA(50:50)–based formulations stabilized them by before and after dialysis. Parameters include hydrodynamic diameter ($d_H$), zeta potential ($\zeta$), and drug loading (DL%) for complex-loaded systems.

| | DMSO phase | | Milli-Q phase | *before dialysis* | | after dialysis | | |
|---|---|---|---|---|---|---|---|---|
| **sample** | **$c_{SUBSTANCE}$ (mg/mL)** | **$c_{PLGA\ 50:50}$ (mg/mL)** | **$c_{PLUR}$ (mg/mL)** | ***$d_H$ ± SD (nm)*** | ***$\zeta$ ± SD (mV)*** | **$d_H$ ± SD (nm)** | **$\zeta$ ± SD (mV)** | **DL%** |
| **empty** | – | 10 | 0.5 | *113 ± 1* | *-16 ± 4* | **91 ± 1** | **-24 ± 6** | **–** |
| | | | 1 | *114 ± 2* | *-17 ± 6* | **86 ± 5** | **-24 ± 4** | |
| **4a** | 1 | 10 | 0.5 | *277 ± 10* | *-2 ± 1* | **227 ± 5** | **-32 ± 1** | **–** |
| | | | 1 | *262 ± 20* | *-5 ± 2* | **224 ± 7** | **-33 ± 1** | **0.46** |
| | | | 2 | *263 ± 4* | *-7 ± 2* | **214 ± 7** | **-30 ± 1** | **0.42** |
| **1a** | 1 | 10 | 0.5 | *282 ± 16* | *4 ± 1* | **246 ± 6** | **-33 ± 1** | **0.24** |
| | | | 1 | *288 ± 5* | *4 ± 2* | **227 ± 26** | **-32 ± 1** | **0.18** |
| | | | 2 | *269 ± 14* | *5 ± 2* | **246 ± 8** | **-31 ± 2** | **0.11** |

In subsequent experiments, the drug concentration (1 mg/mL) and PLGA concentration (10 mg/mL) were kept constant, while the PLUR concentration was varied (0.5, 1, and 2 mg/mL). The

data revealed a clear trend: increasing PLUR concentration led to a progressive decrease in DL% (*e.g.* for **1a**: 0.25 → 0.13 → 0.09%). This behavior can be attributed to the competitive interfacial adsorption of the surfactant, which can hinder efficient drug incorporation into the polymer matrix. Additionally, excess PLUR may form micellar aggregates in the aqueous phase, partially sequestering the drug and further lowering the overall loading efficiency. Dialysis of the samples was performed in Milli-Q water for 4 h to purify the dispersions and to remove residual solvent and unbound PLUR molecules.

Following dialysis, a pronounced decrease in the hydrodynamic diameter was observed for both PLGA compositions (*e.g.* for PLGA 75:25 **1a**: 282 nm → 250 nm; for PLGA 50:50 **1a**: 269 nm → 246 nm). This reduction in particle size can be attributed to the purification effect of dialysis, which eliminates loosely associated aggregates and promotes the formation of a more compact and homogeneous colloidal population. The zeta potential values of all formulations stabilized around −30 mV, indicative of excellent electrostatic stabilization and confirming the efficient surface coverage provided by the PLUR copolymer. Such zeta potential values are generally considered sufficient to prevent aggregation through electrostatic repulsion, ensuring the long-term colloidal stability of the nanocarriers in aqueous environments.

Based on the measured size and stability data, the PLGA-based nanocarriers exhibit suitable colloidal characteristics to serve as potential delivery vehicles for anticancer metallodrugs. However, our results also revealed that the drug loading achieved with the PLGA-PLUR system remained suboptimal for effective therapeutic application. Despite the favorable physico-chemical properties and biocompatibility of the PLGA matrix, the limited drug incorporation efficiency constrains its potential in practical formulations.

**Table S10.** Photos of the transported liposomal dispersions through gel and remaining free substances on the gel obtained for complexes **1a** and **4a** at 100 and 300 μM in the lipid-based nanocarriers.

| complex | form | substance $c_{initial}$ (μM) and sample types | | | |
|---|---|---|---|---|---|
| | | **100 μM dispersion** | **100 μM gel** | **300 μM dispersion** | **300 μM gel** |
| **4a** | unformulated | 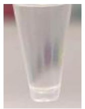 | 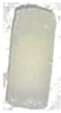 | 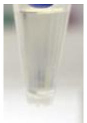 | 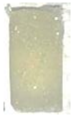 |
| | formulated | 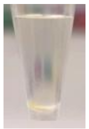 | 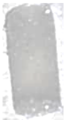 | 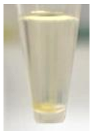 | 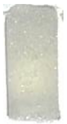 |
| **1a** | unformulated | 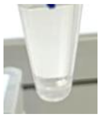 | 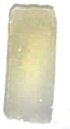 | 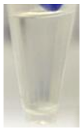 | 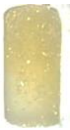 |
| | formulated | 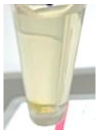 | 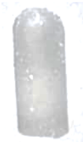 | 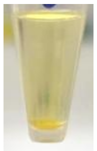 | 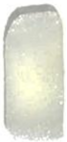 |

**Table S11.** Biofilm inhibition of MRSA ATCC 43300 and *Escherichia coli* ATCC 25922 by the investigated compounds. Biofilm formation was quantified by OD measurements at 600 nm. Inhibition (%) was calculated relative to untreated control (solution). "N.I." indicates no inhibition. Data are expressed as mean ± SD. Statistical significance was evaluated versus control (*$p < 0.05$; *$p < 0.05$, **$p < 0.01$, ***$p < 0.001$).

| | Compounds | $OD_{600}$ | SD +/- | Inhibition (%) | Statistics significant value: $p$<0.05 |
|---|---|---|---|---|---|
| **MRSA ATCC 43300 ($OD_{600}$: 2.12 +/- 0.19)** | **Solution** | 2.18 | 0.13 | N.I. | - |
| | **FEBA-RuCym (1a)** | 1.26 | 0.08 | 40.6 | ** $p = 0.002$ |
| | **FEBA-OsCym (2a)** | 0.71 | 0.04 | 66.3 | *** $p < 0.001$ |
| | **Compounds** | **$OD_{600}$** | **SD +/-** | **Inhibition (%)** | **Statistics significant value: $p$<0.05** |
| ***E. coli* ATCC 25922 ($OD_{600}$: 1.93 +/- 0.14)** | **Solution** | 2.11 | 0.12 | N.I. | - |
| | **FEBA-RuCym (1a)** | 1.37 | 0.12 | 29.1 | ** $p = 0.007$ |
| | **FEBA-OsCym (2a)** | 1.64 | 0.04 | 14.8 | * $p = 0.031$ |

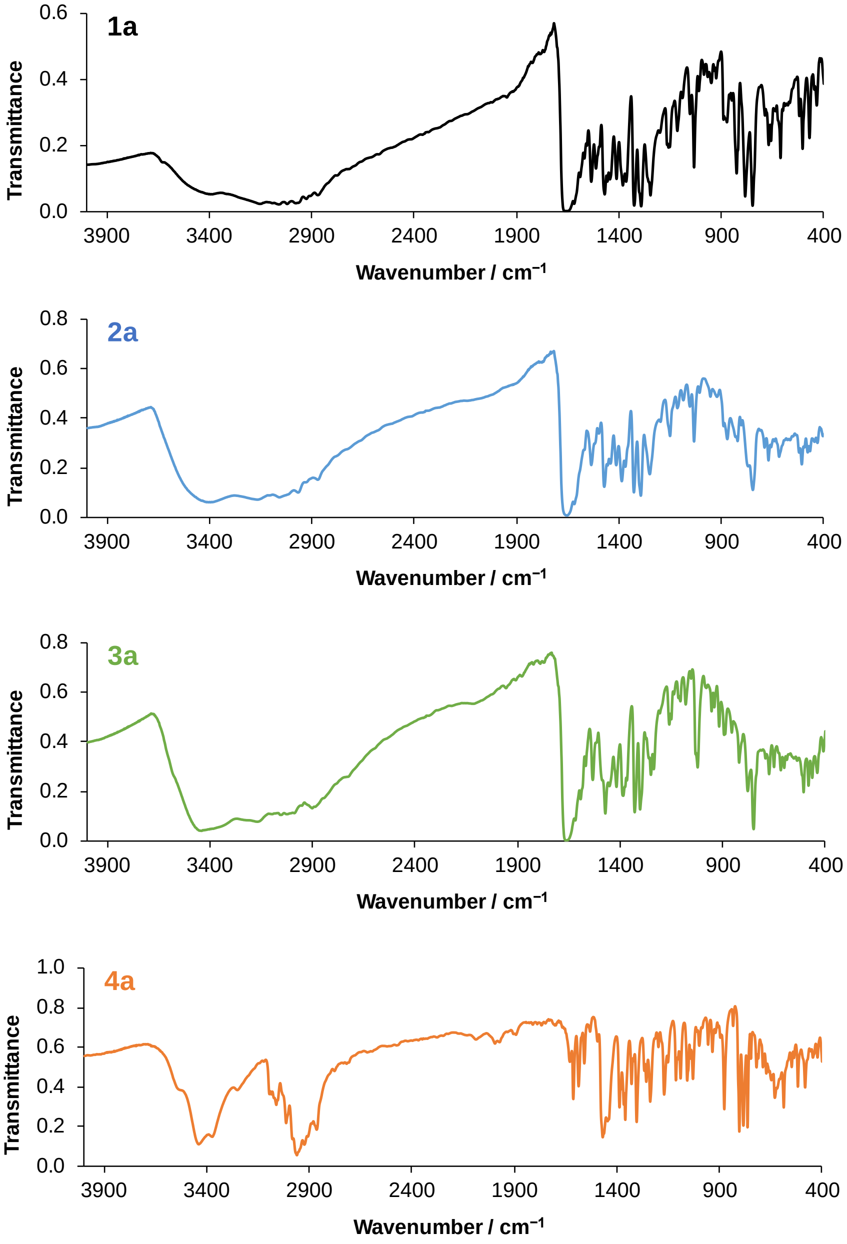


**Figure S57.** IR spectra of complexes **1a**−**4a**. The spectra display broad absorption bands in the 3600–3000 cm$^{-1}$ region, consistent with O−H stretching vibrations of water molecules, together with bands around 1600–1650 cm$^{-1}$ attributable to H−O−H bending vibrations. These observations support the presence of hydrated water in the isolated samples.

## EXPERIMENTAL FOR THE BIOLOGICAL ASSAYS

### *In vitro* cytotoxicity assays

*Cell lines and culture conditions.* Human colon adenocarcinoma cell lines Colo205 chemosensitive (ATCC-CCL-222), Colo320/MDR-LRP doxorubicin-resistant expressing ABCB1 (MDR1)-LRP (ATCC-CCL-220.1) and MCF-7 breast cancer and A549 (CCL-185) lung adenocarcinoma cell lines were purchased from LGC Promochem (Teddington, UK). The CCD-19Lu (CCL-210™) human normal fibroblast cell line was purchased from the American Type Culture Collection (ATCC). The Colo205 and Colo320 cells were cultured in RPMI 1640 medium supplemented with 10% heat-inactivated fetal bovine serum, 2 mM L-glutamine, 1 mM Na-pyruvate and 10 mM HEPES. The MCF-7, A549 and CCD-19Lu cell lines were cultured in EMEM supplemented with a non-essential amino acid (NEAA) mixture (Sigma-Aldrich, a selection of vitamins, 10% heat-inactivated fetal bovine serum (FBS), 2 mM L-glutamine (Sigma-Aldrich), 1 mM Na-pyruvate (Sigma-Aldrich), nystatin (Sigma-Aldrich) and a penicillin–streptomycin mixture (Sigma-Aldrich) in concentrations of 100 U/L and 10 mg/L, respectively. Cell lines were incubated at 37 °C, in a 5% $CO_2$, 95% air atmosphere. The semi-adherent human colon cancer cells were detached with Trypsin-Versene (EDTA, Sigma) solution for 5 min at 37 °C.

*MTT assay on the used human cancer and CCD-19Lu cells.* The tested compounds were dissolved in DMSO to prepare 10 mM stock solutions, which were diluted in complete culture medium, to study the effect of compounds on cancer cell growth. Lipid-based nanoformulations of complexes **1a** and **4a** (100 μM) were also tested on MCF-7 cells. Doxorubicin (Merck) was used as a positive control. The cells were treated with Trypsin-Versene (EDTA) solution. They were adjusted to a density of $1\times10^4$ cells in 100 μL of the appropriate culture medium and were added to each well, with the exception of the medium control wells. Except for the semi-adherent Colo205 and Colo320 cell lines, the other adherent cells were seeded 24 h prior to the assay. Then stock solutions

were diluted in the appropriate culture medium, and two-fold serial dilutions of compounds were prepared in 100 μL of the medium, horizontally. The final volume of the wells containing compounds and cells was 200 μL. The plates containing the cancer cells were incubated at 37 °C for 72 h; at the end of the incubation period, 20 μL of MTT solution (from a stock solution of 5 mg/mL) were added to each well. After incubation at 37 °C for 4 h, 100 μL of SDS solution (10% in 0.01 M HCI) were added to each well, and the plates were further incubated at 37 °C overnight. Cell growth was determined by measuring the optical density (OD) at 540/630 nm with a Multiscan EX ELISA reader (Thermo Labsystems, Cheshire, WA, USA). Inhibition of the cell growth (expressed as $IC_{50}$: inhibitory concentration that reduces by 50% the growth of the cells exposed to the tested compounds) was determined from the sigmoid curve where $100 - ((OD_{sample} - OD_{medium\ control})/(OD_{cell\ control} - OD_{medium\ control})) \times 100$ values were plotted against the logarithm of compound concentrations. Curves were fitted by GraphPad Prism software (2021, Graphpad Software, San Diego, CA, USA) [SI-17] using the sigmoidal dose-response model (comparing variable and fixed slopes). The $IC_{50}$ values were obtained from at least 3 independent experiments.

**Antibacterial activity assay**

*Escherichia coli* ATCC 25922 and *Klebsiella quasipneumoniae* ATCC 700603 Gram-negative strains were studied in the experiments. *Staphylococcus aureus* (ATCC 25928) reference strain, methicillin-resistant *Staphylococcus aureus* (MRSA 272123) and *Enterococcus faecalis* ATCC 29212 strains were used as Gram-positive strains. MIC values of compounds were determined in 96-well plates based on the Clinical and Laboratory Standard Institute guidelines (CLSI guidelines) [SI-18]. The measurements were performed in triplicate. The stock solutions of the compounds (dissolved in DMSO or 20% (*v/v*) DMSO/$H_2O$ mixture in 5 mM concentration) were diluted in 100 μL of Mueller Hinton Broth. Then $10^{-4}$ dilution of an overnight bacterial culture in 100 μL of

medium was added to each well, with the exception of the medium control wells. The plates were further incubated at 37 °C for 18 h; at the end of the incubation period, the MIC values of tested compounds were determined by visual inspection.

**Inhibition of biofilm formation**

The anti-biofilm effect of the tested compounds against bacteria strains was evaluated using the crystal violet (CV) assay. This dye (0.1% (*v*/*v*)) is used to detect the total biofilm biomass formed. Overnight cultures were diluted to OD of 0.1 at 600 nm in Luria-Bertani broth. The bacterial cultures were then added to 96-well microtiter plates, and the compounds were added at subinhibitory concentration (1/3 MIC). The final volume was 200 μL in each well. The microtiter plates were incubated at 30 °C for 48 h with gentle agitation (100 rpm). After the incubation period, medium was discarded, and the plates were washed with water to remove unattached cells. Then 200 μL CV was added to the wells and incubated for 15 min at room temperature. Then, CV was removed from the wells and the plates were washed again with water, and 200 μL of 70% (*v*/*v*) ethanol was added to the wells. Finally, the biofilm formation was determined by measuring the OD at 600 nm using Multiscan EX ELISA plate reader (Thermo Labsystems, Cheshire, WA, USA). The anti-biofilm effect of compounds was expressed as a percentage (%) reduction in biofilm formation. The biofilm inhibition (%) was calculated based on the mean of absorbance values. The analysis of data was performed using Graphpad Prism software [SI-17] applying the unpaired two-tailed t-test.

## REFERENCES

**[SI-1]** RAPID AUTO Ver. 3.1.1. (software) Rigaku/MSC Inc.: The Woodlands, TX, USA, 2016

**[SI-2]** Rigaku (2015). CrysAlisPro Software System, Version 1.171.38.41. Rigaku Oxford Diffraction, http://www.rigaku.com 2018 (accessed 2 February 2026)

**[SI-3]** Dolomanov, O.V.; Bourhis, L.J.; Gildea, R.J.; Howard, J.A.K.; Puschmann, H. OLEX2: a complete structure solution, refinement and analysis program. *J. Appl. Cryst.* **2009**, *42*, 339 – 341. DOI: 10.1107/S0021889808042726

**[SI-4]** Sheldrick, G.M. A short history of SHELX. *Acta. Cryst.* **2008**, *64*, 112 – 122. DOI: 10.1107/S0108767307043930

**[SI-5]** Bourhis, L.J.; Dolomanov, O.V.; Gildea, R.J.; Howard, J.A.K.; Puschmann, H. The anatomy of a comprehensive constrained, restrained refinement program for the modern computing environment – Olex2 dissected. *Acta. Cryst.* **2015**, *71*, 59 – 75. DOI: 10.1107/S2053273314022207

**[SI-6]** Macrae, C.F.; Edgington, P.R.; McCabe, P.; Pidcock, E.; Shields, G.P.; Taylor, R.; Towler, M.; van De Streek, J. Mercury: visualization and analysis of crystal structures. *J. Appl. Cryst*. **2006**, *39*, 453 – 457. DOI: 10.1107/S002188980600731X

**[SI-7]** Dhiman, R.; Nagaraja, C.M. Photochemical oxidation of water catalysed by cyclometalated Ir(iii) complexes bearing Schiff-base ligands. *New J. Chem.* **2019**, *43*, 13662 – 13669. DOI: 10.1039/C9NJ02281C

**[SI-8]** Yang, Y.; Ge, X.; Guo, L.; Zhu, T.; Tian, Z.; Zhang, H.; Du, Q.; Peng, H, Ma, W.; Liu, Z. Zwitterionic and cationic half-sandwich iridium(iii) ruthenium(ii) complexes bearing sulfonate groups: synthesis, characterization and their different biological activities. *Dalton Trans.* **2019**, *48*, 3193 – 3197. DOI: 10.1039/C9DT00259F

**[SI-9]** Li, J.J.; Guo, L.; Tian, Z.; Tian, M.; Zhang, S.; Xu, K.; Qian, Y.; Liu, Z. Novel half-sandwich iridium(iii) imino-pyridyl complexes showing remarkable in vitro anticancer activity. *Dalton Trans.* **2017**, *46*, 15520 – 15534. DOI: 10.1039/C7DT03265J

**[SI-10]** Deshmukh, G.; Gharpure, S.J.; Murugavel, R. Dinuclear Ru(II) Schiff Base Complex Catalyzed One-Pot Synthesis of Quinolines through Acceptorless Dehydrogenative Coupling of Secondary Alcohols with 2-Nitrobenzyl Alcohol. *Organometallics* **2024**, *43*, 1190 – 1202. DOI: 10.1021/acs.organomet.4c00129

**[SI-11]** Gichumbi, J.M.; Omondi, B.; Friedrich, H.B. Half-Sandwich Osmium(II) Complexes with Bidentate N,N-Chelating Ligands and Their Use in the Transfer Hydrogenation of Ketones. *EurJIC* **2017**, *2017*, 915 – 924. DOI: 10.1002/ejic.201601249

**[SI-12]** Hu. X.; Guo, L.; Liu, M.; Sun, M.; Zhang, Q.; Peng, H.; Zhang, F.; Liu, Z. Formation of Iridium(III) and Rhodium(III) Amine, Imine, and Amido Complexes Based on Pyridine–Amine Ligands: Structural

Diversity Arising from Reaction Conditions, Substituent Variation, and Metal Centers. *Inorg. Chem.* **2022**, *61*, 10051 – 10065. DOI: 10.1021/acs.inorgchem.2c00984

**[SI-13]** Mensah, S.; Rosenthal, J.D.; Dagar, M.; Brown, T.; Mills, J.J.; Hamaker, C.G.; Ferrence, G.M.; Webb, M.I. A Ru(ii)-arene-ferrocene complex with promising antibacterial activity. *Dalton Trans.* **2022**, *51*, 17609 – 17619. DOI: 10.1039/D2DT02696A

**[SI-14]** Thangavel, S.; Boopathi, S.; Mahadevaiah, N.; Kolandaivel, P.; Pansuriya, P.B.; Friedrich, H.B. Catalytic oxidation of primary aromatic alcohols using half sandwich Ir(III), Rh(III) and Ru(II) complexes: A practical and theoretical study. *J. Mol. Catal. A Chem.* **2016**, *423*, 160 – 171. DOI: 10.1016/j.molcata.2016.06.017

**[SI-15]** Wu, Y.; Che, F.-B.; Chen, J.-H. Synthesis and characterization of an amphiphilic pluronic-poly(D,L-lactide-co-glycolide) copolymer and their nanoparticles as protein delivery systems. *J. Appl. Polym. Sci.* **2008**, *110*, 1118 – 1128. DOI: 10.1002/app.28723

[**SI-16**] Dobhal, A.; Srivastav, A.; Dandekar, P.; Jain, R. Influence of lactide vs glycolide composition of poly (lactic-co-glycolic acid) polymers on encapsulation of hydrophobic molecules: molecular dynamics and formulation studies. *J. Mater. Sci: Mater Med.* **2021**, *32*, 126. DOI: 10.1007/s10856-021-06580-0

[**SI-17**] GraphPad Prism Version 7.00 for Windows. https://www.graphpad.com/ (accessed 2 February 2026).

[**SI-18**] CLSI. In Methods for Dilution Antimicrobial Susceptibility Tests for Bacteria that Grow Aerobically, tenth ed., P.J. Christopher, E.P. Polgar, (Eds.), Clinical and Laboratory Standards Institute: Wayne, MI, USA, 2015, 32, pp. 15 – 19.
